# Synthetic Method–Defect Correlations in Aldol Polycondensation: From Model Reactions to an *n*‑Type Thiazolothioazole Copolymer

**DOI:** 10.1021/jacs.6c07584

**Published:** 2026-07-24

**Authors:** Diego R. Hinojosa, Florian Günther, Paola Mantegazza, Matus Stredansky, Stefania Moro, Tobias Rüffer, Chunqiu Zheng, Yana Vaynzof, Giovanni Costantini, Michael Sommer

**Affiliations:** † Institute for Chemistry, 38869 Chemnitz University of Technology, Straße der Nationen 62, Chemnitz 09111, Germany; ‡ Instituto de Geociências e Ciências Exatas (IGCE), São Paulo State University (UNESP), Campus Rio Claro, Rio Claro 13506-700, Brazil; § School of Chemistry, 1724 University of Birmingham, Birmingham B15 2TT, U.K.; ∥ Chair for Emerging Electronic Technologies, Technical University of Dresden, Dresden 01187, Germany; ⊥ Leibniz-Institute for Solid State and Materials Research Dresden, Dresden 01069, Germany; # School of Physics and Astronomy, University of Birmingham, Birmingham B15 2TT, U.K.; ¶ Research Center MAIN, Chemnitz University of Technology, Rosenbergstraße 6, Chemnitz 09126, Germany

## Abstract

A new *n*-type conjugated polymer is prepared using aldol polycondensation. The backbone is designed to be linear, coplanar and to have a low LUMO energy level. A bisisatin monomer is made in four steps only under metal-free and ambient conditions. Key to the linear and coplanar structure is the bridging of two isatin motifs by a thiazolothiazole unit. Aldol copolymers with benzodifuranone afford polymers with low LUMO energy levels of ∼ −4.2 eV without using halogens. Defect formation during aldol polycondensation is studied quantitatively using monofunctional model compounds. Besides known defects such as homocouplings, fused analogs are observed along with other defects. Zinc stearate is identified as a superior catalyst, with defects being mostly absent in model reactions. This is in contrast to all other catalytic conditions used, including common literature protocols. Importantly, water must be continuously removed from the reaction to prevent the formation of defects. From these results, protocols are selected that lead to different defect densities in aldol copolymers. Spectroscopic methods confirm the defective nature of aldol polymers but offer limited structural insight into their nature and abundance. Electrospray deposition scanning tunneling microscopy enables direct identification and quantification of defect structures, revealing both similar trends and notable differences in their relative frequencies between small-molecule model systems and full polymers, rationalized by differences in reaction equilibria and chain mobility. Alongside introducing a new low LUMO *n*-type polymer synthesized via a metal-free route, this study opens pathways toward defect-free aldol polymers, which are otherwise intrinsically prone to defect formation.

## Graphical abstract

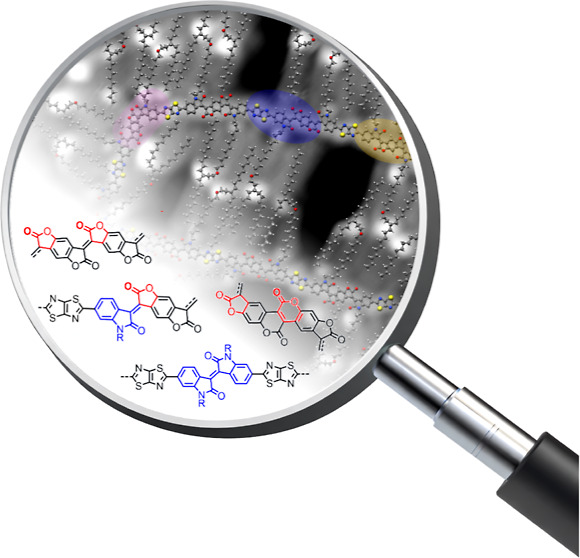

## Introduction

Conjugated polymers (CPs) play a pivotal role in the development of cost-effective, printable, and flexible electronic devices for technological applications spanning fields such as polymeric solar cells­(PSCs),^1^ organic electrochemical transistors (OECTs),^2^ and bioelectronics.^3^ Both *p*-type (hole-transporting) and *n*-type (electron-transporting) polymers are necessary for the construction and functionality of devices. While there has been tremendous progress in developing new materials with remarkable properties, the performance of *n*-type polymers notoriously lags behind that of their *p*-type counterparts. CPs are commonly synthesized by virtue of cross-coupling conjugated monomers to form extended conjugated backbones. The most commonly utilized cross-coupling variants are palladium- or nickel-catalyzed Stille,^4^ Suzuki^5^ and Kumada^6^ polycondensations. While these reactions offer excellent regioselectivity, they also have inherent drawbacks. For example, organotin compounds necessary for Stille couplings are neurotoxic.^7^ Kumada reactions using organomagnesium reagents are moisture-sensitive and must be performed in situ, and their scope is mostly limited to polythiophenes.^8^ Organoboron reagents^9^ for Suzuki reactions require bases, which lowers functional group tolerance. A leap forward in the investigation of synthetic alternatives for the preparation of conjugated polymers was achieved with the development of direct arylation polycondensation (DAP).^10–14^ This methodology is continually improved and extended to low-temperature protocol^15–17^ as well as to Cu as a catalyst,^18–20^ and the initially unknown role of defects in different optoelectronic properties has been largely clarified.^21–25^ While DAP does not require organometallic prefunctionalized building blocks and therefore profits from a higher atom economy and reduced waste, transition metals are still required as catalysts. Another improvement in this direction that has recently attracted interest is cross-dehydrogenative coupling.^26^ Here, only two substrates containing Ar–H bonds are required for the coupling to take place. However, clear limitations in selectivity and monomer scope require further effort to expand its applicability. Apart from the use of Ni in KCTP, all reactions mentioned above require a palladium catalyst. This is a clear drawback, as precious metals not only increase reaction costs but can also lead to metallic impurities, causing inferior device performance^27^ and necessitating additional purification steps. Finally, using transition metals often requires inert reaction conditions to prevent oxidation, further increasing costs and limiting scalability.

Aldol condensation is emerging as an attractive, transition metal-free alternative for the synthesis of CPs.^28,29^ In an aldol condensation, an α–CH bond next to a carbonyl group acts as a nucleophile, reacting with another carbonyl group as an electrophile. This reaction can be catalyzed by a base or an acid.^30^ The elemental steps in an aldol condensation are (i) acid/base enhancement of the nucleophilicity of an α–CH bond adjacent to an electron-withdrawing group via enol/enolate formation, respectively. (ii) Nucleophilic attack of this new species to an electrophilic center. If the electrophile is a ketone, an alcohol molecule is formed after protonation. (iii) Dehydration of the newly formed alcohol to render a double bond.^31^ Depending on the nature of the condensed product, the last step usually requires heating. One of the most common monomer combinations consists of isatin-based electrophiles and benzofuranone- or oxindole-based nucleophiles.^32–34^ This methodology was utilized, for example, to prepare a family of benzodifuranone-isatin based polymers with high mobilities in organic thin film transistors.^35^ The use of benzodifuranone as a nucleophile generates strongly electron-deficient backbones with low LUMO levels, which is fundamentally important for improving charge transport and air-stability of *n*-type polymers.^36–38^

Whereas aldol polycondensations have been reported to profit from high molar masses, high yields, and nontoxic starting materials, the synthetic pathways leading to these monomers either rely on long and cumbersome multistep syntheses^33,34,39^ or on the use of palladium-catalyzed cross-coupling reactions to bridge two isatin units.^40,41^ In this context, the high-performance homopolymer PBDFO^38,42^ has also been prepared via aldol condensation, albeit with highly toxic selenium dioxide as an in situ oxidant.^43^ However, while PBDFO is an intriguing material, processability is limited, and tuning its energetic levels is inherently difficult due to its homopolymer nature.

With these recent developments, the structural integrity of CP backbones made by aldol condensations has moved into focus. Analyzing molecular defects of CPs is generally cumbersome, as model reactions, high temperature NMR spectroscopy, as well as combinations of various analytical methods are required.^22,23,44–47^ While aldol polycondensation is sometimes considered to produce defect-free CPs, a scanning tunneling microscopy (STM) study has recently shown that certain defects appear in these polymers as a result of side reactions, including electrophile and nucleophile homocouplings as well as geometrical misscouplings.^48^ Since defects are generally critical for the optoelectronic properties of CPs and thus device performanc,^44,49,50^ an in-depth study of their origins during aldol polycondensations, as well as strategies for their elimination, is of major importance.

This work contributes to the field of aldol *n*-type CPs in several ways. First, we design a streamlined, four-step reaction pathway to a new bisisatin monomer. In this monomer, two simple isatin motifs are linked by an electron-deficient thiazolo­[5,4-*d*] thiazole (TzTz) unit. The resulting monomer backbone is linear and coplanar due to the bridging TzTz, which provides steric relief from the neighboring isatin through nitrogen atoms. Furthermore, the presence of imine-type nitrogens deepen the lowest unoccupied molecular orbital (LUMO) energy level without the necessity for extra substituents such as halogens.^51^ Moreover, the monomer synthesis is transition metal-free and proceeds under open-flask conditions, thus avoiding the use of sensitive organometallic reagents that undermine the general advantages of aldol polycondensations. The resulting polymers with benzodifuranone (BDF) as comonomer feature a deep LUMO level of −4.2 eV and are processable from common organic solvents. Furthermore, we also study defect formation using model reactions of monofunctional substrates under a broad range of catalytic conditions. Several side products are identified and quantified by chromatographic separation, establishing the structural basis for their formation in polymer backbones during aldol polycondensation. A wide variety of defects is observed, including homocouplings, fused isomers, nonconjugated defects, as well as *cis* couplings. Their formation is strongly dependent on the catalytic conditions. The lowest defect level is achieved using zinc stearate as a catalyst, but only when water is continuously removed from the reaction mixture. Based on the model reactions, selected conditions are applied to polycondensations, and spectroscopic signatures of well-defined and defect-enriched polymers are established. Finally, electrospray deposition scanning tunneling microscopy (ESD-STM) is employed to directly identify and quantify defect structures in full polymer chains. This confirms several of the defects inferred from model reactions, with their quantification revealing both similar trends for certain defect classes but notable differences in their relative frequencies for others. These differences are discussed in terms of variations in reaction equilibria between small-molecule model systems and polymerizations, as well as the reduced mobility of the growing polymer chains compared to small molecules.

## Results and Discussion

### (Retro)­Synthesis of the Bisisatin Monomer

The construction of monomers containing two terminal isatins provides a scaffold for metal-free aldol polycondensation with BDF as comonomer, enabling access to *n*-type aldol CPs with optoelectronic properties that depend on the bisisatin structure.^32^ To streamline the reaction sequence, eliminate the need for transition metals, and design a coplanar backbone with a low LUMO energy, we selected the heteropentalene thiazolo­[5,4-*d*]­thiazole (TzTz) as a motif to bridge two isatin units. TzTz motifs and their bithiazole derivatives have been utilized to achieve polymers with deeper LUMO levels and reduced sterical hindrance.^53–57^ Compared to isostructural thienothiophene (TT), the removal of two hydrogens in TzTz eliminates sterical hindrance and with the neighboring phenyl rings of the isatin fragments, thus enabling a coplanar backbone.^52^

The torsion potentials of the TzTz-isatin fragment as well as the isostructural TT analog calculated by density functional theory (DFT) confirmed this assumption (Scheme 1a left). While a significant dihedral angle between TT-isatin remained, TzTz-isatin was essentially coplanar. This was also seen by the geometry-optimized gas phase structure of the repeat unit as shown in Scheme 1a right (For more details See the Supporting Information and Figure S1). The dihedral angles between isatin and BDF were also low, albeit not 0°, in the gas phase, as noted earlier for these polymers.^58^

**Scheme 1:**
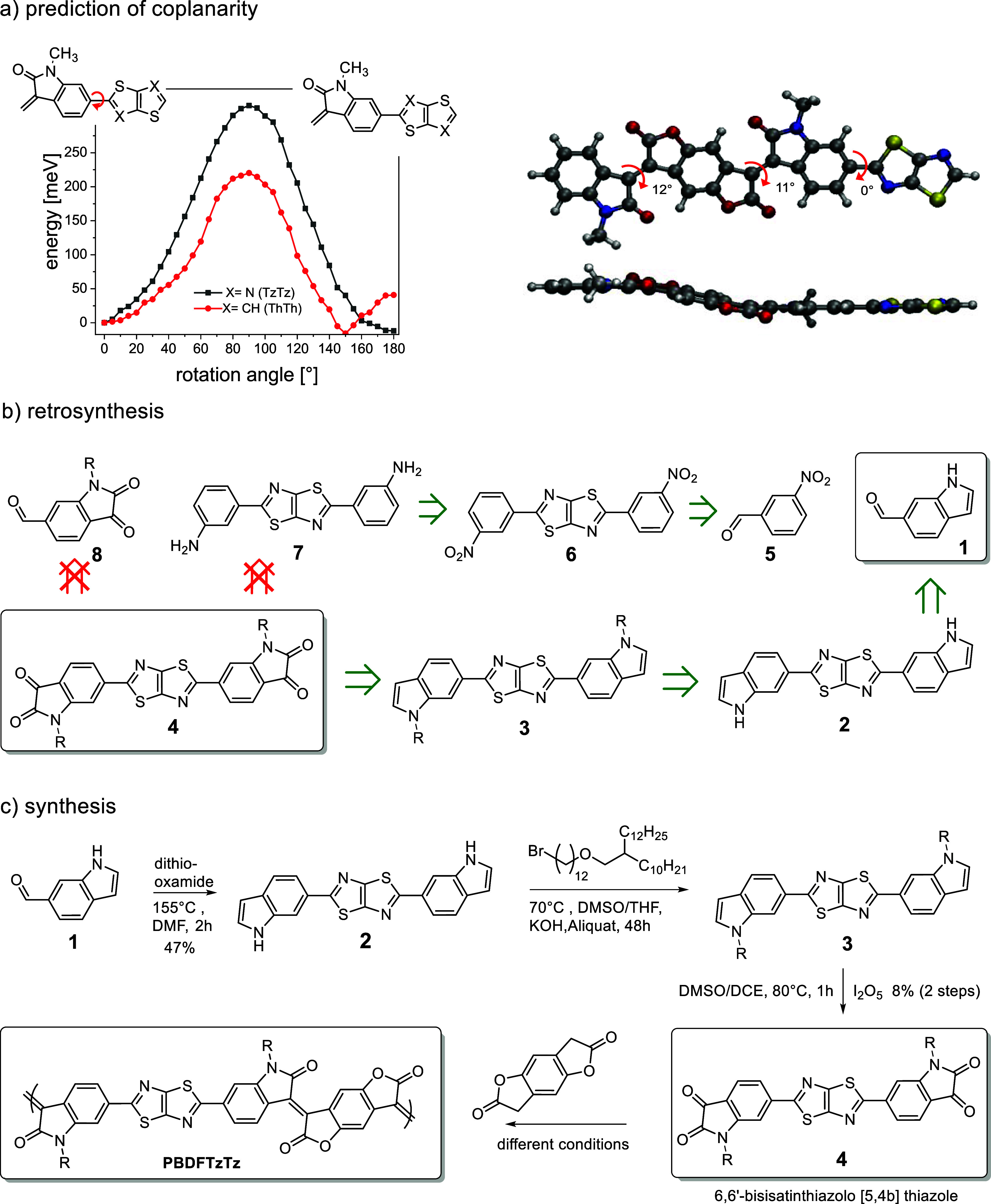
(a) Torsion Potential of the Thiazolothiazole-Isatin Bond and Geometry-Optimized Repeat Unit as Obtained from DFT/PBE/DZVP Simulations; (b) Retrosynthetic Analysis of the Monomer 6,6′-Bisisatinthiazolo [5,4-*d*] Thiazole **4** and; (c) Synthesis and Polycondensation of **4** with Benzodifuranone

Upon retrosynthetic analysis of **4 (** Scheme 1 **b)**, various attempts were made that were not successful (see Supporting Information for details). The successful synthetic route starts with indoles as precursors anticipated to be transformed into isatin derivatives by direct oxidation. Along this line, 6,6′-bisindolethiazolo­[5,4-*d*] thiazole **2** was obtained by simply treating commercially available 6-formyl indole **1** with 0.3 equiv of dithiooxamide in DMA for 2 h at 155 °C (Scheme 1c). The addition of cold ethanol led to the precipitation of a bright golden powder with a strong, shiny appearance under UV light, indicative of a planarized D-A-D system containing TzTz.^59^ This new molecule exhibited low solubility in common organic solvents due to its extended aromatic system, the possibility for hydrogen bonding, and the absence of aliphatic side chains. *N*-alkylation using potassium hydroxide in DMSO, along with a phase transfer catalyst, furnished **3** having single oxygen branched side chains, as previously developed to efficiently solubilize benzodifuranone-isatin copolymers.^60,61^ This intermediate showed high solubility in chlorinated solvents and was obtained in sufficient purity without column chromatography. Finally, **3** was oxidized to the final 6,6́-bisisatin­[5,4-*d*]­thiazolothiazole monomer **4** using iodine pentoxide in DMSO in 8% yield over two steps.^62^ Monomer **4** was purified by Soxhlet extraction with methanol, followed by column chromatography.

Among potential reasons for the low yield is the self-condensation of isatin units during oxidation, leading to oligomeric isoindigo derivatives. Notably, other oxidation protocols, such as pyridinium chlorochromate and aluminum chloride, also exhibit poor selectivity and require strict control of reaction conditions to avoid isatin homocoupling.^63,64^ In our hands, iodine pentoxide was the only benign oxidant that enabled this transformation.^62^ Other oxidants, such as Cu/TBPH,^65^ I_2_/TBPH,^66^ NBS^67^ and I_2_ ^63^_,_ were ineffective in delivering any product. Since I_2_O_5_ is believed to require DMSO as co-oxidant in a Kornblum-type reaction,^63^ potential improvements in yield might involve using smaller amounts of DMSO to enhance the miscibility between the highly polar DMSO and the alkylated monomer, or employing alternative cosolvent/DMSO systems. Importantly, **4** was synthesized without the need for metals or inert reaction atmospheres, providing a simple and straightforward synthetic approach. All products were characterized via NMR spectroscopy and MALDI ToF mass spectrometry (MS), see Supporting Information.

### Model Reactions and Defect Analyses

With monomer **4** in hand, we proceeded toward its polycondensation with BDF. Aldol polycondensations are often expected to yield linear polymers.^68^ However, defects are well-known to occur in many conjugated polymers synthesized via a variety of coupling reactions, even when polymerization proceeds through well-defined reactive sites.^47,49,50,69^ Recent direct STM imaging of aldol polymers revealed the presence of both sequence and coupling defects in these systems,^48^ highlighting that the nature and occurrence of defect formation remain incompletely understood and warrant further investigation. It is also important to note that spectroscopic analysis of defects in BDF-based aldol polymers via ^1^H NMR is not possible due to extremely broad signals arising from strong aggregation, which persists even at elevated temperatures. Thus, we opted for model reactions of a 1:1 mixture of monofunctional benzofuranone **9** and *N*-methyl-isatin **10** in toluene (Scheme 2a). We screened a variety of conditions and catalysts (A-O in Table 1). To obtain comparable information, we separated reaction mixtures consistently using an optimized protocol for automated column chromatography and analyzed all fractions via NMR spectroscopy and MALDI ToF MS (see Supporting Information).

**Scheme 2:**
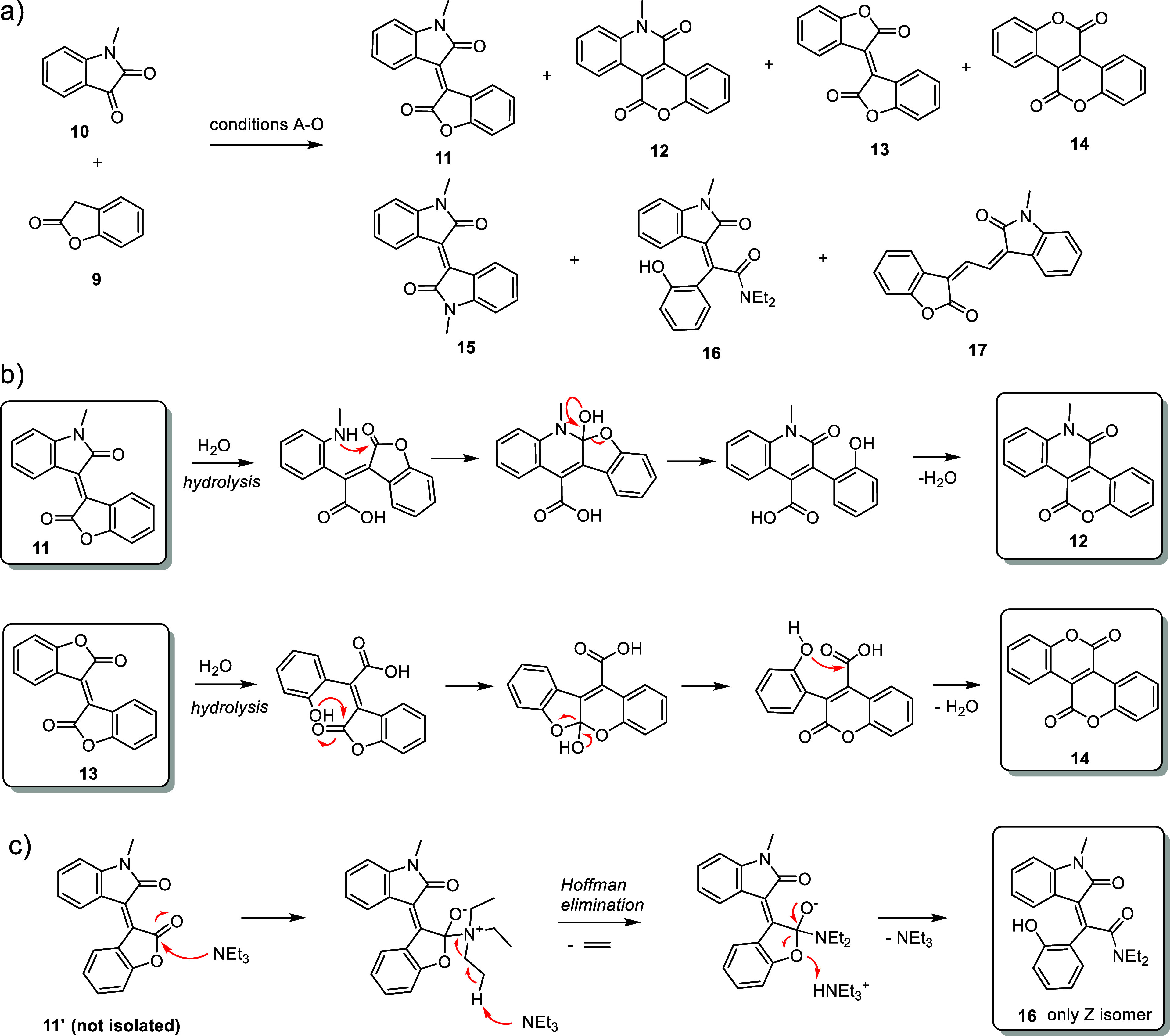
(a) Isolated Compounds **11**–**17** from the Model Aldol Reaction of **9** and **10**; (b) Possible Reaction Pathways to Compounds **11**–**15**; (c) Formation of Compound **16** When Using Triethylamine as Catalyst (conditions E in Table 1)

**Table 1:** Summary of the Conditions and Product Distribution of the Model Aldol Reaction of 9 and 10^a^

conditions^b^	catalyst	yield 11%	yield 12%	yield 13%	yield 14%	yield 15%	rest^c^ [mg]
A^d^	*p*TsOH	82	1	trace	1	trace^e^	117.6
B	*p*TsOH	78	trace^e^	trace^e^	1	2	101.3
C^f^	*p*TsOH	73	4	1	1	1	160.0
D	HCl	24	0	trace^e^	2	0	442.3
E	TEA	60	trace^e^	1	3	2	172.4
F	Zn(st)_2_	86	1	trace^e^	trace^e^	2	91.8
G	Mg(st)_2_	67	1	1	4	11	365.4
H	Zn(st)_2_+ 4Å MS	82	2	5	0	8	142.1
I	Zn(st)_2_ + 4 Å MS (spatially separated)	>95%	0	trace^e^	trace^e^	n.q^g^	n.q^h^
J	4 Å MS	47	0	n.q^g^	n.q^g^	0	385.0
K	none	33	0	3	trace^e^	n.q^g^	467.0
L	Li_2_CO_3_	66	trace^e^	2	2	2	193.5
M	Na(OAc)	57	20	0	3	16	291.6
N	K(OAc)	38	2	0	12	5	205.3
O	Zn(OAc)_2_	74	4	0	2	11	15.3

a Yields were determined by weighing pure fractions and additionally, if needed, integration of NMR spectra of mixed fractions. Other intermediates of unknown nature account for the remainder of 100% yield of each reaction. *p*TsOH: *para*-toluene sulfonic acid, Zn­(st)_2_: zinc stearate, Mg­(st)_2_: magnesium stearate, MS: mol sieve.

b Standard conditions unless otherwise noted: ambient atmosphere, concentration and scale of **9** and **10** was 0.25­[M], ratio of **9**:**10** = 1:1, 0.2 eq. of catalyst.

c Remainder of solid material obtained after applying a standardized protocol for column chromatography.

d Argon atmosphere.

e Trace: <0.5%.

f Reaction performed in the absence of light.

g Not quantified, NMR showed low intensities close to the baseline and the presence of different byproducts made quantification unprecise.

h The majority of this fraction was cross-aldol product **11**.

All isolated and unambiguously identified products are shown in Scheme 2a along with possible reaction pathways shown in Scheme 2b. Table 1 summarizes the different conditions used. The application of automated column chromatography resulted in a residue (referred to as “Rest”; see Table 1), which was tedious to separate and was therefore performed only in selected cases (*vide infra*). Starting with commonly used *p*-toluene sulfonic acid (*p*TsOH),^28,33,34^ we investigated other Brönsted acids, triethylamine (TEA), molecular sieve, and other Brönsted bases, among them different stearates, carbonates, and acetates with mono- and divalent cations (Table 1). Cross-aldol product **11** was found in all conditions as major product in broadly varying yields between 33% and >95%. Along with **11**, its fused analog **12** was also isolated. We hypothesize that this isomerization is promoted by partial hydrolysis of the starting material (Scheme 2b).^70^ Homocoupled benzofuranone **13** was also isolated from almost all conditions. Homocoupling of benzodifuranone is the basis of the synthesis of PBDFO under oxidative conditions.^38^ An alternative pathway to **13** would entail the aldol reaction of **9** with 2,3-benzofurandione, which could form from **9** in situ. Compound **14** is the isomerized product of **13**, the formation of which is believed to be promoted by water.^71^ Finally, homocoupled isatin **15** was frequently observed. Isatin homocoupling has previously been proposed to form under acidic conditions, although details of the mechanism remained unclear.^48^ Here, **15** is formed under basic conditions, suggesting that alternative pathways such as pinacol-type radical couplings^72^ might be operative. Further products contained in the remaining polar fraction “Rest” (Table 2) were generally complex mixtures that we were unable to separate and fully identify by NMR and MS analyses. These are believed to consist of polar components such as unreacted *N*-methylisatin, higher molecular weight adducts, hydrolyzed structures, and other defects of unknown nature. From this polar fraction, only in some cases other defects, such as **15**, **16**, and **17**, could be isolated via preparative thin layer chromatography (TLC) and fully characterized by NMR spectroscopy, MS, and X-ray crystallography (See Supporting Information). X-ray crystallographic characterization of **15** has already been reported elsewhere.^73^ Somewhat independent of the composition of the polar fraction, the lower its overall mass, the more efficient the catalytic system and the higher the yield of the other fractions were.

**Table 2:** Reaction Conditions for Polymerization Reactions

conditions	catalyst	conc.[mM]	time [h]	yield %^a^	*M*_n, SEC_ [kg/mol]^b^	*M*_w, SEC_ [kg/mol]^b^	LUMO [eV]^c^	*E*_U_ [meV]
A	pTsOH	0.01	72	55	11.0	29.7	–4.09	73.52
E	TEA	0.01	72	33	9.9	28.1	–4.29	118.69
G^d^	Mg(st)_2_	0.01	72	45	17.6	36.8	–4.22	101.31
I	Zn(st)_2_+ 4Å MS spatially separated	0.005	28	97	18.1	39.3	–4.22	48.80

a Isolated yield after Soxhlet extraction.

b From HT-SEC in TCB at 160 °C.

c From cyclic voltammetry of drop-casted *o*-DCB solutions into ITO glass substrates.

d Low yield due to incomplete extraction from the Soxhlet thimble.

Conditions A-C used pTsOH as a catalyst. The outcome of using conditions A, which are standard conditions^33,34,40^ is characterized by a good yield (∼82%) of cross-aldol product **11** and small amounts of undesired **12–15**. Conditions B were the same as conditions A but without an argon atmosphere (open flask), under the assumption that an external oxidant, such as oxygen, could inhibit the formation of certain defects, such as **15**, by blocking carbonyl reduction of the isatin or promoting the formation of **13** via the radical pathway. However, according to the similarly low yields of **12**–**15** and the similarly good yield of **11**, this assumption could not be confirmed. For this reason, all remaining reactions were conducted open flask. Under conditions C, light was excluded from the system. A slight decrease in the yield of **11** from ∼80% under conditions A and B to ∼73% under conditions C was observed along with a slightly larger polar fraction. This may indicate that photochemical pathways are additionally operative in this case.^74^ However, we note that ambient conditions, including both light and air, did not negatively affect the outcome of the model reaction. Next, hydrochloric acid was tested (conditions D), giving the lowest yield of **11** (24%) among all conditions and the largest mass of the polar fraction, likely resulting from the hydrolysis of the heterocycles to give open-ring forms.

Fundamental differences were observed when switching from acid to base catalysis. In acid catalysis, an enol intermediate formed via protonation of the ketone serves as nucleophile, which is in equilibrium with the inactive keto form. In base-catalyzed aldol condensations, an ionic enolate serves as the nucleophile. We tested TEA (condition E) as a good base with low nucleophilicity, which had already been used for the polycondensation of benzodifuranone and bisisatin monomers.^75^ Using TEA, the yield of **11** was only 60%, and the amount of the polar fraction increased compared to condition B. From this polar fraction, products **16** and **17** were isolated by preparative TLC and characterized by NMR, MALDI-ToF, and single crystal X-ray diffraction (Figure 1). Product **16** forms via the addition of triethylamine to lactone **11′**, which is the *cis* isomer of **11** (Scheme 2c). Thus, the geometry of **16** with the two carbonyls in *cis* configuration suggested that **11′** possibly formed also in other conditions. We suggest that the carbonyl repulsion in **11′** generates torsion and renders the lactone more reactive toward nucleophilic attacks, gaining stability from ring opening. One possible reason as to why pure **11′** could not be isolated from any other conditions is its suspected high polarity and instability toward hydrolysis. Thus, hydrolyzed *cis* defects may be hidden in their open forms in the remaining polar fraction. Such *cis* defects in aldol polymers are strong kinks that disturb chain packing; in fact, they have already been identified by high-resolution STM imaging.^48^ **17** was a further side product from conditions E and apparently contains an ethylene fragment originating from TEA. The structure of **17** was unequivocally confirmed by NMR, MALDI-ToF MS and single crystal X-ray crystallography (Figure 1).

**Figure 1:**
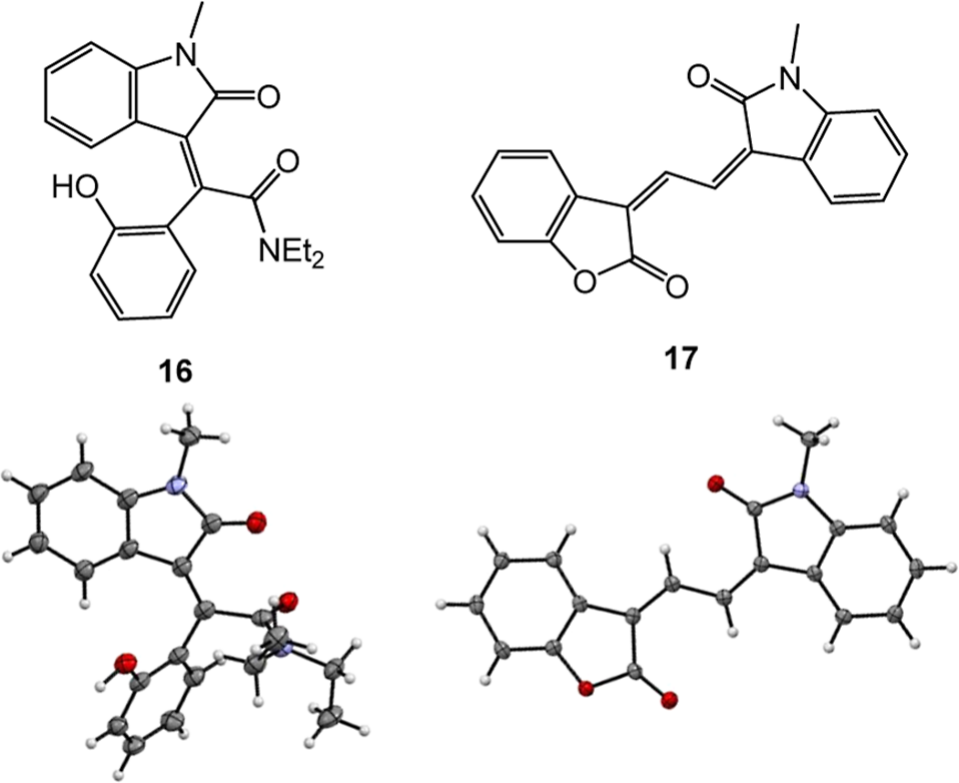
Single crystal X-ray structures of defects **16** and **17** that form when the aldol reaction is catalyzed by triethylamine.

To generate the enolate with fewer side reactions, we next tested weaker bases. In conditions F and G, zinc and magnesium stearate, respectively, were used, both of which are well miscible with toluene. Zn­(st)_2_ as a catalyst showed an improvement in yield to **11** (∼86%), but also a slightly larger yield of the side product **15**. The 2-coumarone homocoupling products **13** and **14** were obtained in slightly lower yields. Notably, a significant reduction in the remaining polar fraction was observed for this catalyst. Using Mg­(st)_2_ did not further improve the outcome but did promote the formation of byproducts. More specifically, a large yield of **15** of ∼11% was found. Possibly, the large yield of **15** may be caused by a pinacol-type homocoupling between two isatins catalyzed by the divalent and oxophilic Mg^2+^ acting as a joint coordination site, a mechanism known for the coupling of aldehydes and ketones.^72^ On the basis of the hard–soft-acid–base theory (HSAB),^76^ Zn^2+^ is softer compared to Mg^2+^ and hence, stabilization of the enolate might be less efficient. As a result, the presence of Zn^2+^ reduced homocoupling of **9** and increased the reactivity of the enolate, which finally led to a reduction of the mass of the polar fraction compared to conditions G (Mg^2+^).

Settling on Zn­(st)_2_ as a catalyst, we tested 4Å mol sieve (MS) as an additive, since the formation of the fused defects **12** and **14** is thought to involve water (conditions H, Scheme 2b). However, the yield of **11** was slightly lower compared to conditions F where Zn­(st)_2_ alone was used, and the yields of all defects increased except for **14**, which was not found. Apart from the absence of **14** in conditions G, additional MS in the reaction mixture did not improve the overall outcome. Possibly, side reactions on the surface or within the pores of the zeolite-based molecular sieves were involved. Nevertheless, as water is a byproduct of the reaction that is furthermore involved in the formation of defects, we designed a reaction setup in which the continuous removal of water was achieved by avoiding physical contact between the molecular sieves and the reactants. To this end, a round-bottom flask was equipped with a dropping funnel charged with activated 4Å MS, topped with a Vigreux column, and closed with a drying tube (see Supporting Information Figure S2). This reaction setup yielded an almost quantitative yield of **11** with minimal byproducts (Conditions I), confirming that water removal is essential. An exact quantification of the yield was not possible, as trace amounts of compounds were found along with cross-aldol product **11** in very polar fractions. However, based on the mass of pure fractions, the isolated yield of **11** was >95%, which, with the polar fraction being largely absent, we considered as a conservative lower bound. Using MS alone in a control experiment (Conditions J), a mediocre yield of 47% was obtained along with a large polar fraction of unidentified products. The robustness of the reaction to deliver **11** in the absence of any catalyst was also checked (Conditions K), furnishing a low yield of **11** of 33% and a similarly large polar fraction compared to conditions J. Finally, the counterion of the enolate was further varied. Conditions L-O test the effect of different acetates (Na, K, and Zn) as well as lithium carbonate. In general, a high level of defects was formed for all these conditions. Sodium acetate yielded the largest amounts of defects, with **12** and **15** being obtained in ∼20% and ∼16% yield, respectively. Zinc acetate, having the softest counterion, furnished the largest yield of **11** and the lowest mass of remaining polar fraction.

From the results presented in Table 1, we consider a mechanistic scenario for the Zn­(st)_2_-catalyzed reaction possible, in which both the Zn^2+^ and the stearate anion play key roles. Lewis acids have been proposed to promote soft enolization by coordinating and activating the carbonyl species.^77^ In this context, Zn^2+^ appears particularly suitable, being sufficiently electrophilic to activate the carbonyl while remaining small enough to retain the substrates within the coordination sphere. Scheme 3 depicts a proposed catalytic cycle using Zn­(st)_2_. In the first step, Zn­(st)_2_ **I** activates benzofuranone via carbonyl substitution. The ligand exchange alters the coordination mode of the carboxylate from κ^2^-*O*,*O*′ to κ^1^-*O*. In the resulting intermediate **II**, the benzofuranone is intramolecularly deprotonated, releasing stearic acid and generating the Zinc enolate **III**. In **III**, the lower coordination of zinc allows the carbonyl oxygen of isatin as a new ligand, resulting in **IV**. Here, nucleophilic attack of the enolate at carbonyl carbon of the isatin generates the new C–C bond. The addition product **V** is protonated and undergoes ligand exchange with stearate to yield the lactol **VI**, which is possibly stabilized by an intramolecular H-bond. Elimination of water yields **11**. Notably, the Zn metallic center does not undergo redox transformations and thus acts solely as an auxiliary platform to facilitate the coupling reaction.

**Scheme 3:**
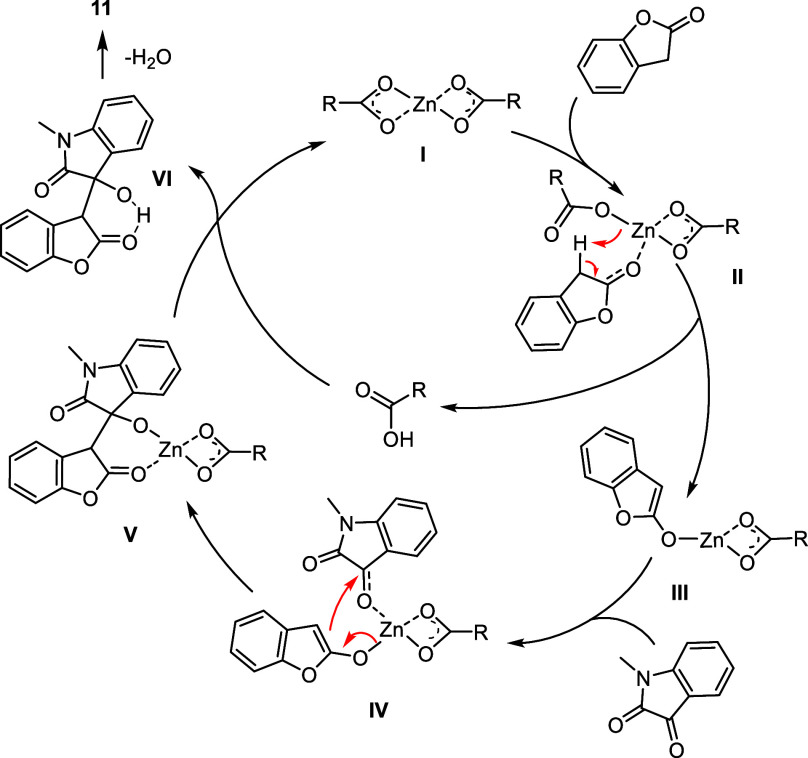
Tentative Catalytic Cycle for the Formation of Cross-Aldol Product **11** Using Zn­(st)_2_ as Catalyst

### Synthesis of PBDFTzTz by Aldol Polycondensations

The outcomes of the model reactions were used to guide the polymerization conditions, and selected conditions were applied to the aldol polycondensation of monomer **4** with benzodifuranone to yield **PBDFTzTz** (Scheme 4a).

**Scheme 4:**
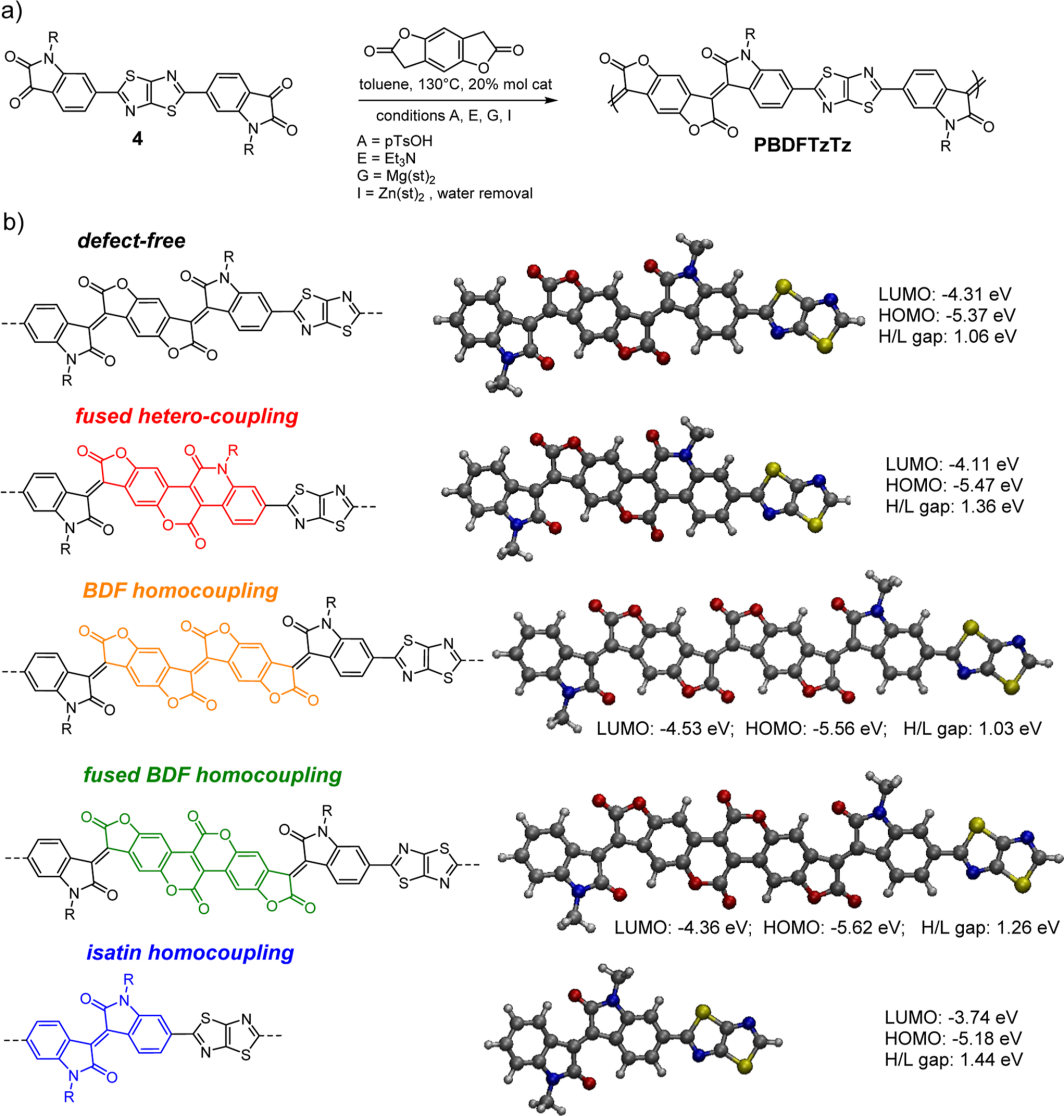
(a) Synthesis of PBDFTzTz via Selected Conditions Shown in Table 2; (b) Possible Defect Structures Embedded in PBDFTzTz Together with the Calculated HOMO and LUMO Energy Levels of Isolated Structures from DFT/PBE/DZVP Calculations

Table 2 summarizes these conditions, along with the molecular data for the resulting **PBDFTzTz** samples. The reaction parameters were selected based on literature protocols^33,39^ and the conditions of Table 1. Specifically, conditions A, E, G, and I were used. It is noteworthy that the use of pTsOH is common in the literature for this type of polycondensation involving benzodifuranone motifs.^28,33,39^ The prevalent use of acid catalysis can be rationalized by the base-labile nature of the aromatic lactones; however, appropriate selection of base may enable polymerization while avoiding hydrolysis. Molar masses were determined via high temperature size exclusion chromatography (HT-SEC) in 1,2,4-trichlorobenzene at 160 °C (Figures S3 and S4).

Commonly used conditions A gave a low yield of **BDFTzTz** of 55% and a low number-average molar mass *M*_n,SEC_ of 11 kg/mol, even after a prolonged reaction time of 72 h. Using TEA as a catalyst (condition E), the polymer yield and molar mass decreased to 33% and 10 kg/mol, respectively, at the same reaction time of 72 h. The decrease in yield and molar mass correlates with the lower yields and larger polar fraction observed in the corresponding model reactions. The same conditions were used with magnesium stearate as a catalyst (conditions G). Here, the yield improved slightly but remained below 50%, while the molar mass increased to 17.6 kg/mol. Given that an improvement in the yield of model product **11** was observed for zinc stearate under continuous dehydrating conditions, conditions I were also tested for the aldol polycondensation of **4** and BDF. Here, the polycondensation was much faster, and the monomer concentration had to be lowered to 0.005 M to control the reaction. Moreover, the reaction time had to be adjusted to avoid precipitation. Longer reaction times compromised the processability of the final polymer due to very high molar masses, and therefore, the reaction time was limited to 28 h. For these lower concentrations and shorter reaction times, a molar mass of 18.1 kg/mol and an almost quantitative isolated yield of 97% after purification by Soxhlet extraction was obtained. All these observations confirm that Zn­(st)_2_ outperforms all other catalytic conditions tested herein, both in the model reactions and in the aldol polycondensations.

To understand the evolution of molar mass under conditions I, **PBDFTzTz** was synthesized on a larger scale, and aliquots were collected at different times. As HT SEC is costly and tedious, an attempt was made to correlate chain length with optical absorption. This would allow for simpler, faster monitoring of the molar mass of **PBDFTzTz** and possibly similar aldol polymers as well. Aliquots were taken from the crude reaction mixture and diluted directly in *ortho*-dichlorobenzene (*o*-DCB) to a concentration of 0.02 mg/mL with respect to the initial monomer concentration. The increase in absorption coefficient with reaction time is consistent with an increasing conjugation length as the polymer chains grow (Figure 2a,b). In addition, the relative intensities of the 0–0 and 0–1 vibronic bands clearly changed with reaction time. Comparing the molar masses from HT-SEC with the 0–0/0–1 ratio showed a monotonic dependence and confirmed that monitoring the aldol polycondensation using steady-state absorption is reasonable and simplifies reaction control (Figure 2c). After 28 h, the absorbance reaches a plateau and subsequently decreases after 48 h, not due to side reactions or depolymerization, but rather to the formation of insoluble aggregates. Data for aliquots with reaction times >28 h were obtained by boiling the samples in *o*-DCB, followed by filtering; however, concentrations could no longer be accurately determined.

**Figure 2:**
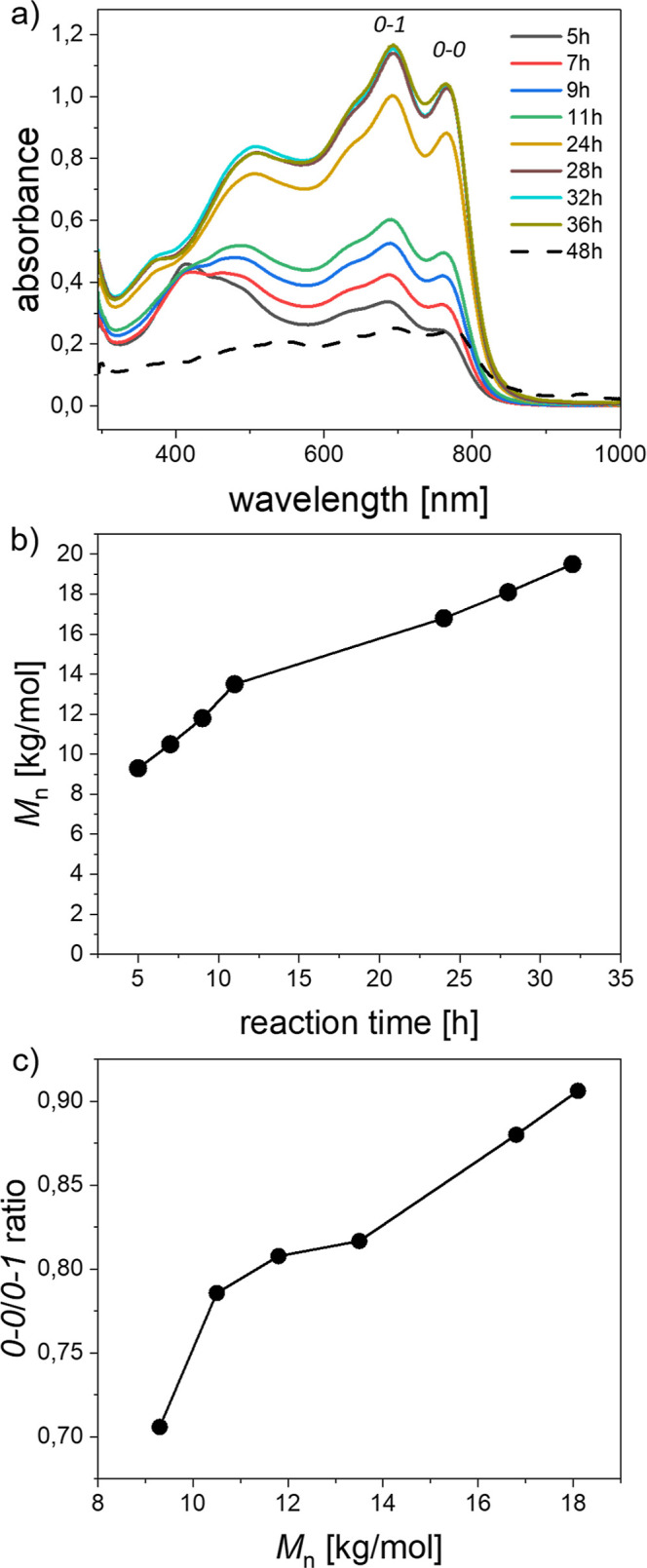
(a) UV–vis spectra of aliquots taken during the synthesis of **PBDFTzTz** under conditions I. Known volumes of crude mixture were taken and diluted with *o*-DCB to reach a concentration of 0.02 mg/mL with respect to the initial monomer concentration. (b) Molar masses from SEC in 1,2,4-trichlorobenzene at 160 °C. (c) Vibronic band ratios 0–0/0–1 versus reaction time.

### Defect Analyses of PBDFTzTz

With the help of the model reactions and the obtained model compounds **11**–**15**, as well as a theoretical assessment of defect models via DFT (gas phase), we attempted to uncover the spectroscopic signatures of possibly built-in defects in **PBDFTzTz** (Scheme 4b). We first invoked steady-state UV–vis spectroscopy of isolated compounds **11**–**15** (Figure S5). While the fused compounds **12** and **14** present a similar signature with maxima near 400 nm and onset wavelengths near 450 nm, compounds **11**, **13**, and **15** featured bathochromically shifted maxima and onset wavelengths, indicating smaller optical band gaps. This indicates that fusing units always widens the band gap. Cyclic voltammetry (CV) analysis of the model compounds also reveals significant information about the variance of the energetic levels of the different defects. Assessment of the HOMO–LUMO levels can be extracted (Figure S6), and their values align well with their optical band gaps. Upon moving from 5-membered rings to their fused isomers, the LUMO level is less negative. This can indeed translate into more fused-defect-rich polymer backbones, with a detrimental impact on their LUMO levels. The HOMO–LUMO gaps of model compounds **11**–**15** were also calculated in the gas-phase using DFT (Figure S7). The obtained values confirm the trends and differences in optical absorption and CV (Figures S6 and S8). For the fused defects **12** and **14**, similar HOMO–LUMO gaps of 2.32 and 2.27 eV, respectively, were obtained. Significantly smaller band gaps were obtained from **11**, **13** and **15**, which had 1.62, 1.72, and 1.62 eV, respectively. Note that the sequence and relative differences of these HOMO–LUMO gaps agree with the absorption onsets presented in Figure S7. In contrast to the model compounds **11**–**14**, the BDF units in **PBDFTzTz** are symmetric and include a second furanone unit, which extends the π-electronic system and significantly affects the electronic levels. To assess these effects, we calculated the energetic levels for the corresponding extended models, referred to as **18**–**21** (Figure S7). Due to the extended π-system, the gap between HOMO and LUMO levels reduces by ∼0.2 and 0.3 eV for models **18** and **19**, and by ∼0.5 eV for compounds **20** and **21**. Upon comparing **11** with **13** and **18** with **20**, it becomes clear that BDF homocoupling reduces the band gap compared to the alternating structure. According to these results, homocoupled BDF units exhibit the smallest gap among all defect structures probed. This observation persists even in more extended theoretical models of defects possibly present in **PBDFTzTz**, which now include the TzTz motif.

Scheme 4b compiles the chemical structures of various homocoupled motifs together with their geometry-optimized gas phase structures and corresponding HOMO/LUMO values.

Absorption profiles of **PBDFTzTz** were taken in *o*-DCB at a concentration of 0.02 mg/mL (Figures 3a and S9). Significant differences were observed when comparing the spectra of the samples prepared under conditions A, E, G, and I. The spectra of **PBDFTzTz** made by conditions A and E showed a higher intensity around 400 nm, as well as a relatively strong tailing at wavelengths >800 nm. We assign the tailing at >800 nm to nonfused BDF homocoupling, as this defect is the only one characterized by a lower band gap than the regular polymer and all other structural defects. We also found an increased amount of **13** in the model reaction under condition E (Table 1). In fact, as **14** is produced from **13**, the sum of **13** and **14** is the relevant quantity that could be considered as an upper limit. However, whether fusion occurs during polymerization or not is something we cannot reliably comment on. We also excluded the possibility that the tailing in the UV–vis spectrum of **PBDFTzTz** made by conditions A and E arose from aggregation. Upon heating the solution, the vibronic 0–0 and 0–1 bands at 700 and 770 nm changed in intensity, but the tail at ∼800 nm did not. This is another indication that the tailing is caused by molecularly built-in defects rather than aggregates.

**Figure 3:**
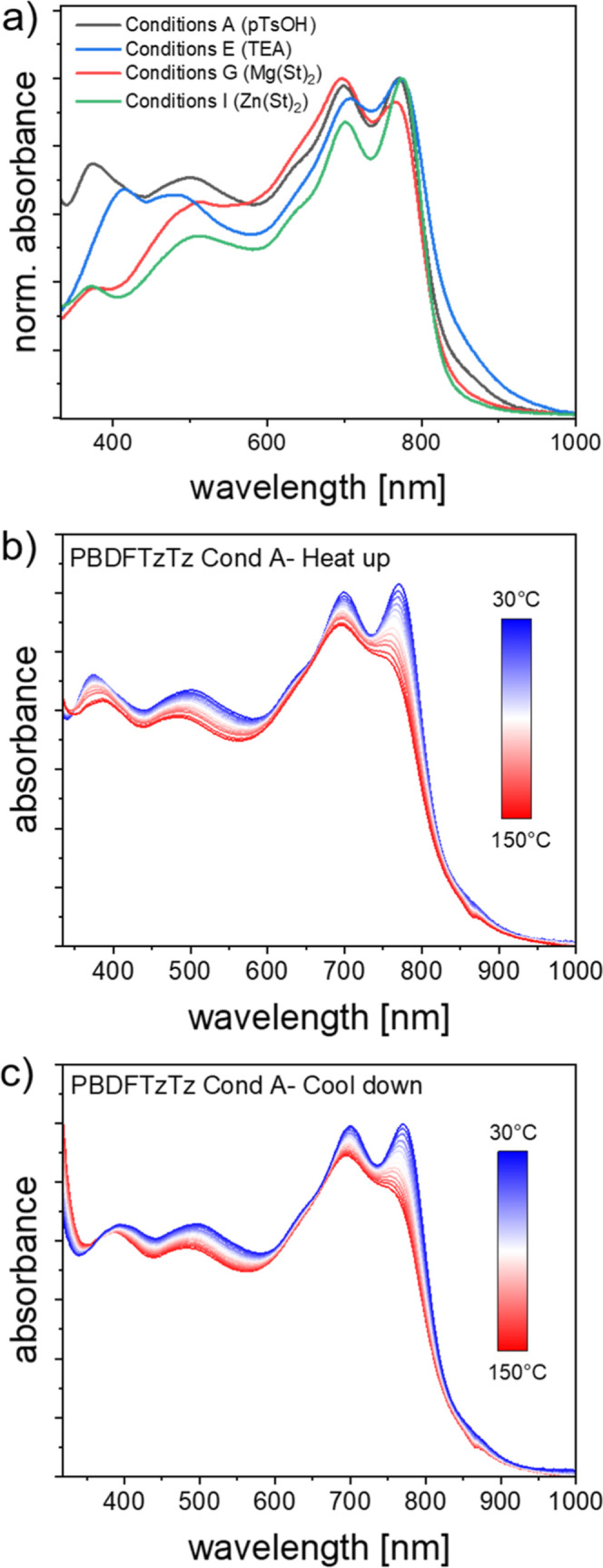
(a) UV–vis spectra of **PBDFTzTz** synthesized under different conditions in *o*-DCB at 25 °C. (b) Heating curve in variable-temperature UV–vis spectra in *o*-DCB of **PBDFTzTz** made with conditions A. (c) Cooling curve of the same sample as shown in (b).

The varying intensities in the absorption spectra in Figure 3a of the four samples between 350 and 550 nm are likely caused by various defects. On the basis of the large oscillator strength of homocoupled models **13** and **14** as well as the relatively large band gaps of the fused defect structures (Figure S7) and the increased amount of defects in conditions A, E and G (Table 1), we ascribed the higher intensity between 350 and 550 nm to the presence of fused and nonfused BDF and isatin homocoupled sequences. A more precise assignment of UV–vis signatures with distinct defects is not possible.

Besides these changes, the two vibronic 0–1 and 0–0 bands at 700 and 770 nm, respectively, are a sign of the extent of conjugation along the main chain (c.f. Figure 2c). Thus, their relative intensities are not only caused by chain length but are additionally influenced by molecularly built-in defects that alter the extent of conjugation. Therefore, the similar molar masses of **PBDFTzTz** made by conditions A and E allow for a qualitative statement on defect density, with the lower 0–0/0–1 ratio of the TEA catalyzed sample (conditions E) indicating a larger amount of defects. The same can be done for **PBDFTzTz** made by conditions G and I, for which the molar masses are very similar. The 0–0/0–1 ratio of **PBDFTzTz** made by Mg­(st)_2_ features is significantly smaller, indicating a much larger defect density compared to **PBDFTzTz** made under conditions I. Again, this correlates well with the results from Table 1, where conditions G caused significant isatin homocoupling. Instead, conditions I were those that led to the least side reactions. As a result, we anticipate that the absorption spectrum of **PBDFTzTz** prepared under conditions I reflects a backbone structure with minimal chemical defects.

To obtain more quantitative information on the presence of defects in **PBDFTzTz** backbones, we performed photothermal deflection spectroscopy (PDS) on thin films of the four polymers. We extracted the Urbach energies *E*_U_ from the low-energy onset of absorption. The *E*_U_ is obtained from the linear slope of the onset of absorption and is a measure of energetic disorder in a thin film, which can be influenced by various factors, including packing and morphology. If, however, molecular defects are present, the energetic disorder of both single chains and aggregates/multiple chains is expected to be higher as well. We therefore anticipated that this method would be a useful addition for probing molecular defects in aldol polymers. From Figure 4, a clear trend of decreasing *E*_U_ values was obtained for conditions E (TEA), G (Mg­(st)_2_), A (pTsOH), and I (Zn­(st)_2_ with water removal). Again, the best catalytic conditions produced the least defective material, with **PBDFTzTz** synthesized under conditions I, yielding the smallest *E*_U_. Moreover, a decrease in *E*_U_ correlated with an increase in yield to **11** in the model reactions (Table 2). Thus, PDS is an excellent method to qualitatively probe molecular defects in **PBDFTzTz.**

**Figure 4:**
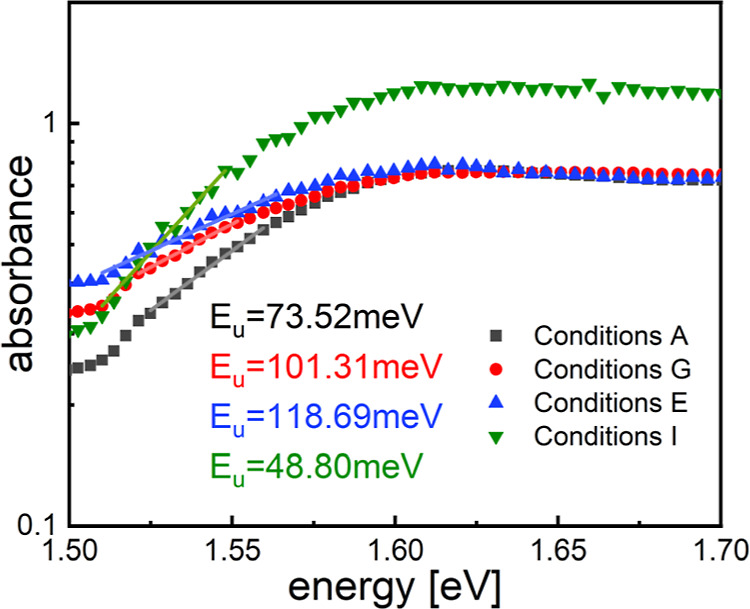
Absorbance of the four **PBDFTzTz** samples made under conditions A, E, G and I measured by photothermal deflection spectroscopy (PDS). From the slopes at the low energy onset (straight lines) the Urbach energies *E*_U_ are extracted.

Although both UV–vis and PDS measurements consistently showed that structural defects are associated with changes in the absorption spectra and that conditions I yielded the least defective polymers, these techniques rely on indirect observables and thus provide only limited structural insight into the nature of the defects. In particular, the chemical identity of the defects cannot be explicitly resolved, nor can their frequency be reliably determined. To address these limitations, a more direct characterization method is required. ESD-STM enables direct visualization of defect structures along individual polymer chains, allowing precise identification of distinct defect types and quantification of their relative and absolute concentrations.^23,46,48,78,79^

Here, ESD-STM analysis was performed on **PBDFTzTz** synthesized using conditions I. For solubility reasons, a sample with a reaction time of 5 h was used. Figure 5a shows a representative STM image of submonolayer coverage, in which defect-free chains were identified by fitting scaled molecular models to the observed features (see inset). The method used to determine the exact molecular sequence of the polymer backbone is described in the Supporting Information. Consistent with recent STM studies on aldol-derived conjugated polymers,^48^ two main types of defects were identified: homocouplings and kink defects. Homocouplings are readily recognized by changes in the periodicity of side chain attachment points along the polymer backbone, whereas kink defects appear as pronounced deviations from the otherwise overall linear chain conformation of **PBDFTzTz**. Homocouplings involving both isatin and BDF units were identified (Figure 5b,c, respectively). In both cases, however, it was not possible to distinguish between simple and fused homocouplings, as the associated differences in feature dimensions fall below the resolution limit of the measurement. Kink defects were also observed (Figure 5d) and are attributed to a *cis* coupling between BDF and isatin. Although the resolution does not allow a strict distinction between structures **11′** and **16** (see Scheme 2b), both of which produce similar backbone kinks, we assign this defect predominantly to **11′**, as TEA was not used in condition I.

**Figure 5:**
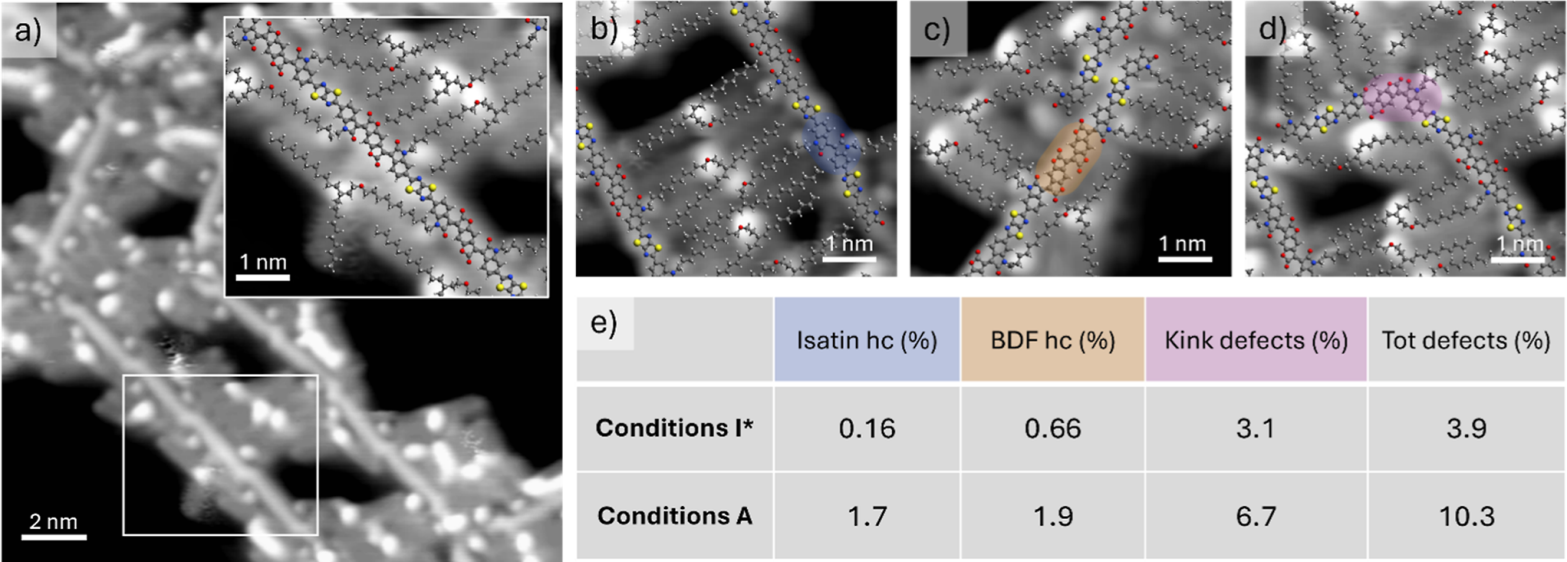
ESD-STM analysis of **PBDFTzTz**. (a) STM image of **PBDFTzTz** synthesized under conditions I* and deposited by ESD onto a Au (111) surface (black background). The inset shows a magnified section with superimposed molecular models, highlighting a defect-free sequence. (b) Isatin homocoupling, (c) BDF homocoupling, and (d) kink defect. (e) Defect frequencies obtained from analyzing a large number of STM images of **PBDFTzTz** synthesized under conditions A and I. (* after a reaction time of 5 h).

The same ESD-STM analysis was performed on **PBDFTzTz** synthesized under conditions A, revealing the same defect types, albeit with different frequencies. The absolute and relative defect distributions for both synthetic conditions are summarized in Figure 5e. Notably, the total defect density in **PBDFTzTz** prepared under conditions I is below 5%, i.e., less than half of that observed for samples synthesized under conditions A (≈10%). This quantitatively confirms the trends inferred from model reactions and from UV–vis and PDS measurements of the polymers, demonstrating that the material obtained under conditions I is significantly less defective. In comparison with previous ESD-STM studies of *n*-type copolymers prepared by aldol condensation under acidic conditions,^48^ similar frequencies of kink defects were observed, whereas homocoupling defects were significantly more prevalent in those systems, occurring at least four times more frequently, and in some cases up to 20× more frequently.

A comparison between the defect distributions obtained by ESD-STM and those derived from NMR analysis of the corresponding model reactions (Table S1) reveals similar overall trends, such as a higher prevalence of BDF homocouplings relative to isatin homocouplings. However, notable differences are also observed, most prominently in the case of kink defects, which are abundant in the polymer samples but detected only in trace amounts in the model systems. These discrepancies can be rationalized by differences in reaction equilibria between small-molecule model systems and polymerizations,^48^ likely influenced by the lower solubility of the growing polymer chains. This effect is particularly relevant for kink defects: while *cis*-couplings may initially form in both systems due to random encounters, they can readily revert and reorganize into the energetically favored *trans* configuration in small molecules owing to the reversibility of the aldol-mediated coupling and their high mobility. In contrast, although the same mechanisms should allow for reversibility in aldol polymers as well, such *cis*–*trans* isomerizations are hindered, as they require rotation of extended chain segments. This may increase the likelihood that *cis* couplings persist.

These findings highlight that, although small-molecule analogs provide valuable insight into defects and their possible reaction pathways, they do not fully capture the defect landscape in the corresponding polymers. Direct characterization of polymer chains is therefore essential for a quantitative understanding of defect formation, which is enabled here by ESD-STM.

Finally, the analysis of the STM data reveals that, in both batches of **PBDFTzTz**, the majority of polymer chains terminate with isatin units (∼90%, see Table S1 and Figures S10–S13). Symmetric end group termination can result from off-stoichiometries, distinct mechanistic features, or different reactivities.

Since an unintentional off-stoichiometry can be excluded and symmetric isatin termination is found for both Zn­(st)_2_ and pTsOH-catalyzed conditions, we propose that the underlying reason is instead linked to an increased reactivity of the second furanone in monofunctionalized BDF that is already linked to one isatin compared to pristine BDF monomer.

In this way, bis-isatin-terminated oligomers would be made first, followed by further coupling with remaining pristine BDF units at later stages of polycondensation. Such a situation has implications for the evolution of molar mass as a function of stoichiometry, as well as opens up the possibility to make block copolymers.

## Conclusions

In this work, we have explored the intricacies of aldol polycondensations between benzodifuranone and a bis-isatin monomer. A new, linear, and coplanar monomer, 6,6′-bisisatinthiazolo­[5,4-*d*]­thiazole, was synthesized using a metal-free, open-flask methodology and copolymerized with benzodifuranone under broadly varying catalytic aldol conditions. These conditions were investigated using model reactions of monofunctional substrates. Product distributions were separated, isolated, and quantified. Several defect structures were identified, including homocouplings, fused isomers of homocouplings, *cis* couplings, and more unusual, unexpected defects. The use of zinc stearate in combination with water removal afforded the highest yield of pure cross aldol product and the least amount of defects. Therefore, zinc stearate was identified as a superior catalyst, also in comparison to established aldol conditions involving *p*-toluene sulfonic acid as catalyst. Translation of these different catalytic conditions into aldol polycondensations of benzodifuranone and the bisisatin monomer enabled the synthesis of *n*-type conjugated polymers with varying defect densities, whose optical spectroscopic signatures were compared. While a correlation of the optical signatures of the polymers with defects seen in model reactions was possible to some extent, proof or quantitative information on a certain defect in the chain could not be gained. In contrast, ESD-STM enabled direct imaging of individual polymer chains and the determination of their backbone structure and sequence, allowing identification and quantification of defect structures and providing direct access to the defect landscape of the full polymers. The superior catalytic conditions using Zn­(st)_2_ reduced defect levels approximately by 50% compared to polymers prepared under established aldol polycondensation conditions. ESD-STM also revealed that a majority of chains were terminated by the isatin units. This observation is attributed to the preferential reactivity of asymmetric BDF functionalities over pristine BDF monomers and opens up the possibility for the preparation of block copolymers. This work established a new *n*-type copolymer with low-lying LUMO and identified catalytic conditions that enable access to aldol polymers with markedly reduced defect densities, providing a general strategy for the development of defect-minimized aldol-derived conjugated polymers.

## Supplementary Material





## Notes

Data DFT (ZIP)
Supporting Information Experimental details, synthesis and analysis of the monomers and polymers are described in the Supporting Information (PDF)

## References

[1] Cheng Y.-J., Yang S.-H., Hsu C.-S. (2009). Synthesis of Conjugated Polymers
for Organic Solar Cell Applications. Chem. Rev..

[2] Chen Z., Ding X., Wang J., Guo X., Shao S., Feng K. (2025). π-Conjugated Polymers for High-Performance
Organic Electrochemical
Transistors: Molecular Design Strategies, Applications and Perspectives. Angew. Chem., Int. Ed..

[3] Inal S., Rivnay J., Suiu A.-O., Malliaras G. G., McCulloch I. (2018). Conjugated Polymers in Bioelectronics. Acc. Chem. Res..

[4] Carsten B., He F., Son H. J., Xu T., Yu L. (2011). Stille Polycondensation
for Synthesis of Functional Materials. Chem.
Rev..

[5] Sakamoto J., Rehahn M., Wegner G., Schlüter A. D. (2009). Suzuki
Polycondensation: Polyarylenes à La Carte. Macromol. Rapid Commun..

[6] Cheng S., Zhao R., Seferos D. S. (2021). Precision Synthesis of Conjugated
Polymers Using the Kumada Methodology. Acc.
Chem. Res..

[7] Kimbrough R. D. (1976). Toxicity
and Health Effects of Selected Organotin Compounds: A Review. Environ. Health Perspect..

[8] Kiriy A., Senkovskyy V., Sommer M. (2011). Kumada Catalyst-Transfer Polycondensation:
Mechanism, Opportunities, and Challenges. Macromol.
Rapid Commun..

[9] Krishna, A. ; Lunchev, A. V. ; Grimsdale, A. C. Suzuki Polycondensation. In Synthetic Methods for Conjugated Polymers and Carbon Materials; John Wiley & Sons, Ltd, 2017; pp 59–95.

[10] Kowalski S., Allard S., Zilberberg K., Riedl T., Scherf U. (2013). Direct Arylation
Polycondensation as Simplified Alternative for the Synthesis of Conjugated
(Co)­Polymers. Prog. Polym. Sci..

[11] Wang Q., Takita R., Kikuzaki Y., Ozawa F. (2010). Palladium-Catalyzed
Dehydrohalogenative Polycondensation of 2-Bromo-3-Hexylthiophene:
An Efficient Approach to Head-to-Tail Poly­(3-Hexylthiophene). J. Am. Chem. Soc..

[12] Matsidik R., Komber H., Luzio A., Caironi M., Sommer M. (2015). Defect-Free
Naphthalene Diimide Bithiophene Copolymers with Controlled Molar Mass
and High Performance via Direct Arylation Polycondensation. J. Am. Chem. Soc..

[13] Pouliot J.-R., Grenier F., Blaskovits J. T., Beaupré S., Leclerc M. (2016). Direct (Hetero)­Arylation Polymerization: Simplicity
for Conjugated Polymer Synthesis. Chem. Rev..

[14] Scott C. N., Bisen M. D., Stemer D. M., McKinnon S., Luscombe C. K. (2017). Direct
Arylation Polycondensation of 2,5-Dithienylsilole with a Series of
Difluorobenzodiimine-Based Electron Acceptors. Macromolecules.

[15] Kimpel J., Kim Y., Schomaker H., Hinojosa D. R., Asatryan J., Martín J., Kroon R., Sommer M., Müller C. (2025). Open-Flask,
Ambient Temperature Direct Arylation Synthesis of Mixed Ionic-Electronic
Conductors. Sci. Adv..

[16] Castillo G. E., Thompson B. C. (2023). Room Temperature Synthesis of a Well-Defined
Conjugated
Polymer Using Direct Arylation Polymerization (DArP). ACS Macro Lett..

[17] Mayhugh A. L., Luscombe C. K. (2020). Room-Temperature Pd/Ag Direct Arylation
Enabled by
a Radical Pathway. Beilstein J. Org. Chem..

[18] Pankow R. M., Ye L., Thompson B. C. (2018). Sustainable
Synthesis of a Fluorinated Arylene Conjugated
Polymer via Cu-Catalyzed Direct Arylation Polymerization (DArP). ACS Macro Lett..

[19] Ye L., Pankow R. M., Schmitt A., Thompson B. C. (2019). Synthesis of Conjugated
Polymers Using Aryl-Bromides *via* Cu-Catalyzed Direct
Arylation Polymerization (Cu-DArP). Polym. Chem..

[20] Ye L., Schmitt A., Pankow R. M., Thompson B. C. (2020). An Efficient Precatalyst
Approach for the Synthesis of Thiazole-Containing Conjugated Polymers
via Cu-Catalyzed Direct Arylation Polymerization (Cu-DArP). ACS Macro Lett..

[21] Gobalasingham N. S., Thompson B. C. (2018). Direct Arylation Polymerization:
A Guide to Optimal
Conditions for Effective Conjugated Polymers. Prog. Polym. Sci..

[22] Wang Q., Lenjani S. V., Dolynchuk O., Scaccabarozzi A. D., Komber H., Guo Y., Günther F., Gemming S., Magerle R., Caironi M., Sommer M. (2021). Electron Mobility
of Diketopyrrolopyrrole Copolymers Is Robust against Homocoupling
Defects. Chem. Mater..

[23] Moro S., Spencer S. E. F., Lester D. W., Nübling F., Sommer M., Costantini G. (2024). Molecular-Scale
Imaging Enables Direct
Visualization of Molecular Defects and Chain Structure of Conjugated
Polymers. ACS Nano.

[24] Lombeck F., Komber H., Gorelsky S. I., Sommer M. (2014). Identifying
Homocouplings
as Critical Side Reactions in Direct Arylation Polycondensation. ACS Macro Lett..

[25] Aldrich T. J., Dudnik A. S., Eastham N. D., Manley E. F., Chen L. X., Chang R. P. H., Melkonyan F. S., Facchetti A., Marks T. J. (2018). Suppressing Defect Formation Pathways in the Direct
C-H Arylation Polymerization of Photovoltaic Copolymers. Macromolecules.

[26] Chakraborty B., Luscombe C. K. (2023). Cross-Dehydrogenative Coupling Polymerization
via C-H
Activation for the Synthesis of Conjugated Polymers. Angew. Chem..

[27] Griggs S., Marks A., Meli D., Rebetez G., Bardagot O., Paulsen B. D., Chen H., Weaver K., Nugraha M. I., Schafer E. A., Tropp J., Aitchison C. M., Anthopoulos T. D., Banerji N., Rivnay J., McCulloch I. (2022). The Effect
of Residual Palladium on the Performance of Organic Electrochemical
Transistors. Nat. Commun..

[28] Zhang G., Dai Y., Liu Y., Liu J., Lu H., Qiu L., Cho K. (2017). Facile Green Synthesis of Isoindigo-Based
Conjugated Polymers Using
Aldol Polycondensation. Polym. Chem..

[29] Sato S., Lin P.-S., Wu W.-N., Liu C.-L., Higashihara T. (2020). Atom-Economical
Synthesis and Characterization of Poly­(Oxindolidene Thienylene Vinylene)
Based on Aldol Polycondensation Reaction. Catalysts.

[30] Modern Aldol Reactions, 1st ed.; John Wiley & Sons, Ltd, 2004.

[31] Perrin C. L., Chang K.-L. (2016). The Complete Mechanism of an Aldol
Condensation. J. Org. Chem..

[32] Zhu X., Duan J., Chen J., Liu R., Qin Z., Chen H., Yue W. (2024). Aldol Condensation for the Construction
of Organic Functional Materials. Angew. Chem..

[33] Lu Y., Yu Z., Zhang R., Yao Z., You H., Jiang L., Un H., Dong B., Xiong M., Wang J., Pei J. (2019). Rigid Coplanar
Polymers for Stable n-Type Polymer Thermoelectrics. Angew. Chem., Int. Ed..

[34] Chen H., Moser M., Wang S., Jellett C., Thorley K., Harrison G. T., Jiao X., Xiao M., Purushothaman B., Alsufyani M., Bristow H., De Wolf S., Gasparini N., Wadsworth A., McNeill C. R., Sirringhaus H., Fabiano S., McCulloch I. (2021). Acene Ring Size Optimization in Fused
Lactam Polymers Enabling High N-Type Organic Thermoelectric Performance. J. Am. Chem. Soc..

[35] Che Q., Wei X., Zhang W., Luo H., Cui C., Yang S., Chen J., Chen Z., Wang L., Yu G. (2025). High Electron
Mobility in Conjugated Polymers Processed with Nonchlorinated Solvents
for Organic Thin Film Transistors. ACS Appl.
Polym. Mater..

[36] Lei T., Dou J.-H., Cao X.-Y., Wang J.-Y., Pei J. (2013). Electron-Deficient
Poly­(*p* -Phenylene Vinylene) Provides Electron Mobility
over 1 Cm ^2^ V ^–‑1^ s ^–‑1^ under Ambient Conditions. J. Am. Chem. Soc..

[37] Griggs S., Marks A., Bristow H., McCulloch I. (2021). N-Type Organic
Semiconducting Polymers: Stability Limitations, Design Considerations
and Applications. J. Mater. Chem. C.

[38] Tang H., Liang Y., Liu C., Hu Z., Deng Y., Guo H., Yu Z., Song A., Zhao H., Zhao D., Zhang Y., Guo X., Pei J., Ma Y., Cao Y., Huang F. (2022). A Solution-Processed
n-Type Conducting Polymer with
Ultrahigh Conductivity. Nature.

[39] Onwubiko A., Yue W., Jellett C., Xiao M., Chen H.-Y., Ravva M. K., Hanifi D. A., Knall A.-C., Purushothaman B., Nikolka M., Flores J.-C., Salleo A., Bredas J.-L., Sirringhaus H., Hayoz P., McCulloch I. (2018). Fused Electron
Deficient Semiconducting Polymers for Air Stable Electron Transport. Nat. Commun..

[40] Gao R., Guo Y., Xu C., Dong W., Xu Y., Han Y., Deng Y., Geng Y. (2025). n-Type Polymer Thermoelectric Materials
Based on Benzodifurandione and F-Substituted Thiophene Derivatives
Synthesized via Direct Arylation and Aldol Polycondensation. Macromol. Chem. Phys..

[41] Shih Y.-H., Wu G.-L., Chueh P.-H., Chen J.-C., Tsai C.-Y., Wang T.-Y., Yu M.-H., Li Y.-P., Chen W.-C., Chueh C.-C. (2025). The Influence of Interlocking Effects in Conjugated
Polymers Synthesized by Aldol Polycondensation on Field-Effect Transistor
Properties and Morphology. JACS Au.

[42] Ke Z., Abtahi A., Hwang J., Chen K., Chaudhary J., Song I., Perera K., You L., Baustert K. N., Graham K. R., Mei J. (2023). Highly Conductive and
Solution-Processable
n-Doped Transparent Organic Conductor. J. Am.
Chem. Soc..

[43] Liu G., Hsu H.-H., Samal S., Lee W.-J., Ke Z., You L., Savoie B. M., Mei J. (2025). Selenium Dioxide Catalyzed Polymerization
of N-Doped Poly­(Benzodifurandione) (n-PBDF) and Its Derivatives. Angew. Chem..

[44] Lombeck F., Komber H., Fazzi D., Nava D., Kuhlmann J., Stegerer D., Strassel K., Brandt J., de Zerio
Mendaza A. D., Müller C., Thiel W., Caironi M., Friend R., Sommer M. (2016). On the Effect of Prevalent Carbazole
Homocoupling Defects on the Photovoltaic Performance of PCDTBT:PC71BM
Solar Cells. Adv. Energy Mater..

[45] Wang Q., Böckmann S., Günther F., Streiter M., Zerson M., Scaccabarozzi A. D., Tan W. L., Komber H., Deibel C., Magerle R., Gemming S., McNeill C. R., Caironi M., Hansen M. R., Sommer M. (2021). Hydrogen Bonds Control Single-Chain
Conformation, Crystallinity, and Electron Transport in Isoelectronic
Diketopyrrolopyrrole Copolymers. Chem. Mater..

[46] Vanderspikken J., Liu Z., Wu X., Beckers O., Moro S., Quill T. J., Liu Q., Goossens A., Marks A., Weaver K., Hamid M., Goderis B., Nies E., Lemaur V., Beljonne D., Salleo A., Lutsen L., Vandewal K., Van Mele B., Costantini G., Van den Brande N., Maes W. (2023). On the Importance of
Chemical Precision in Organic Electronics: Fullerene Intercalation
in Perfectly Alternating Conjugated Polymers. Adv. Funct. Mater..

[47] Broll S., Nübling F., Luzio A., Lentzas D., Komber H., Caironi M., Sommer M. (2015). Defect Analysis of High Electron
Mobility Diketopyrrolopyrrole Copolymers Made by Direct Arylation
Polycondensation. Macromolecules.

[48] Wu X., Moro S., Marks A., Alsufyani M., Yu Z., Perdigão L. M. A., Chen X., Luci A. M. T., Crockford C., Spencer S. E. F., Fox D. J., Pei J., McCulloch I., Costantini G. (2025). Revealing Polymerisation Defects
and Formation Mechanisms in Aldol Condensation for Conjugated Polymers
via High-Resolution Molecular Imaging. Nat.
Commun..

[49] Streiter M., Beer D., Meier F., Göhler C., Lienert C., Lombeck F., Sommer M., Deibel C. (2019). Homocoupling
Defects in a Conjugated Polymer Limit Exciton Diffusion. Adv. Funct. Mater..

[50] Hendriks K. H., Li W., Heintges G. H. L., van Pruissen G. W. P., Wienk M. M., Janssen R. A. J. (2014). Homocoupling Defects in Diketopyrrolopyrrole-Based
Copolymers and Their Effect on Photovoltaic Performance. J. Am. Chem. Soc..

[51] Shi K., Zhang F., Di C.-A., Yan T.-W., Zou Y., Zhou X., Zhu D., Wang J.-Y., Pei J. (2015). Toward High
Performance *n* -Type Thermoelectric Materials by Rational
Modification of BDPPV Backbones. J. Am. Chem.
Soc..

[52] Jismy B., Durand P., Jacob J. P., Richard F., Boyron O., Heinrich B., Schmaltz B., Lévêque P., Bardagot O., Leclerc N. (2025). Versatile Direct (Hetero)­Arylation
Polymerization of Electro-Deficient Unsubstituted Thiazolo­[5,4-d]­Thiazole:
A Tool to Lower the LUMO Level. Macromol. Rapid
Commun..

[53] Zhang Z., Shi Y., Li H., Rao M., Deng Y., Geng Y. (2026). Aldol Polycondensation
toward Benzodifurandione-Based Conjugated Polymers for n-Type Organic
Electronics. Polymer.

[54] Matsidik R., Giorgio M., Luzio A., Caironi M., Komber H., Sommer M. (2018). A Defect-Free Naphthalene Diimide Bithiazole Copolymer
via Regioselective Direct Arylation Polycondensation. Eur. J. Org. Chem..

[55] Wang S., Sun H., Erdmann T., Wang G., Fazzi D., Lappan U., Puttisong Y., Chen Z., Berggren M., Crispin X., Kiriy A., Voit B., Marks T. J., Fabiano S., Facchetti A. (2018). A Chemically Doped Naphthalenediimide-Bithiazole Polymer
for n-Type Organic Thermoelectrics. Adv. Mater..

[56] Wei N., Hao L., Li D., Bian Z., Song S., He J., Li C., Zhang A., Liu Y., Bo Z. (2024). Conjugated
Polymers
with Thiazolothiazole as the Acceptor Unit for High Performance Organic
Solar Cells. Dyes Pigm..

[57] Su H.-L., Sredojevic D. N., Bronstein H., Marks T. J., Schroeder B. C., Al-Hashimi M. (2017). Bithiazole: An Intriguing Electron-Deficient Building
for Plastic Electronic Applications. Macromol.
Rapid Commun..

[58] Hinojosa D. R., Pataki N. J., Pallini F., Freychet G., Erhardt A., Schuller K., Olthof S., Meerholz K., McNeill C. R., Caironi M., Günther F., Sommer M. (2026). Interplay of Backbone
Conformation, Morphology and Thermoelectric Properties of Benzodifuranone-Isatin
Acceptor Polymers. Adv. Electron. Mater..

[59] Woodward A. N., Kolesar J. M., Hall S. R., Saleh N.-A., Jones D. S., Walter M. G. (2017). Thiazolothiazole
Fluorophores Exhibiting Strong Fluorescence
and Viologen-Like Reversible Electrochromism. J. Am. Chem. Soc..

[60] R.
Hinojosa D., J. Pataki N., Rossi P., Erhardt A., Guchait S., Pallini F., McNeill C., Müller C., Caironi M., Sommer M. (2024). Solubilizing Benzodifuranone-Based
Conjugated Copolymers with Single-Oxygen-Containing Branched Side
Chains. ACS Appl. Polym. Mater..

[61] Heaney H., Ley S. V. (1973). N-Alkylation of
Indole and Pyrroles in Dimethyl Sulfoxide. J.
Chem. Soc., Perkin Trans. 1.

[62] Wang C.-P., Jiang G.-F. (2017). An Efficient Method
Based on Indoles for the Synthesis
of Isatins by Taking Advantage of I2O5 as Oxidant. Tetrahedron Lett..

[63] Rastogi G. K., Deka B., Deb M. L., Baruah P. K. (2022). Iodine-DMSO-Promoted
Oxygenation of Indoles: Synthesis of Isatin and Isoindigo. Asian J. Org. Chem..

[64] Dai Y.-Z., Ai N., Lu Y., Zheng Y.-Q., Dou J.-H., Shi K., Lei T., Wang J.-Y., Pei J. (2016). Embedding Electron-Deficient Nitrogen
Atoms in Polymer Backbone towards High Performance n-Type Polymer
Field-Effect Transistors. Chem. Sci..

[65] Luo J., Zhao Y., Xu X., Zheng J., Liang H. (2017). Cu-Catalyzed
Oxidation of Indoles to Isatins. Tetrahedron
Lett..

[66] Zi Y., Cai Z.-J., Wang S.-Y., Ji S.-J. (2014). Synthesis of Isatins
by I2/TBHP Mediated Oxidation of Indoles. Org.
Lett..

[67] Tatsugi J., Zhiwei T., Amano T., Izawa Y. (2000). A Facile Synthesis
of 1Alkyl7-Azaisatins. Heterocycles.

[68] Zhu X., Duan J., Chen J., Liu R., Qin Z., Chen H., Yue W. (2024). Aldol Condensation for the Construction
of Organic Functional Materials. Angew. Chem.,
Int. Ed..

[69] Wong K. F., Skaf M. S., Yang C.-Y., Rossky P. J., Bagchi B., Hu D., Yu J., Barbara P. F. (2001). Structural and Electronic Characterization
of Chemical and Conformational Defects in Conjugated Polymers. J. Phys. Chem. B.

[70] Kawase Y., Yamaguchi S., Maeda O., Hayashi A., Hayashi I., Tabata K., Kondo M. (1979). The Synthesis of Benzofuroquinolines.
I. Some Benzofuro­[2,3-b]­Quinoline and Benzofuro­[3,2-c]­Quinoline Derivatives. J. Heterocycl. Chem..

[71] Hwang J., Zhao Q., Ahmed M., Yakisan A. C., Espenship M. F., Laskin J., Savoie B. M., Mei J. (2024). Reductive Doping Inhibits
the Formation of Isomerization-Derived Structural Defects in N-Doped
Poly­(Benzodifurandione) (n-PBDF). Angew. Chem.,
Int. Ed..

[72] Wang J.-S., Li J.-T., Lin Z.-P., Li T.-S. (2005). Magnesium-Induced
Pinacol Coupling of Aromatic Aldehydes and Ketones Under Ultrasound
Irradiation. Synth. Commun..

[73] Voronina Yu. K., Krivolapov D. B., Bogdanov A. V., Mironov V. F., Litvinov I. A. (2012). An Unusual
Conformation of 1,1′-Dimethyl-Isoindigo in Crystals. J. Struct. Chem..

[74] Haucke G., Seidel B., Graness A. (1987). The Photochemistry
of Isatin. J. Photochem..

[75] Tian L., Jing J., Tang H., Liang Y., Hu Z., Rafiq M., Huang F., Cao Y. (2021). Aldol Condensation-Polymerized
n-Doped Conjugated Polyelectrolytes for High-Performance Nonfullerene
Polymer Solar Cells. Sol. RRL.

[76] Ho T.-L. (1975). Hard Soft
Acids Bases (HSAB) Principle and Organic Chemistry. Chem. Rev..

[77] Lewis J. D., Van de Vyver S., Román-Leshkov Y. (2015). Acid-Base Pairs in
Lewis Acidic Zeolites Promote Direct Aldol Reactions by Soft Enolization. Angew. Chem..

[78] Warr D. A., Perdigão L. M. A., Pinfold H., Blohm J., Stringer D., Leventis A., Bronstein H., Troisi A., Costantini G. (2018). Sequencing Conjugated Polymers by
Eye. Sci. Adv..

[79] Bynens L., Mantegazza P., Marks A., Park Y., Goossens A., Moro S., Quill T. J., Lecroy G., Cheng C., Magni A., Lutsen L., Vanderspikken J., Spencer S. E. F., Vandewal K., Salleo A., Costantini G., Maes W. (2026). Revealing the Full Potential of Glycolated Mixed Ionic-Electronic
Semiconductors - Symmetric Monomer Polymerization to Boost Electrochemical
Transistor Performance. J. Am. Chem. Soc..

