# Supplementary material for: Synthetic Method–Defect Correlations in Aldol Polycondensation: From Model Reactions to an n‑Type Thiazolothioazole Copolymer
Source: J Am Chem Soc. 2026 Jul 24;148(30):32195–209. doi: 10.1021/jacs.6c07584 (PMC13450419; doi:10.1021/jacs.6c07584)
Supplement: Supplementary file 1 [file ja6c07584_si_002.pdf]

Supporting Information

Synthetic Method-Defect Correlations in Aldol Polycondensation: From Model Reactions to an *n*-Type Thiazolothioazole Copolymer

Diego R. Hinojosa, Florian Günther, Paola Mantegazza, Matus Stredansky, Stefania Moro, Tobias Rüffer, Chunqiu Zheng, Yana Vaynzof, Giovanni Costantini and Michael Sommer

Table of contents

1. General Materials and Methods..3

2. Experimental Procedures..6

3. Additional Figures..16

4. NMR Spectra...25

5. MALDI ToF MS data...49

6. X-ray diffraction..51

7. References...54

1. General Materials and Methods

Chemicals. All chemicals, reagents and solvents were purchased from commercial sources and used as received unless otherwise noted. Solvents used for column chromatography were distilled under reduced pressure before use. The synthesis of the side chains was performed accordingly to previous procedures.[1]

NMR Spectroscopy. NMR spectra were recorded on an Avance NEO 600 FT spectrometer (^{1}H: 600 MHz, ^{13}C: 150.9 MHz). The spectra were referenced to the residual solvent peaks (chloroform: δ (^{1}H) = 7.26 ppm, δ (^{13}C) = 77.16 ppm). Analysis was executed with Mestrelab Mestrenova v11.0.4. Peak assignments were supported by COSY, HSQC and HMBC experiments.

Matrix Assisted Laser Desorption Ionization (MALDI) TOF. The MALDI-TOF spectra were taken using a BRUKER autoflex maX MALDI-TOF instrument in reflectron positive mode. The laser of this instrument is a smartbeam II with a wavelength of 355 nm. The software for measuring and evaluating the spectra is flexControl 3.4 and flexAnalysis 3.4. Samples were prepared on a standard sample plate (Bruker "MTP 384 target plate polish steel BC"). All samples except intermediate 3 were imaged under matrix-free conditions, i.e. only solutions of analyte (dichloromethane ~1 mg mL^{-1}) were spotted onto the sample plate. Intermediate 3 could not be imaged under these conditions, here a matrix double layer method was used.[2] To this end, 1 μl of analyte solution (dichloromethane ~1 mg mL^{-1}) was first placed on the sample plate and dried under air. Then, the matrix solution (1 μl, *trans*-2-[3-(4-*tert*-butylphenyl)-2-methyl-2-propenyliden]malononitrile (DCTB), 20 mg mL^{-1} in DCM) was spotted on top of the sample droplet and dried under air for a couple of minutes.

Size exclusion chromatography (SEC). High temperature SEC of polymers was performed on an Agilent PL-GPC 220 integrated high-temperature GPC/SEC system in 1,2,4-trichlorobenzene at 150 °C using calibration with polystyrene standards.

Cyclic voltammetry (CV). CV measurements were performed with a scan rate of 50 $mV \cdot s^{-1}$ at RT under an argon atmosphere using a PalmSens4 potentiostat, a Ag/Ag+ pseudo-reference electrode, and a platinum wire as counter electrode. Ferrocene was used for calibration, and a value of −4.80 eV for the half-wave potential of the Fc/Fc+ couple was used for determining the HOMO and LUMO energy levels. The error on the HOMO/LUMO energy levels is estimated to be at least ±0.10 eV.[3] For film measurements, an ITO-coated float glass (from Prazisions¨ Glas & Optik GmbH) , sheet resistance ≤7 $\Omega \cdot sq^{-1}$) as working electrode and NBu_4PF_6 (0.1 M) as electrolyte in dry acetonitrile were used. Thin films were prepared by spin coating (30 s @ 1000 rpm, 30 s @ 3000 rpm) polymer solutions (2.5 g/L in *o*-DCB) onto the ITO electrodes.

UV-vis Spectroscopy. All UV-vis spectra were measured in transmission. UV-vis spectra in solution were measured at the indicated temperature on a Cary 60 UV-vis (Agilent Technologies) using 0.02 mg/mL solutions of polymers in *o*-DCB in sealed glass cuvettes.

X-Ray diffraction. The molecular structures of **11** and **15−17** were determined by single crystal X-ray diffraction (SC XRD). Crystals have been obtained by slow evaporation of dichloromethane solutions of the molecules. Data collection was performed at 100 K with a Bruker Venture D8 diffractometer by using Cu Kα radiation (1.54178 Å). The structure was solved using direct methods with SHELXT 2018/2 and refined with SHELXL-2013.[4,5] All non-carbon atoms were refined anisotropically while carbon-bonded hydrogen atoms were placed on calculated positions isotropically. In case of **11** and **16** the asymmetric unit comprises two crystallographic independent molecules of identical connectivity. Furthermore, the position of oxygen-bonded hydrogen atoms of **16** were taken from different Fourier maps and refined freely. Table S1 gives selected crystal data and structural refinement details of **11** and **15−17**.

Photothermal deflection spectroscopy (PDS). The photothermal deflection spectroscopy (PDS) spectra were measured using the procedure detailed in previous works.[6–8] The films were mounted in the signal-enhancing liquid-filled (Fluorinert FC-770) quartz cuvette inside a N_2-filled glovebox. Next, the samples were excited using a tunable, chopped, monochromatic light source (150 W xenon short arc lamp with a Cornerstone monochromator) and probed using a laser beam (635 nm diode laser, Thorlabs) propagating parallel to the surface of the samples. The heat generated through the absorption of light changes the refractive index of the Fluorinert liquid, resulting in the deflection of the laser beam. This deflection was measured using a

position-sensitive detector (Thorlabs, PDP90A) and a lock-in amplifier (Amatec SR7230) and is directly correlated to the absorption of the films.

Electrospray deposition scanning tunneling microscopy (ESD-STM). The two samples of **PBDFTzTz** analyzed by ESD-STM were synthesized under conditions I (but with a reaction time of 5h) and under conditions A. Both polymers were dissolved in chlorobenzene (≈ 0.03 g/L) and diluted with methanol in a 4:1 volume ratio. The solutions were then electrosprayed using a four-stage Molecularspray Ltd. system onto clean Au (111)/mica substrates (Georg Albert PVD, 300 nm thickness) at room temperature. The gold substrates had been pre-treated with cycles of argon ion sputtering followed by annealing at 500°C in ultra-high vacuum (UHV). STM measurements were performed at $\sim 10^{-10}$ mbar and 77 K using a low-temperature STM (CreaTec Fischer & Co. GmbH). For the analysis of the STM images, molecular models of individual monomers were optimized using Avogadro[9] and manually superimposed onto high-resolution STM images (lateral size < 20 nm) using the LMAPper software.[10,11] All possible planar conformations, obtained by rotating each subunit around the single C-C bonds, were considered (referring to the torsional potential in Scheme 1a). The monomers were placed sequentially onto the images to identify the polymeric sequence that best matched the observed STM features, as shown in Figures S63 and S564.

Density functional theory (DFT). Molecular models of the **PBDFTzTz** were examined using density functional theory calculations carried out with the deMon2k software package.[12] The computations employed the generalized-gradient exchange–correlation functional developed by Perdew, Burke, and Ernzerhof (PBE)[13] together with a double-ζ basis set including polarization functions.

2. Experimental Procedures

Failed attempts toward the synthesis of 4. Different approaches were explored within the retrosynthetic analysis of the new bisisatin monomer **4** (**Scheme 1b**). The first and most common synthesis of the TzTz motif flanked with aromatic units reported by Ketcham starts with an aldehyde.[14] First, we attempted to prepare 6-isatin-carbaldehyde **8** *via* lithium-halogen exchange, but obtained decomposed products only when using both ketone-protected and unprotected isatins for details of unsuccessful reactions, see Schemes S1 and S2.

Attempt to prepare 6,6'-bisisatinthiazolo [5,4-d] thiazole *via* electrophilic ring cyclization. In a 100 mL round bottom flask, 3-nitrobenzaldehyde (10 g, 66.17 mmol) was charged with dithiooxamide (1.58 g, 13.2 mmol) and 20 mL of dimethylformamide. The flask was equipped with a Vigoreux column and the mixture heated at 155 °C for two hours. After this time, 80 mL of ethanol were added while still hot and the mixture vigorously stirred for 10 minutes. A yellow golden solid precipitated that was filtered and washed with ethanol and diethyl ether. The solid 3-bisnitrophenylthiazolo [5,4-d] thiazole is a bright yellow solid that is almost insoluble in most common organic solvents and was used for the next step without further purification. Following a modified procedure in the literature,[15] 3-bisnitrophenylthiazolo [5,4-d] thiazole (7.16 g, 18.65 mmol) was added to a 250mL round bottom flask and suspended in 50mL of n-butanol. To the suspension, metallic tin (22.13 g, 186.5 mmol) and 55mL of 37% HCl were slowly added and the reaction stirred at 100°C for 16h. During this time a change in color to a paler yellow was observed. The mixture was allowed to cool to room temperature and the solids filtered. A beige solid was obtained that was washed with small amounts of cold water and ethanol (5.48 g, 90%). The product 3-bisaminophenylthiazolo [5,4-d] thiazole was further attempted to convert to the isatin derivative using standard Sandmeyer, Martinet and Stolle procedures, obtaining multiple products and intractable tars in all cases.

Scheme S1. Failed attempt of preparation of **4** starting from 3-nitrobenzaldehyde.

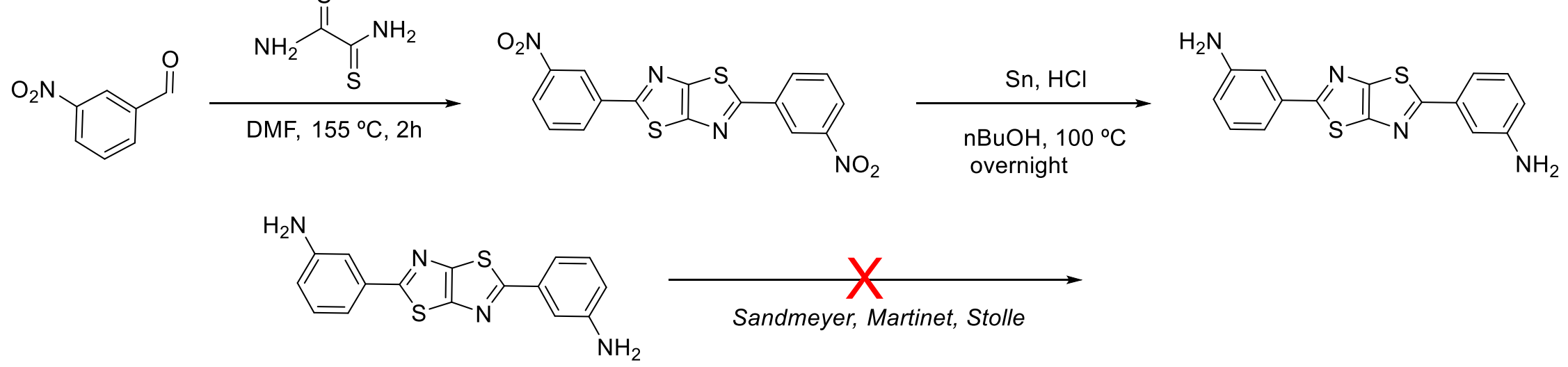

Attempts to prepare 6,6'-bisisatinthiazolo [5,4-d] thiazole *via* 6-bromoisatin derivatives.

N-alkylated 6-bromoisatin (2g, 5.9 mmol) or ketone-protected, non-alkylated 6-bromoisatin (2g, 6.4 mmol) were charged in a 250 mL two neck vacuum dried round bottom flask and subjected to vacuum for 30 minutes. After this time, three vacuum argon cycles were performed, and 20 mL of pre dried diethyl ether were added. The reaction was placed in an acetone-nitrogen bath (-78 °C) and the mixture was further sparged with argon for 5 minutes. After this time *n*-butyl lithium (1.0M in hexanes, 2.5 eq) was added dropwise *via* syringe. The mixture was stirred for 30 minutes and then dry formylating agent (dimethylformamide or N-formylpiperidine, 1.5 eq) were added dropwise *via* syringe. The reaction was allowed to reach room temperature and stirred overnight. Only decomposition and unidentified products could be obtained.

6-formylisatin could be accessed *via* 6-formylindole oxidation using iodine pentoxide as reported elsewhere[16], however, condensation with dithiooxamide yielded intractable tars and the monomer could not be reached *via* this route either.

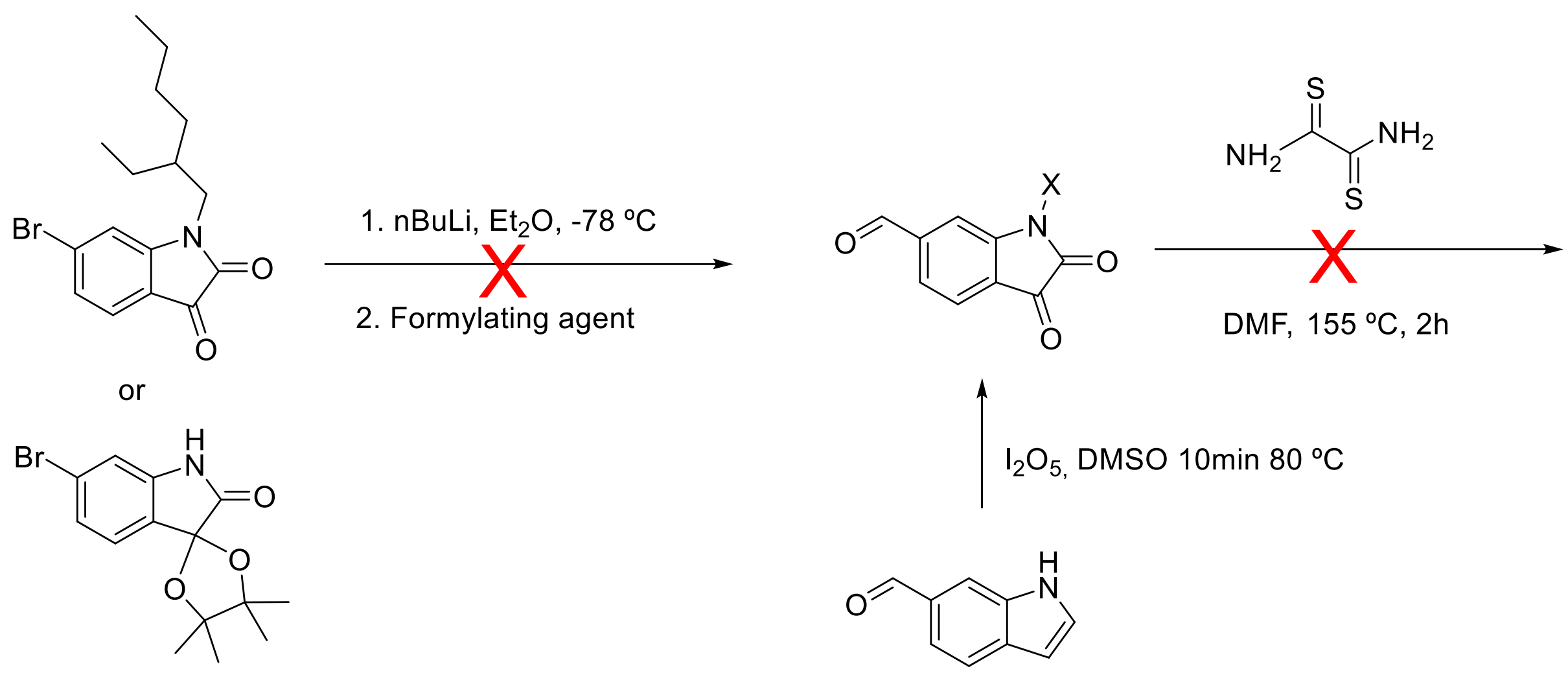

Scheme S2. Failed attempts at the preparation of **4** *via* 6-formylisatin.

Synthesis of 2

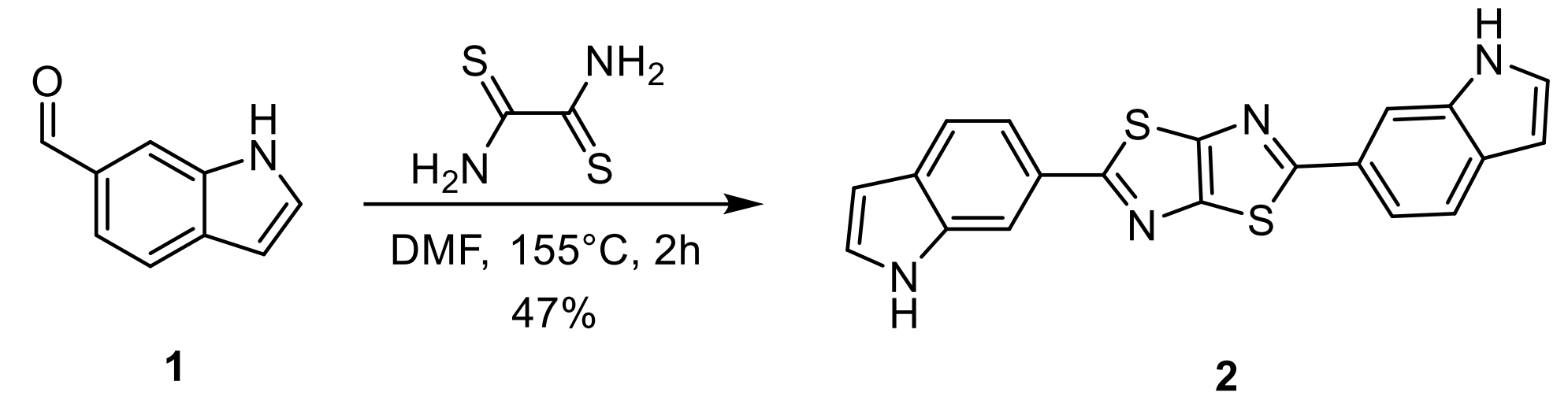

A 100 mL round bottom flask was charged with 6-formylindole (5 g, 34.4 mmol), dithiooxamide (800 mg, 6.6 mmol) and 7 mL of dimethylformamide. The red solution was heated to 155°C under air in an oil bath with strong stirring and the flask was then equipped with a Vigoreux column. After 2h, the flask was removed from the oil bath and 80 mL of ethanol were added while the reaction mixture was hot. A golden yellow precipitate immediately appears. The precipitate was filtered under vacuum and washed with copious amounts of ethanol and diethyl ether. Finally, it was dried under vacuum to yield **2** as a bright yellow powder that was pure enough to carry on to the next step. (1.15 g, 47%). Longer reaction times, or higher temperatures led to decomposition products and tars that were difficult to purify. Attempts to scale up the reaction by increasing the amounts of starting materials led to lower yields, therefore the scale up of **2** was done in 5 g batches of 6-formylindole.

Synthesis of 3

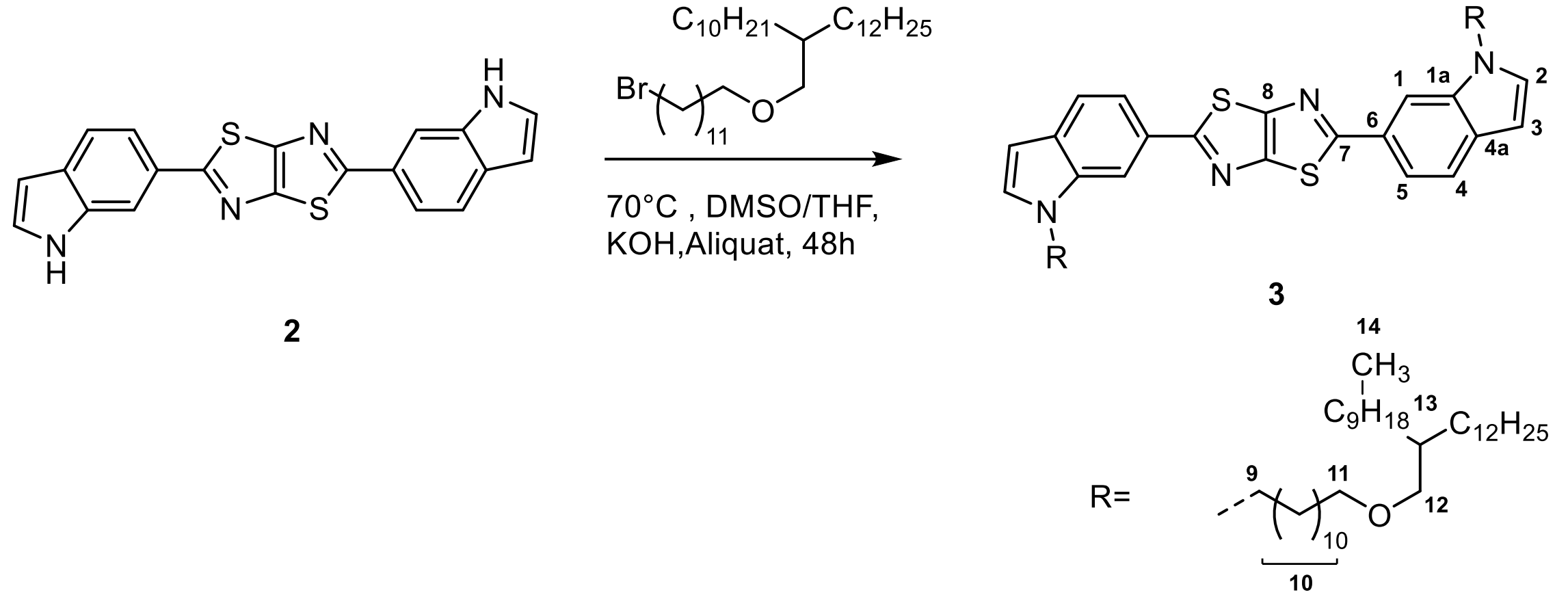

In a 250 mL round bottom flask, **2** (0.9 g, 2.4 mmol) was suspended in 15 mL of DMSO. Potassium hydroxide (0.54 g, 9.62 mmol) was finely ground in a mortar and pestle and quickly transferred to the round bottom flask. The addition of the base caused a color change from bright golden yellow to deep red due to deprotonation. The mixture was further stirred for one hour at room temperature and the alkylating agent (5.8 g, 9.62 mmol) was dissolved in 15mL of THF and added to the round bottom flask. Two drops of Aliquat 336 were added as phase transfer reagent and the mixture was stirred at 70°C for 48h. After this time, the crude mixture was diluted with 200mL of chloroform and filtered through a pad of celite to eliminate insoluble base and non-alkylated products. The filtrate was washed 5 times with 200mL of water and the organic phase collected and evaporated under reduced pressure. The resulting bright yellow oil was dissolved in the minimum amount of hexane and precipitated into 200mL of cold acetone. The precipitate was collected by filtration and the process repeated until a yellow powder was obtained. Product **3** was used in the next step without further purification.

^{1}H NMR (600.1 MHz, $CDCl_3$) δ / ppm 8.12 (s, 1 H, -C$\underline{H}_{(1)}$), 7.68 (d, $^3J_{HH}$ = 0.91 Hz 2 H, C$\underline{H}_{(5,4)}$), 7.23 (d, $^3J_{HH}$ = 3.10 Hz 1 H, C$\underline{H}_{(3)}$), 6.53 (d, $^3J_{HH}$ = 3.10 Hz, 1 H, C$\underline{H}_{(2)}$), 4.22 (t, $^3J_{HH}$ = 7.41 Hz, 2 H, C$\underline{H}_{(9)}$), 3.35 (t, $^3J_{HH}$ = 7.06 Hz, 2 H, C$\underline{H}_{(11)}$), 1.94-1.11 (m, 62 H, C$_{\underline{H,H2}(10,13)}$), 0.87 (t, $^3J_{HH}$ = 7.04 Hz, 6 H, C$\underline{H}_{3(14)}$).

^{13}C{^{1}H} NMR (150.9 MHz, $CDCl_3$) δ / ppm 170.44 (C_7), 136.1 (C_{1a}), 130.8 (C_{4a}), 130.6 (C_3),127.5 (C_8) 127.0 (C_6), 121.5 ($C_{5,4}$), 118.2 ($C_{5,4}$), 107.6 (C_1), 101.6 (C_2),74.2 (C_{12}), 71.1 (C_{11}),46.6(C_9), 38.3-22.3 ($C_{10,12,13}$), 14.1 (C_{14}).

Synthesis of 4

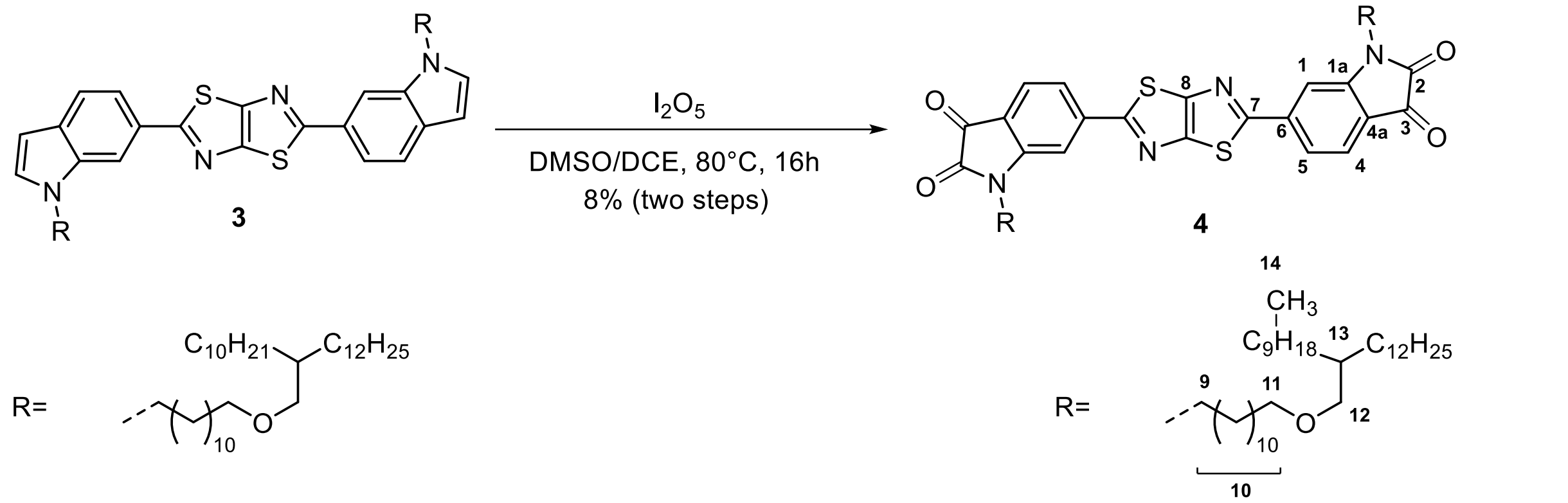

In a 250 mL round bottom flask crude **3** was suspended in 20 mL of a 1:1 mixture of dimethyl sulfoxide / dichloroethane. Then iodine pentoxide (4.79 g, 14.4 mmol, 6 equivalents respect to **2**) was added and the mixture stirred at room temperature for five minutes. After this time, the reaction was heated to 80°C and a Vigoreux column was attached to the flask. After 16 hours, the reaction was poured into 300mL of a saturated sodium thiosulfate solution, stirred for one hour and the brown precipitate collected using a cellulose thimble. The thimble was then washed with copious amounts of water and methanol and charged into a Soxhlet apparatus. After purification of 24h with methanol, the solvent reservoir was changed to acetone and the Soxhlet extraction was continued for 48h. The acetone fraction was collected and the solvent removed under reduced pressure to yield a bright orange oil that can be further purified by successive reprecipitations from dichloromethane in cold acetone to obtain a dark red waxy solid that is further purified using column chromatography ramping acetone from 0 to 1% in DCM (0.285g, 8% two steps)

CAUTION: This reaction must not be performed in closed systems. Attempts to increase oxidant concentration and/or temperature resulted in violent gas evolution which leads to explosion of closed vials and/ or overflowing over condensers (possibly due to a Kornblum-type oxidation mechanism where dimethyl sulfide is violently given off).

^{1}H NMR (600.1 MHz, $CDCl_3$) δ / ppm 7.72 (d, $^3J_{HH}$ = 7.63 Hz 1 H, -C$\underline{H}_{(4)}$), 7.67 (d, $^3J_{HH}$ = 7.63 Hz 1 H, C$\underline{H}_{(5)}$), 7.66 (s,1 H, C$\underline{H}_{(1)}$), 3.83 (t, $^3J_{HH}$ = 7.64 Hz, 2 H, C$\underline{H}_{2(9)}$), 3.35 (t, $^3J_{HH}$ = 6.59 Hz, 2 H, C$\underline{H}_{2(11)}$), 3.24 (d, $^3J_{HH}$ = 6.14 Hz, 2 H C$\underline{H}_{2(12)}$), 1.82-1.17 (m, 62 H, C$\underline{H,H2}_{(10,13)}$) ,0.87 (t, $^3J_{HH}$ = 7.04 Hz, 6 H, C$\underline{H}_{3(14)}$).

$^{13}C\{^{1}H\}$ NMR (150.9 MHz, $CDCl_3$) δ / ppm 182.7 (C_3), 167.9 (C_7), 157.9 (C_2), 152.7 (C_8), 151.5(C_{1a}), 142.2 (C_6), 126.4 (C_4), 121.9 (C_5), 118.9 (C_{4a}), 107.9 (C_1),74.1 (C_{12}), 71.1 (C_{11}), 40.6 (C_9), 38.6-21.2 ($C_{10,12,13}$), 13.7 (C_{14})

General procedure for the monofunctional model reactions

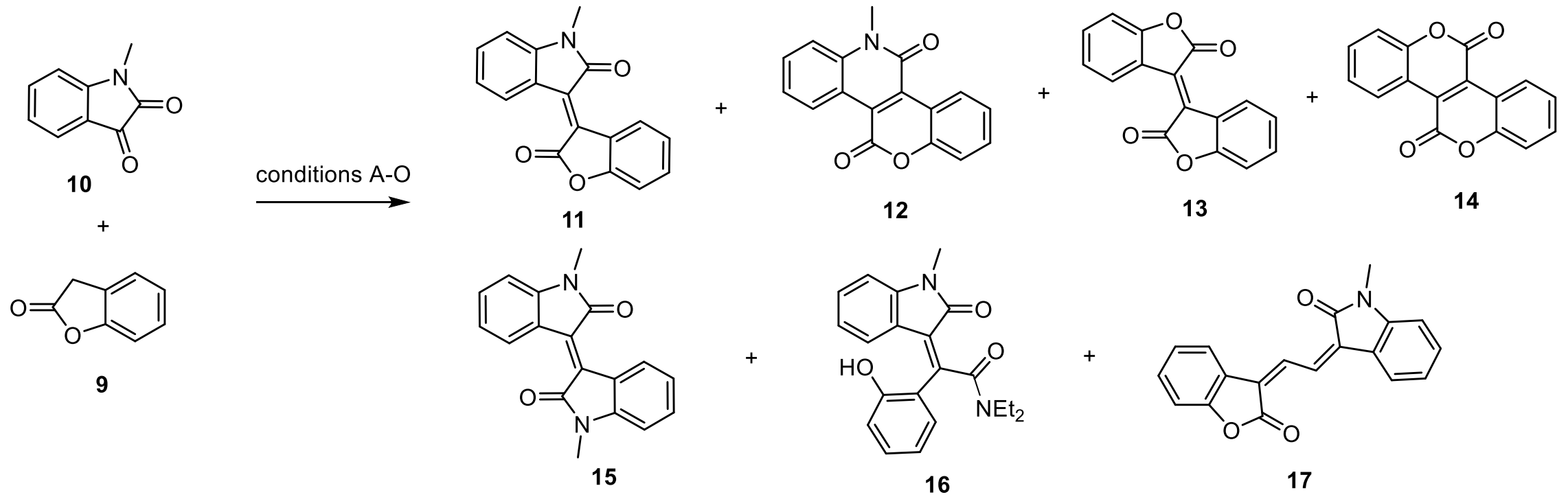

In a 15 mL snap-cap vial, *N*-methylisatin and 2-coumaranone were charged in a 1:1 molar ratio with catalyst (20% mol) and additives. To perform the reaction under inert atmosphere, the vial was evacuated and refilled five times with argon. Toluene was added *via* syringe until the final concentration of the monomers reached 0.25M. To perform the reaction under ambient conditions, the purging procedure was omitted. The solution was then stirred at 130°C for 40h in an aluminum block. After this time, the crude product was cooled, mixed with celite and evaporated to dryness under reduced pressure. The adsorbed mixture was then purified using an automated Biotage system with Biotage Sfär Silica D – Duo 60 mm 50g (for 2.75 mmol of starting materials) cartridges ramping THF in toluene from 0% to 100%. The ramp profile was as follows: 2 Equilibration Column Volumes (CV) 1 CV 0% THF, 9CV 0% to 3% THF, 1CV 3% to 6% THF, 2CV 6% to 10% THF, 2 CV 10% THF to 15% THF, 1 CV 15% to 80% THF, 1CV 80% THF to 100% THF, and finally the column is flushed with 100% THF until no further material is eluted. Every separate fraction was collected and concentrated under reduced pressure.

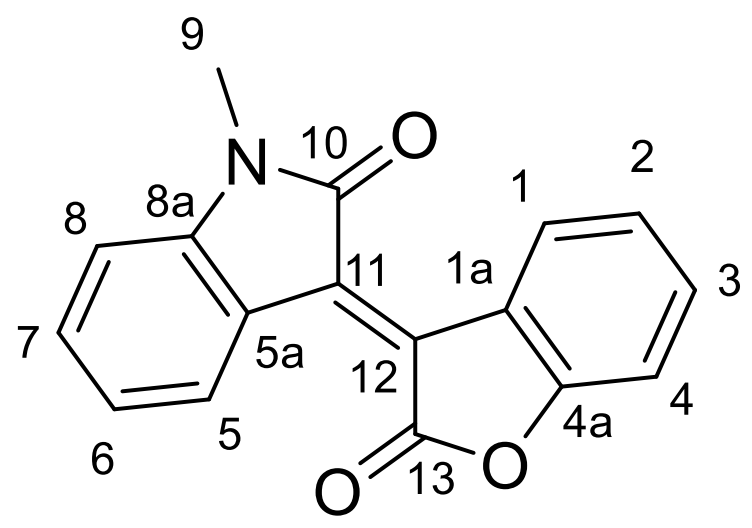

Compound **11** was collected as a burgundy fluffy solid.

^{1}H NMR (600.1 MHz, $CDCl_3$) δ / ppm 9.16 (dd, $^{3}J_{HH}$ = 8.18 Hz, $^{4}J_{HH}$ = 0.96 Hz 1 H, -C$\underline{H}_{(1)}$), 8.89 (dd, $^{3}J_{HH}$ = 8.08 Hz, $^{4}J_{HH}$ = 0.61 Hz 1 H, -C$\underline{H}_{(5)}$),7.36 (td, $^{3}J_{HH}$ = 7.60 Hz, $^{4}J_{HH}$ = 1.31 Hz 1 H, C$\underline{H}_{(3)}$), 7.28 (td, $^{3}J_{HH}$ = 7.64 Hz, $^{4}J_{HH}$ = 1.15 Hz 1 H, C$\underline{H}_{(7)}$), 7.10 (td, $^{3}J_{HH}$ = 7.71 Hz, $^{4}J_{HH}$ = 1.11 Hz 1 H, C$\underline{H}_{(2)}$), 7.01 (dd, $^{3}J_{HH}$ = 7.98 Hz, $^{4}J_{HH}$ = 0.66 Hz 1 H, -C$\underline{H}_{(4)}$), 6.97 (td, $^{3}J_{HH}$ = 7.64 Hz, $^{4}J_{HH}$ = 1.08 Hz 1 H, C$\underline{H}_{(6)}$), 6.67 (d, $^{3}J_{HH}$ = 7.76 Hz, 1 H, -C$\underline{H}_{(8)}$),3.16 (s, 1H, -C$\underline{H}_{3(9)}$).

^{13}C{^{1}H} NMR (150.9 MHz, $CDCl_3$) δ / ppm 167.4 (C_{10}), 167.3 (C_{13}), 155.3 (C_{4a}), 145.7 (C_{8a}),135.2(C_{11}) 133.4 (C_4), 133.2 (C_7), 130.0 (C_1), 129.8 (C_5), 127.0 (C_{12}), 124.1 (C_2), 123.0 (C_{1a}), 122.7 (C_6), 120.9 (C_{5a}), 110.4 (C_4). 108.0 (C_8), 26.2 (C_9).

MALDI-TOF (m/z) (% relative intensity, ion): $[M]^+$ calculated: 277.07, observed: 276.93.

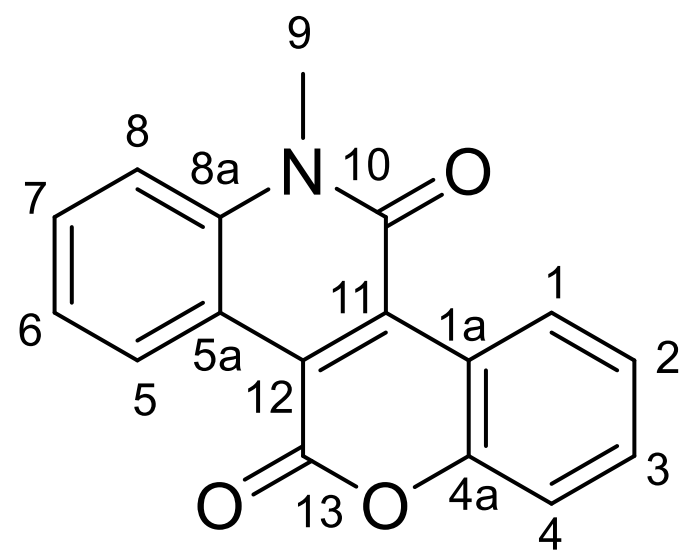

Compound **12** was collected as a bright yellow solid that fluoresces strongly under a UV light. It presents a noticeable decrease in solubility in comparison to **11**, therefore NMR analysis was conducted using $C_2D_2Cl_4$.

^{1}H NMR (600.1 MHz, $C_2D_2Cl_4$) δ / ppm 9.56 (dd, $^{3}J_{HH}$ = 8.38 Hz, $^{4}J_{HH}$ = 1.57 Hz 1 H, -C$\underline{H}_{(1)}$), 9.40 (dd, $^{3}J_{HH}$ = 8.38 Hz, $^{4}J_{HH}$ = 1.02 Hz 1 H, -C$\underline{H}_{(5)}$),7.65 (td, $^{3}J_{HH}$ = 7.85 Hz, $^{4}J_{HH}$ = 1.32 Hz 1 H, C$\underline{H}_{(7)}$), 7.57 (td, $^{3}J_{HH}$ = 7.63 Hz, $^{4}J_{HH}$ = 1.43 Hz 1 H, C$\underline{H}_{(3)}$), 7.43 (d, $^{3}J_{HH}$ = 8.50 Hz, 1 H, C$\underline{H}_{(8)}$), 7.38 (m, H, -C$\underline{H}_{(2,4,6)}$),3.83 (s, 1H, -C$\underline{H}_{3(9)}$).

$^{13}C\{^1H\}$ NMR (150.9 MHz, $C_2D_2Cl_4$) δ / ppm 158.8 (C_{10}), 158.4 (C_{13}), 151.2 (C_{4a}), 138.2 (C_{8a}),131.08(C_3) 130.9 (C_7), 128.8 (C_{11}), 128.4 (C_1), 127.7 (C_5), 124.3 and 123.1 ($C_{2,4\ or\ 6}$), 123.0, 116.43 and 116.40 ($C_{5a,\ 12\ or\ 1a}$), 115.6 ($C_{2,4\ or\ 6}$), 113.7 (C_8),30.1 (C_9).
MALDI-TOF (m/z) (% relative intensity, ion): $[M]^+$ calculated: 277.07, observed: 276.95.

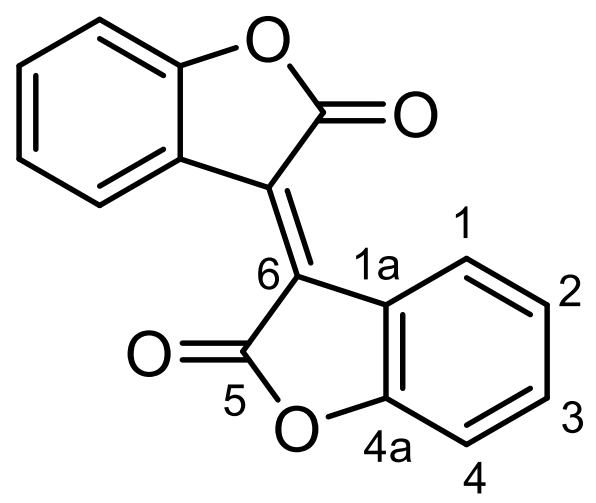

Compound **13** was collected as an orange solid. It presentes a noticeable decrease in solubility in comparison to **11**, therefore NMR analysis was conducted in $C_2D_2Cl_4$.

^1H NMR (600.1 MHz, $C_2D_2Cl_4$) δ / ppm 9.03 (dd, $^3J_{HH}$ = 8.29 Hz, $^4J_{HH}$ = 0.91 Hz 1 H, -C$\underline{H}_{(1)}$), 7.21 (td, $^3J_{HH}$ = 8.03 Hz, $^4J_{HH}$ = 1.13 Hz 1 H, C$\underline{H}_{(2)}$), 7.47 (td, $^3J_{HH}$ = 7.81 Hz, $^4J_{HH}$ = 1.18 Hz 1 H, C$\underline{H}_{(3)}$), 7.11 (d, $^3J_{HH}$ = 8.04 Hz, C$\underline{H}_{(4)}$).

$^{13}C\{^1H\}$ NMR (150.9 MHz, $C_2D_2Cl_4$) δ / ppm 166.3 (C_5), 155.2 (C_{1a}), 134.4 (C_3), 133.6 (C_6),128.9(C_1) 124.1(C_2), 122.1 (C_{4a}), 110.46 (C_4).
MALDI-TOF (m/z) (% relative intensity, ion): $[M]^+$ calculated: 264.04, observed: 264.03

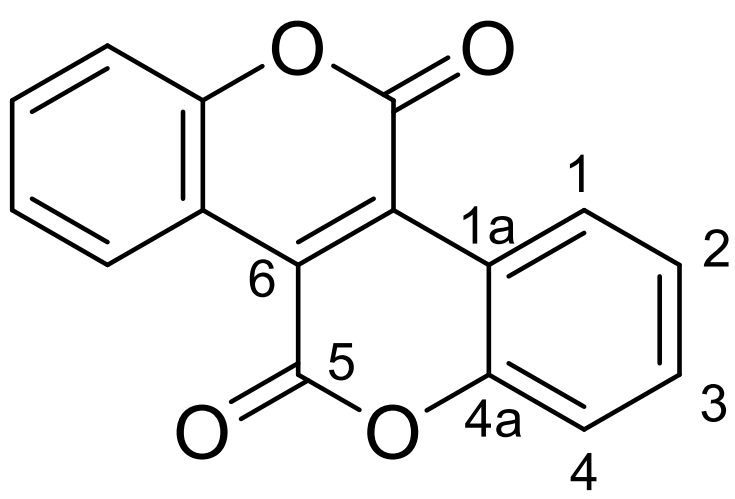

Compound **14** was collected as light-yellow needles that fluoresce strongly under UV light. It presents a noticeable decrease in solubility in comparison to **10**, therefore NMR analysis was conducted in $C_2D_2Cl_4$.

^1H NMR (600.1 MHz, $C_2D_2Cl_4$) δ / ppm 9.20 (dd, $^3J_{HH}$ = 8.33 Hz, $^4J_{HH}$ = 1.08 Hz 1 H, -C$\underline{H}_{(1)}$),7.65 (td, $^3J_{HH}$ = 7.77 Hz, $^4J_{HH}$ = 1.38 Hz 1 H, C$\underline{H}_{(3)}$), 7.45 (td, $^3J_{HH}$ = 7.84 Hz, $^4J_{HH}$ = 0.95 Hz 1 H, C$\underline{H}_{(2)}$), 7.42 (d, $^3J_{HH}$ = 8.3 Hz, 1 H, C$\underline{H}_{(4)}$)

$^{13}C\{^1H\}$ NMR (150.9 MHz, $C_2D_2Cl_4$) δ / ppm 157.2 (C_5), 151.8 (C_6), 131.8 (C_3), 127.7 (C_1),125.7(C_{1a}) 125.1 (C_2), 116.18 (C_4), 114.2 (C_{4a}).

MALDI-TOF (m/z) (% relative intensity, ion): $[M]^+$ calculated: 264.04, observed: 264.03

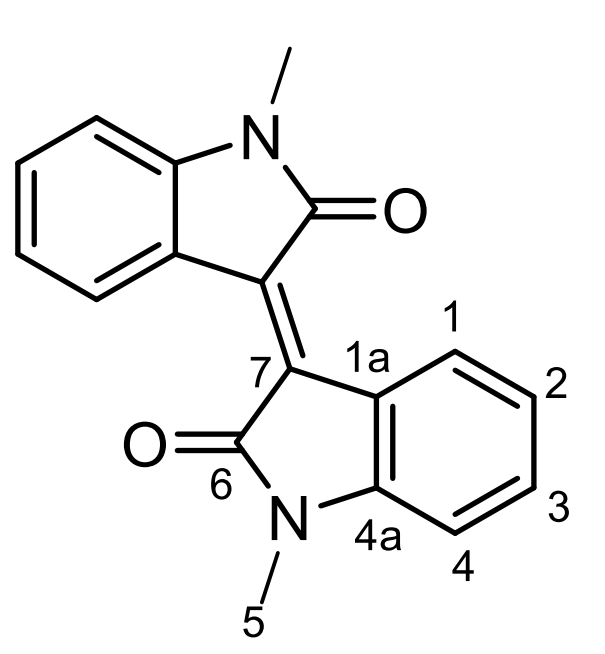

Compound **15** was collected as a dark crystalline red solid.

^{1}H NMR (600.1 MHz, $CDCl_3$) δ / ppm 9.20 (d, $^3J_{HH}$ = 7.96 Hz, 1 H, -C$\underline{H}_{(1)}$),7.61 (t, $^3J_{HH}$ = 8.08 Hz, 1 H, -C$\underline{H}_{(3)}$),6.99 (t, $^3J_{HH}$ = 7.60 Hz, 1 H, C$\underline{H}_{(2)}$), 6.70 (d, $^3J_{HH}$ = 7.65 Hz, 1 H, C$\underline{H}_{(4)}$).

^{13}C{^{1}H} NMR (150.9 MHz, $CDCl_3$) δ / ppm 167.8 (C_6), 145.2 (C_{4a}), 134.1 (C_7), 132.4 (C_3),129.8 (C_1) 122.1 (C_2), 121.5 (C_{1a}), 107,6 (C_4), 26.0 (C_5).

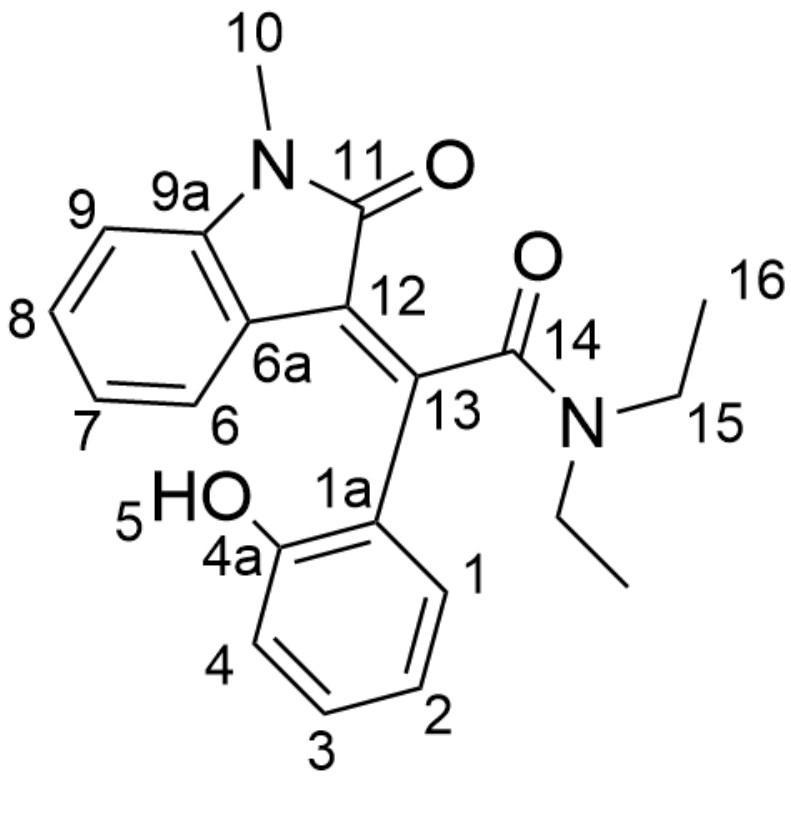

Compound **16** was collected as yellow crystals. Signal duplication indicates the presence of rotamers in solution.

^{1}H NMR (600.1 MHz, DMSO-d_6) δ / ppm 10.10 (s, 1 H, -O$\underline{H}$ (5)), 7.38 (dd, $^3J_{HH}$ = 7.74Hz, $^4J_{HH}$ = 1.68 Hz 1 H, -C$\underline{H}_{(1)}$),7.33 (t, $^3J_{HH}$ = 7.74 Hz, 1 H, -C$\underline{H}_{(3)}$),7.26 (t, $^3J_{HH}$ = 7.74 Hz, 1 H, C$\underline{H}_{(8)}$), 7.00 (d, $^3J_{HH}$ = 8.40 Hz, 1 H, C$\underline{H}_{(4)}$), 6.97 (d, $^3J_{HH}$ = 7.82 Hz, 1 H, C$\underline{H}_{(9)}$),6.92 (t, $^3J_{HH}$ = 7.55 Hz, 1 H, C$\underline{H}_{(2)}$), 6.81 (t, $^3J_{HH}$ = 7.64 Hz, 1 H, C$\underline{H}_{(7)}$), 6.65(d, $^3J_{HH}$ = 7.50 Hz, 1 H, C$\underline{H}_{(6)}$), 3.47 (m, 2H, C$\underline{H}_{2(15)}$), 3.25 (m, 2H, C$\underline{H}_{2(15)}$), 3.16 (s, 3H, C$\underline{H}_{3(10)}$), 1.11 (t, $^3J_{HH}$ = 7.26 Hz, 3 H, C$\underline{H}_{3(16)}$), 0.82 (t, $^3J_{HH}$ = 7.36 Hz, 3 H, C$\underline{H}_{3(16)}$).

^{13}C{^{1}H} NMR (150.9 MHz, DMSO-d_6) δ / ppm 167.9 (C_{14}), 165.9 (C_{11}), 154.3 (C_{1a}), 143.8 (C_{9a}),142.0 (C_{13}), 131.4 (C_3), 129.9 (C_8), 128.7 (C_1), 124.2 (C_{12}), 123.1 (C_6), 123.1 (C_6),

122.0 (C_7), 121.9 (C_{4a}), 121.8 (C_{6a}), 119.9 (C_2), 116.2 (C_4), 108.7 (C_9), 123.1 (C_6), 41.3 (C_{15}), 38.0 (C_{15}), 26.1 (C_{10}), 13.8 (C_{16}), 12.4 (C_{16}).

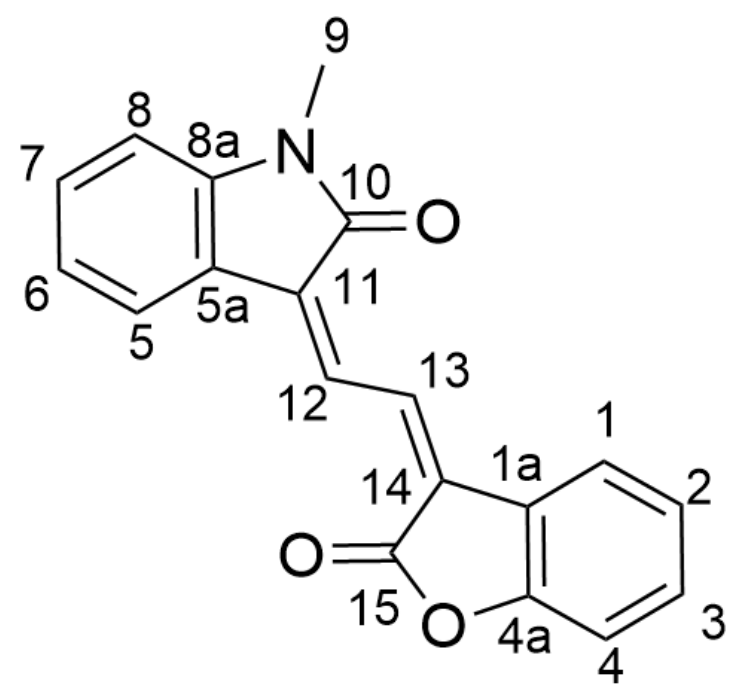

Compound **17** was collected as burgundy plates.

^{1}H NMR (600.1 MHz, DMSO-d$_6$) δ / ppm 9.14 (d, $^3J_{HH}$ = 12.7 Hz, 1 H, -C$\underline{H}_{(13)}$), 8.65 (d, $^3J_{HH}$ = 12.7 Hz, 1 H, -C$\underline{H}_{(12)}$),7.70 (d, $^3J_{HH}$ = 8.34 Hz, 1 H, C$\underline{H}_{(1)}$), 7.65 (d, $^3J_{HH}$ = 7.78 Hz, 1 H, C$\underline{H}_{(5)}$), 7.42 (t, $^3J_{HH}$ = 8.34 Hz, 1 H, C$\underline{H}_{(3)}$),7.36 (t, $^3J_{HH}$ = 7.78 Hz, 1 H, C$\underline{H}_{(7)}$), 7.23 (t, $^3J_{HH}$ = 7.78 Hz, 1 H, C$\underline{H}_{(2)}$), 7.14(d, $^3J_{HH}$ = 8.34 Hz, 1 H, C$\underline{H}_{(4)}$), 7.08 (t, $^3J_{HH}$ = 7.78 Hz, 1 H, C$\underline{H}_{(6)}$), 6.84 (d, $^3J_{HH}$ = 7.78 Hz, 1 H, C$\underline{H}_{(8)}$) 3.25 (s, 3H, C$\underline{H}_{3(9)}$).

^{13}C{^{1}H} NMR (150.9 MHz, DMSO-d$_6$) δ / ppm 166.6 (C_{10}), 166.2 (C_{15}), 154.1, 143.9, 132.6, 125.6, 124.3, 122.7($C_{1a,4a,14,11,5a,8a}$), 131.5 (C_{13}), 131.4 (C_3), 131.2 (C_7), 126.7 (C_{12}), 124.1 (C_2), 122.2 (C_4), 121.3 ($C_{1,5}$), 110.7 (C_6), 108.3 (C_8), 25.6 (C_9).

3. Additional Figures

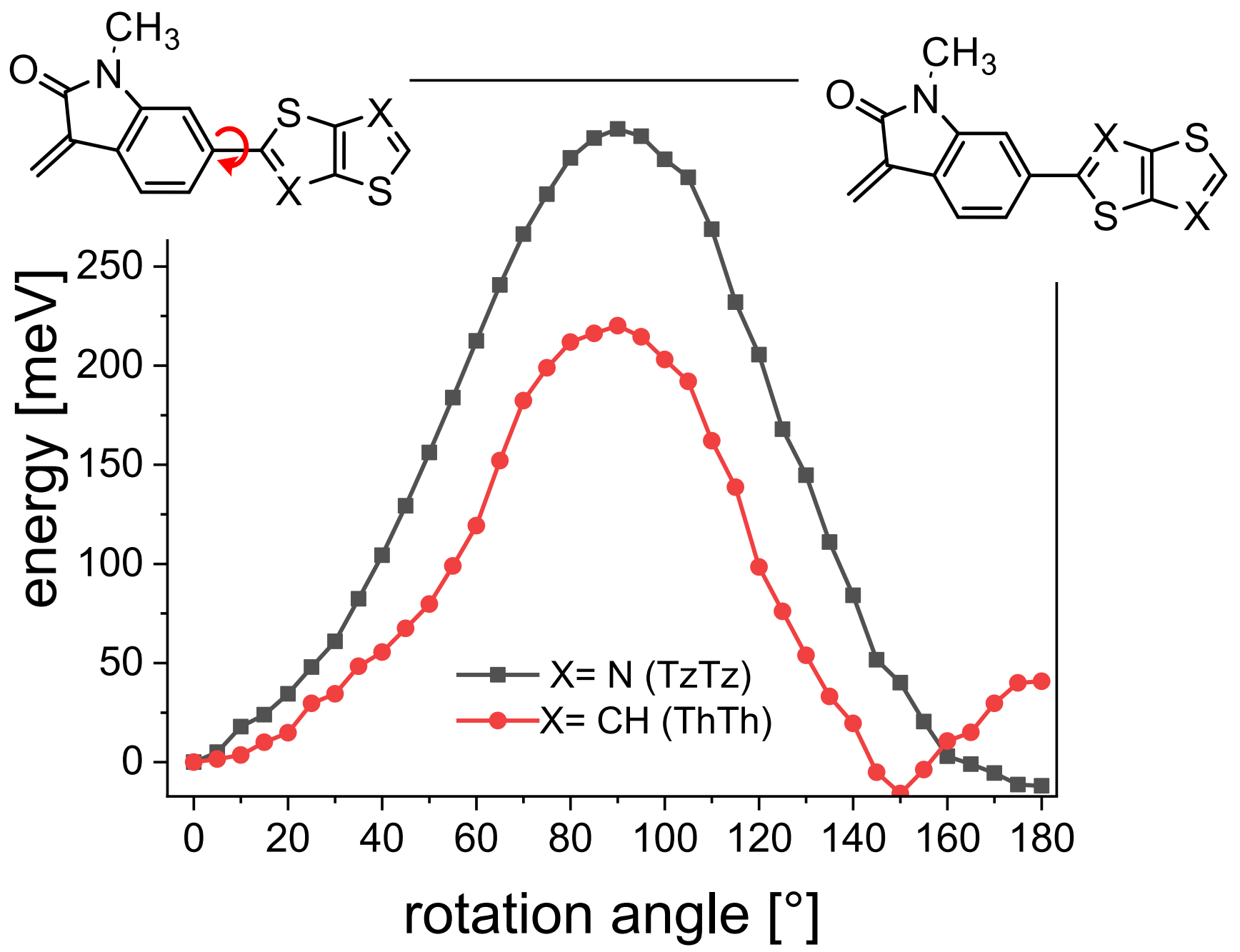

Figure S1. Calculated isatin-thienothiophene and isatin-thiazolothiazole torsion potentials.

To assess the planarity of the motifs that bridge two isatin units in BDF-isatin based polymers, we studied the rotational potential between isatin and thienothiophene (ThTh, X= CH) and thiazolothiazole (TzTz, X= N) by density functional theory. For this, molecular models were generated and optimized by keeping the rotational angle constant. For the TzTz unit a nearly symmetric rotational potential curve is obtained with a rotational barrier of about 320 meV at 90° rotation where conjugation is fully broken. A small preference towards anti-orientation is observed, i.e. with the TzTz-nitrogen pointing into opposite direction as the isatin-nitrogen. For the ThTh model, a stronger asymmetry is found with a lower barrier of about 230 meV at 90°. A second barrier of about 50 meV is seen at 180° for the planar anti-orientation. The minimum configuration is found for a rotation angle of about 30° in anti-orientation. This confirms the hypothesis mentioned in the main text, that TzTz guaranties enhanced coplanarity compared to the ThTh analog due to reduced steric repulsion. The asymmetry in steric repulsion between the syn and anti-conformations in the ThTh model is associated with the asymmetric functionalization of the six-membered ring. This asymmetry slightly shifts the electron density to one side, favors the syn-orientation and leads to the second barrier at 180°. Since in the isostructural TzTz the CH of ThTh is replaced by N having an electron lone pair, the anti-orientation is favored.

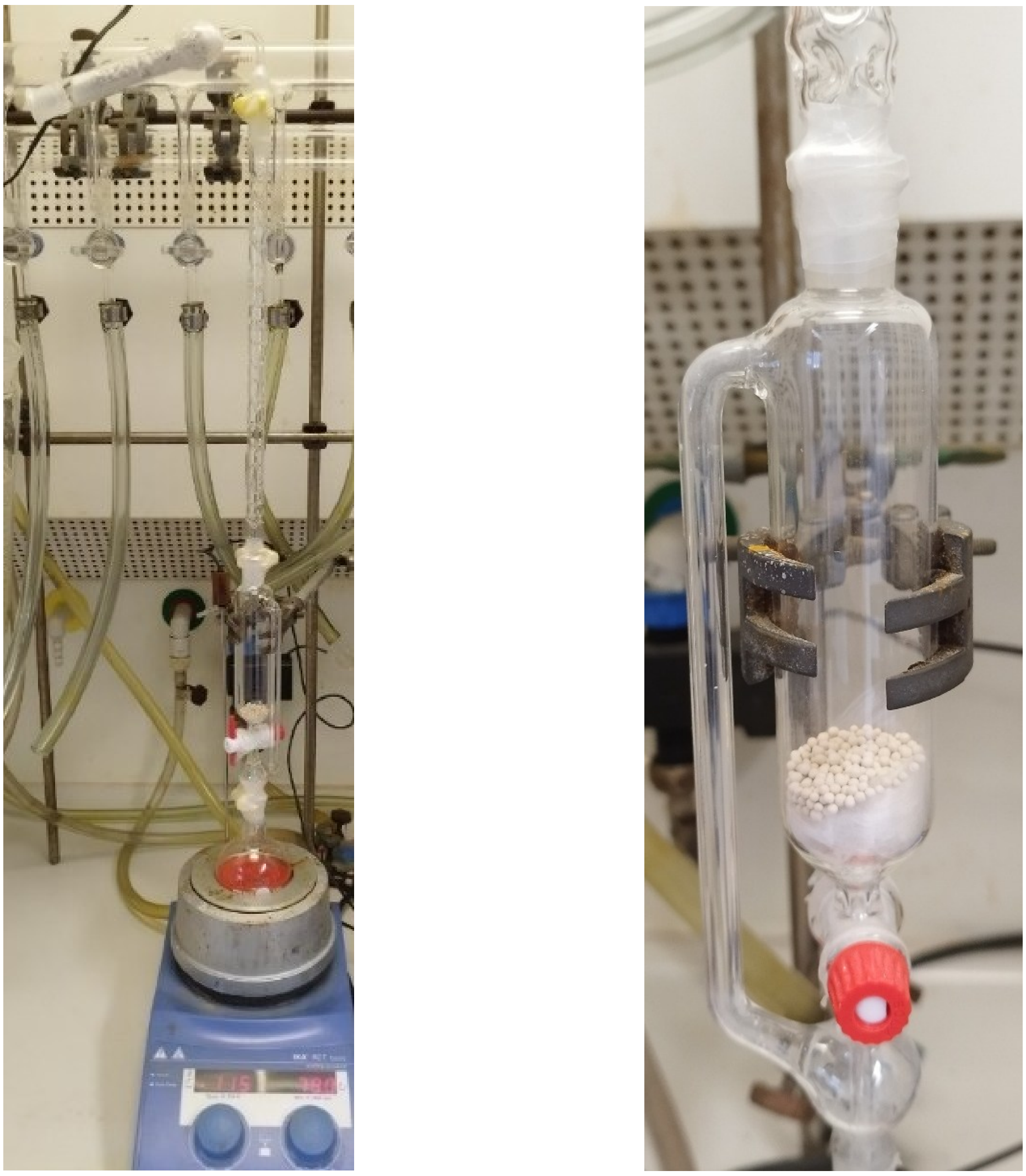

Figure S2. Image of the reaction setup for water removal.

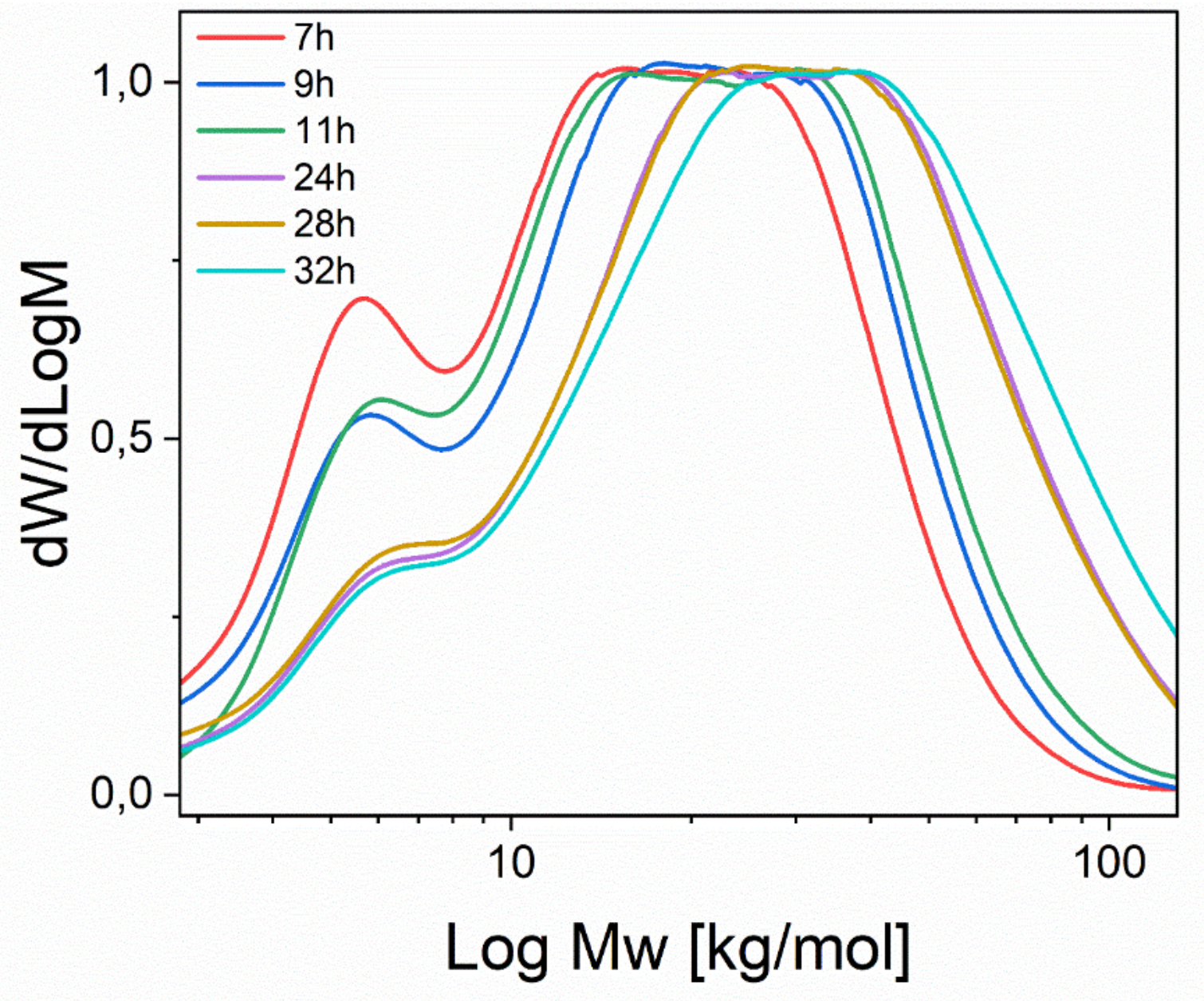

Figure S3. High temperature SEC curves of the aliquots taken during the synthesis of PBDFTzTz at different reaction times measured in 1,2,4-trichlorobenzene at 160°C.

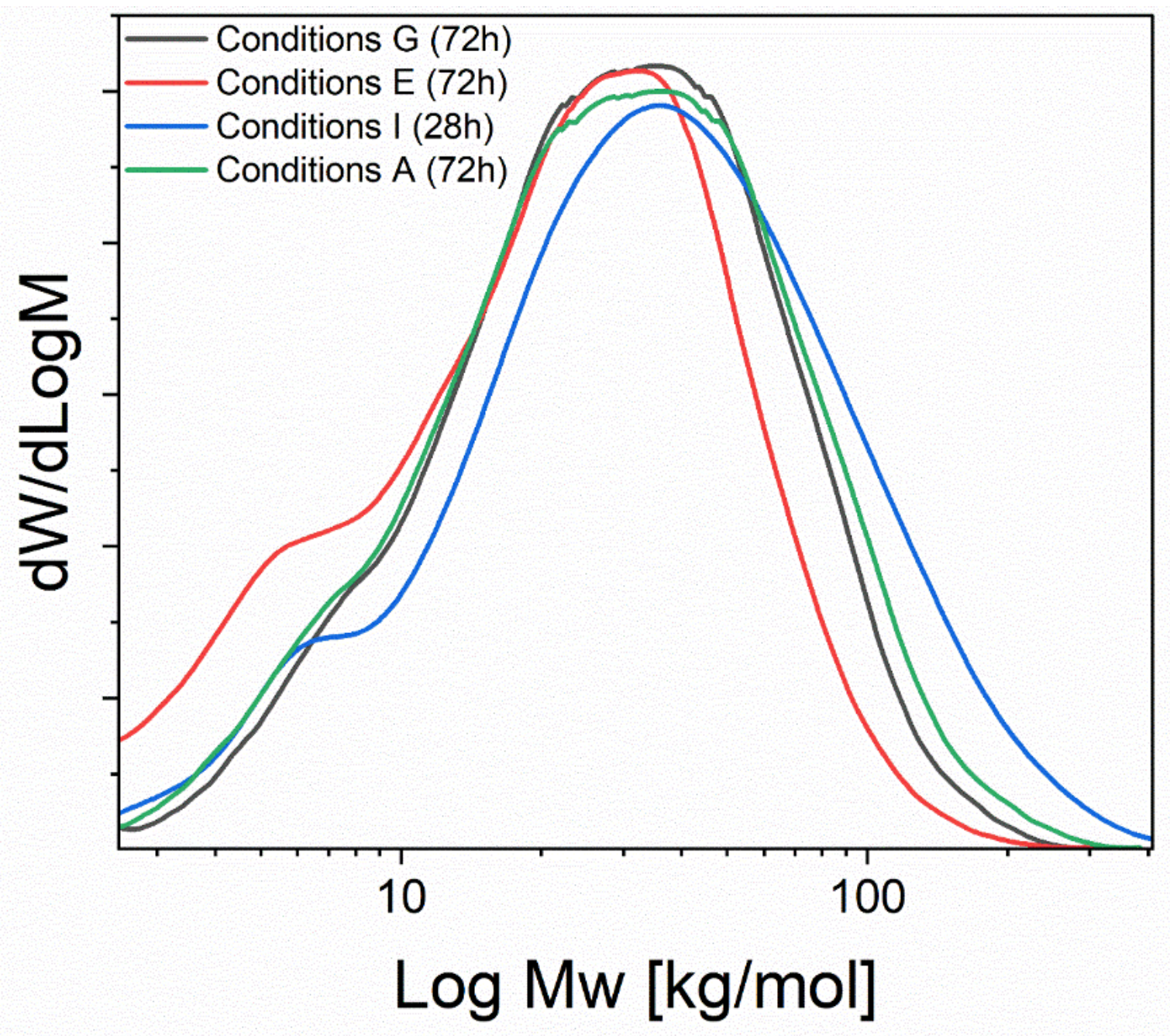

Figure S4. High Temperature SEC curves of **PBDFTzTz** synthesized under different conditions measured in 1,2,4-trichlorobenzene at 160°C.

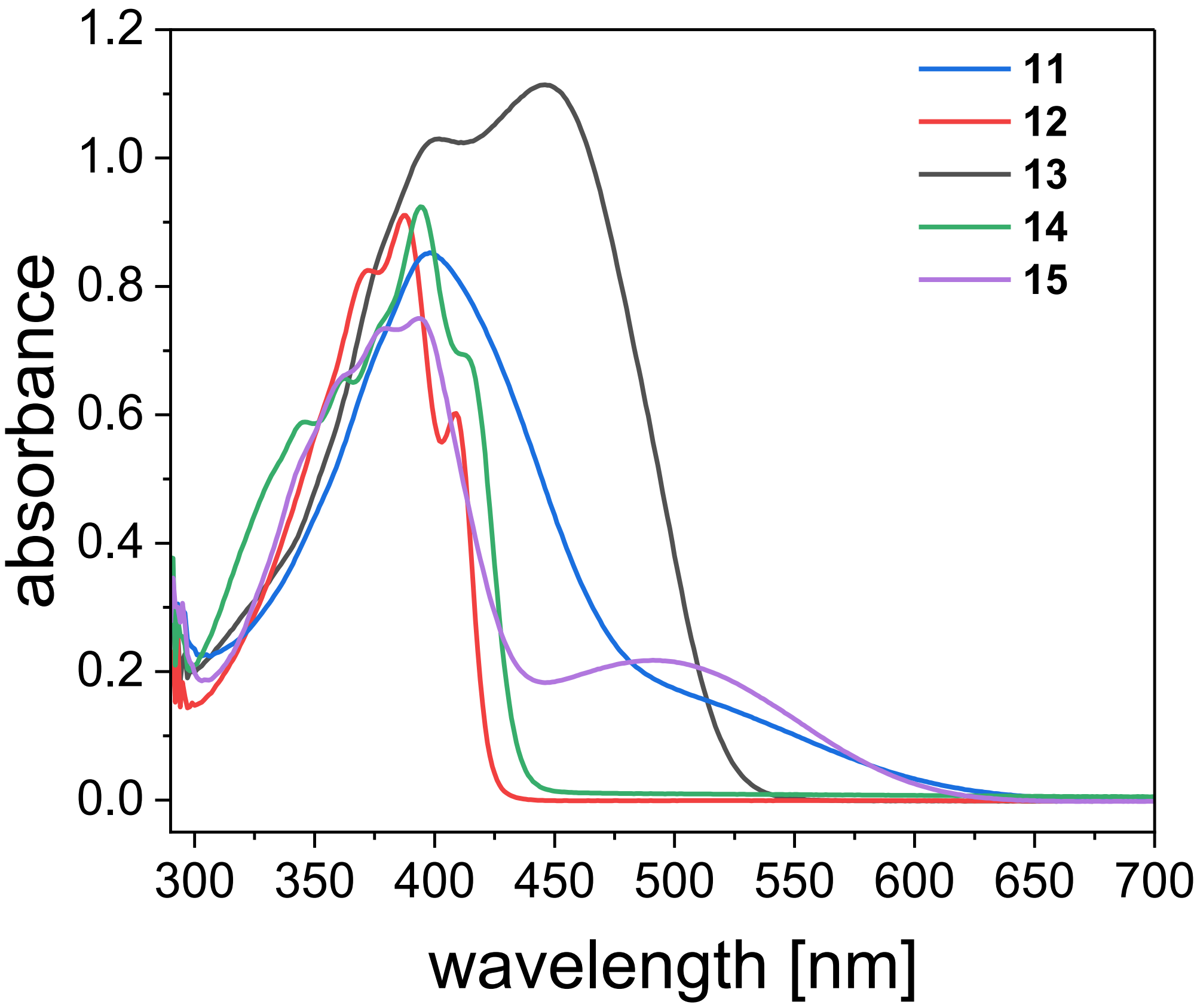

Figure S5. UV-vis spectra of isolated defects **11-15**.

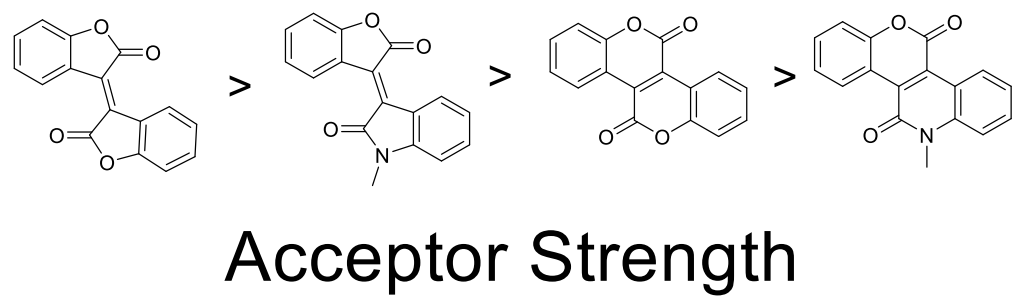

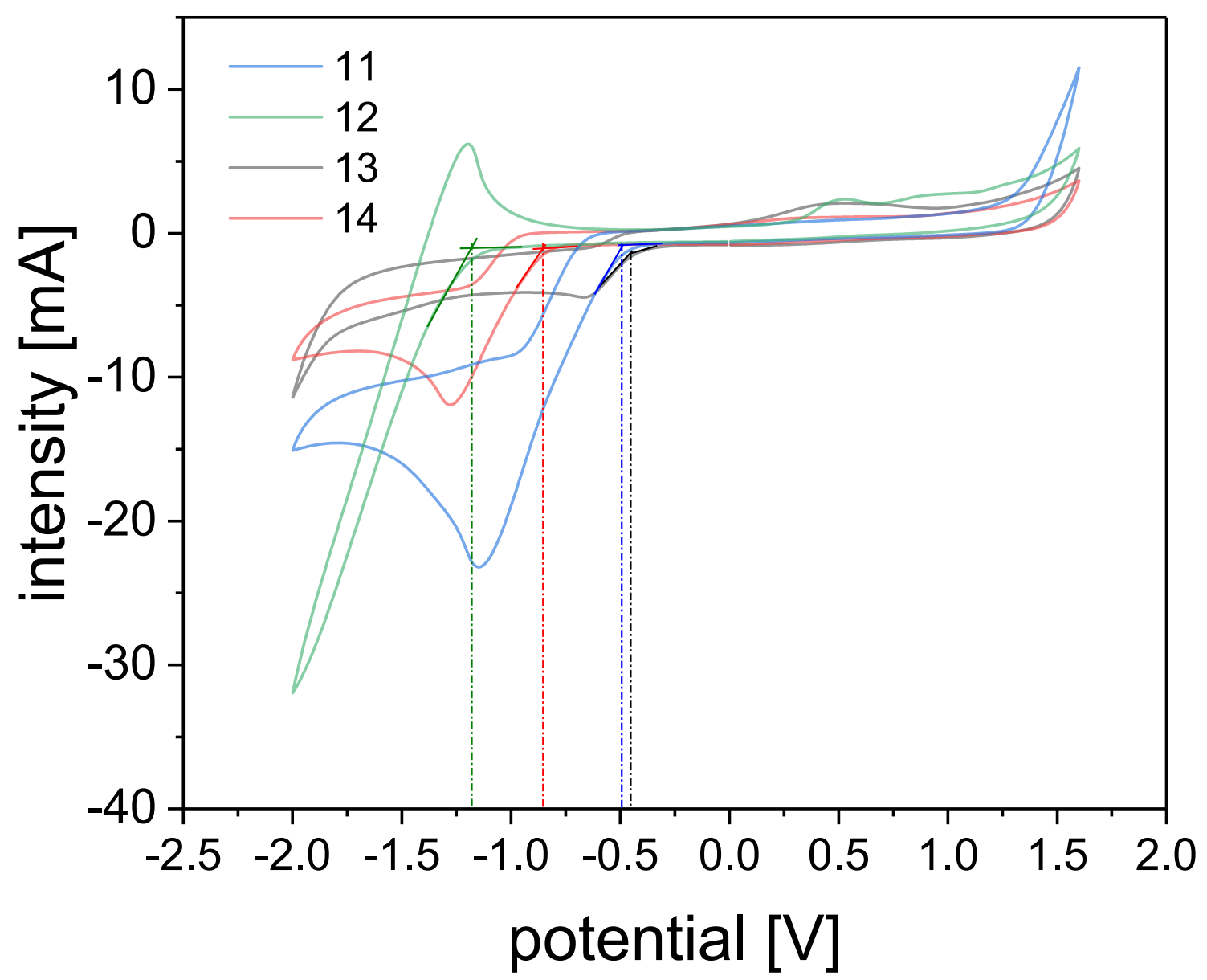

Figure S6. Cyclic voltammetry curves of model compounds isolated from model reactions in diluted *o*-DCB solution in 0.1M $NBu_4^+PF_6^-$.

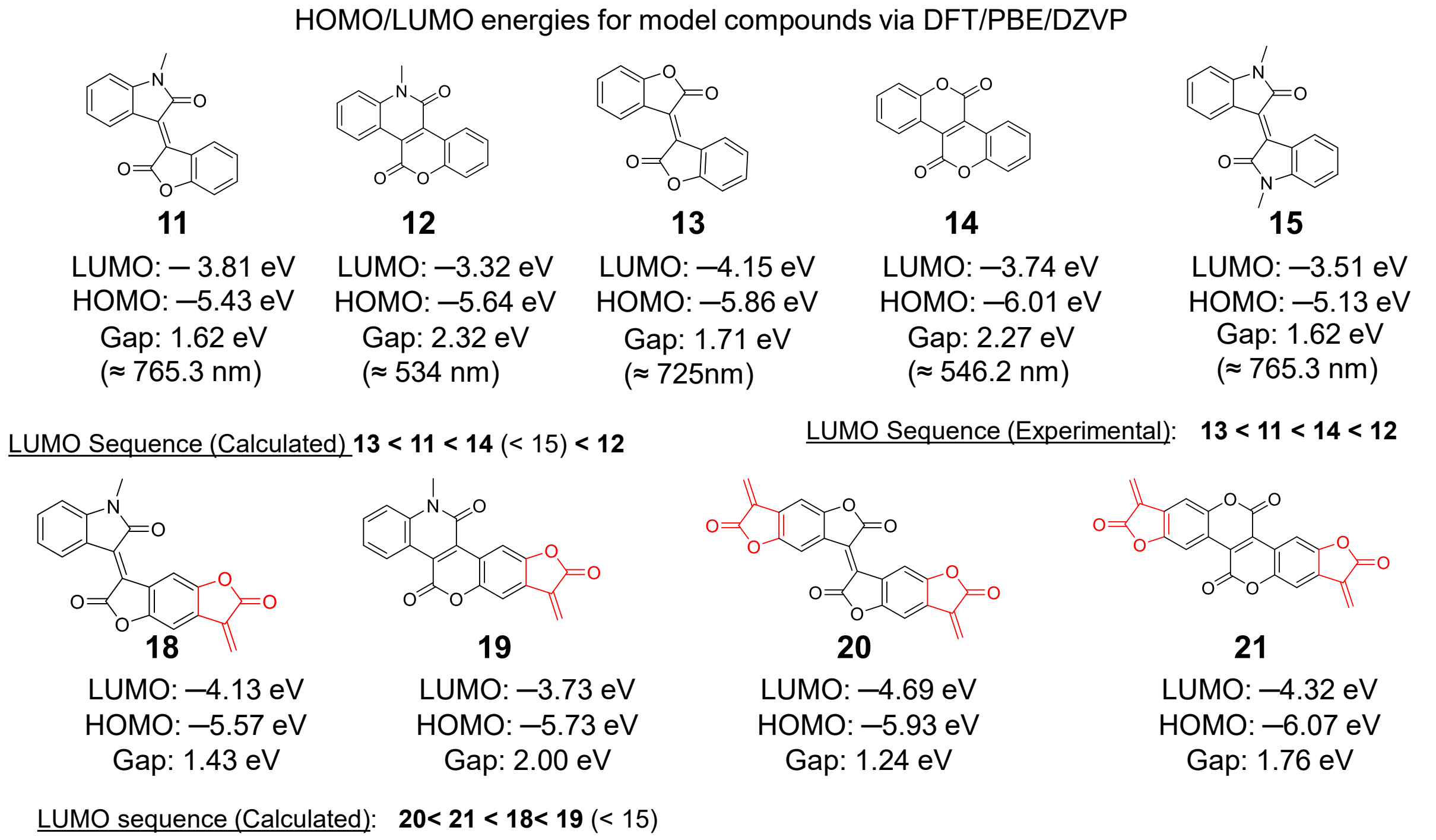

Figure S7. Calculated HOMO-LUMO levels and optical bandgaps for molecular structures **11-15** and extended defect structures **18-21.**

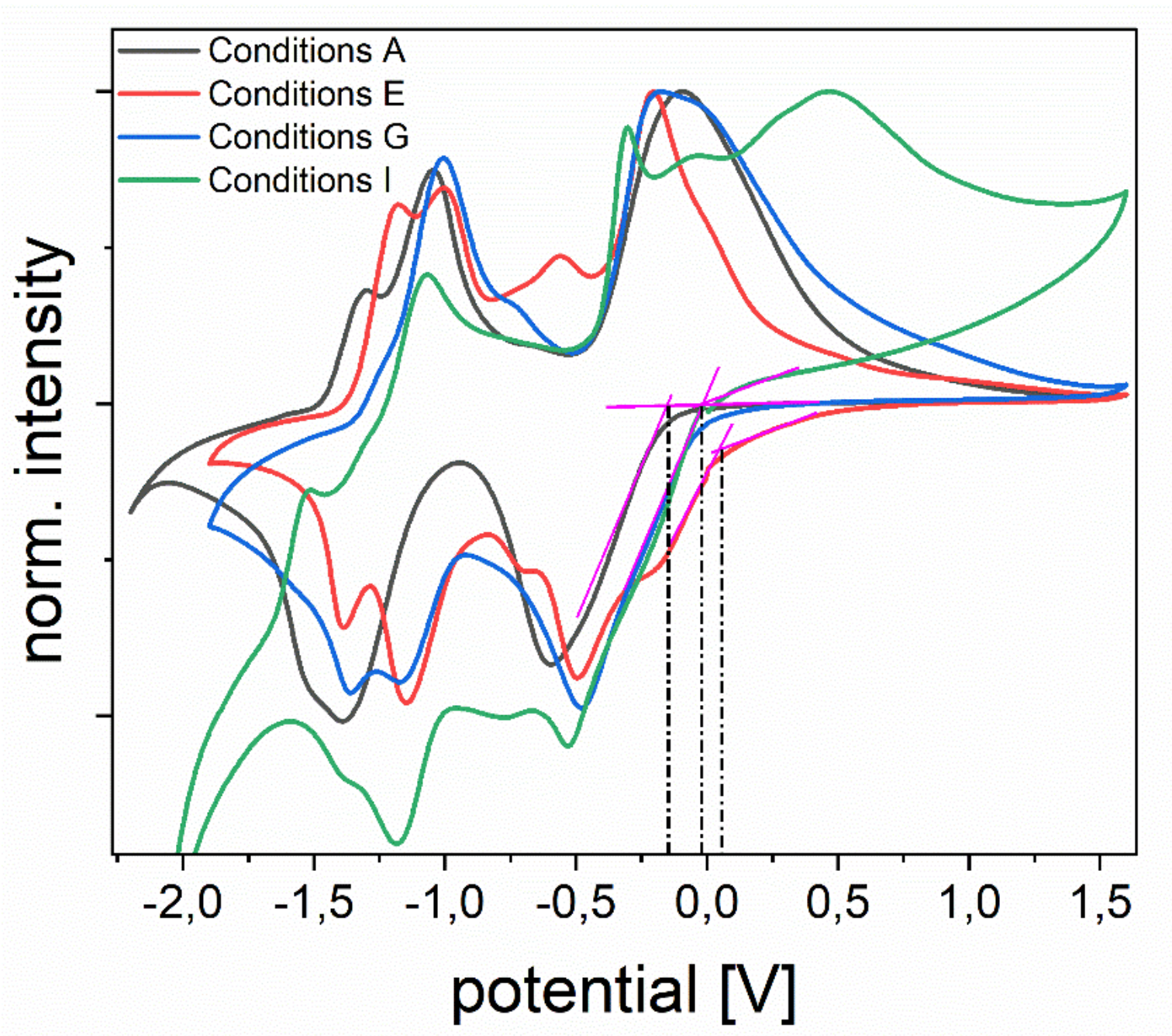

Figure S8. Film cyclic voltammetry curves of **PBDFTzTz** isolated from model reactions in ACN solution in 0.1M $NBu_4^+PF_6^-$.

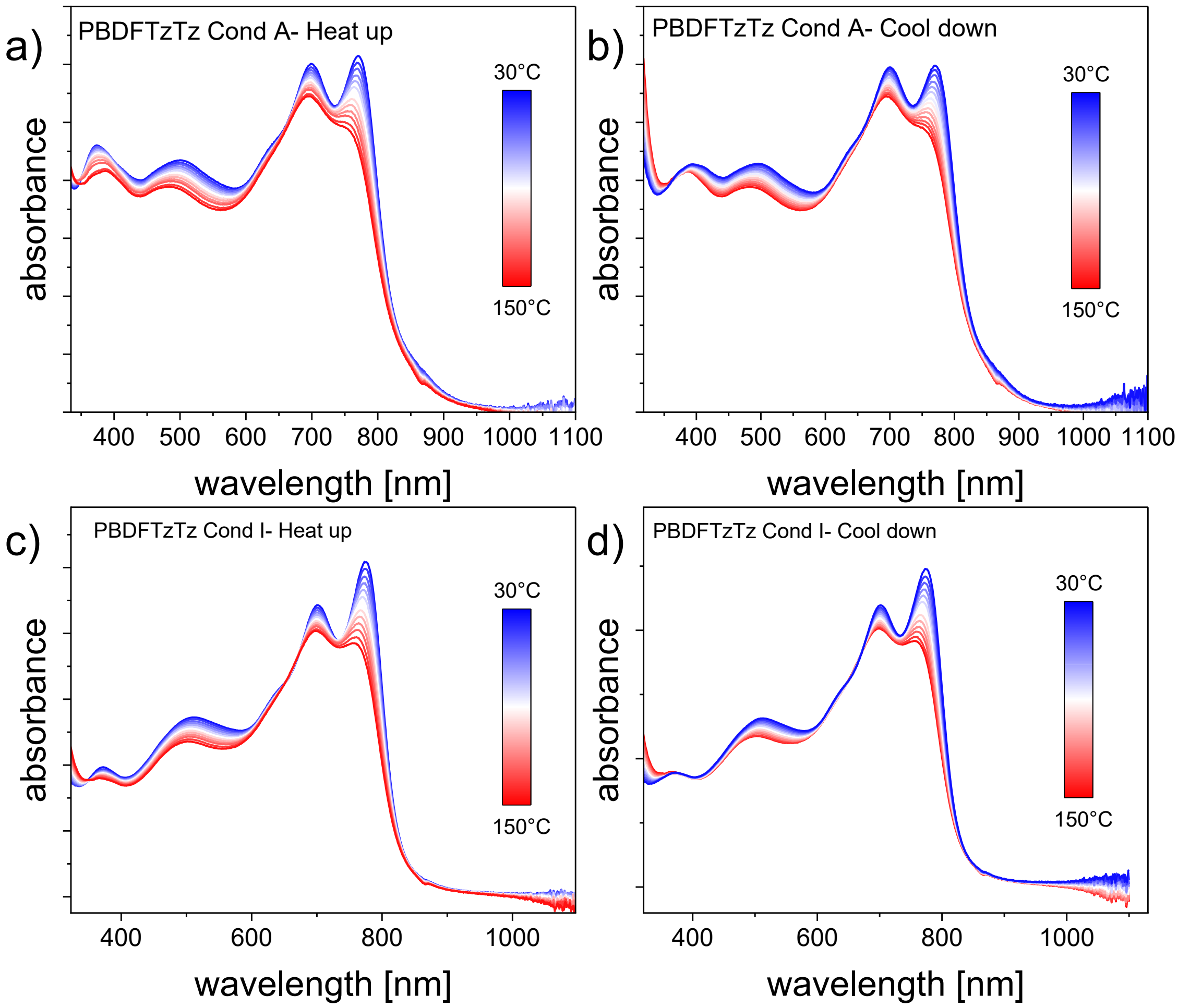

Figure S9. a) Heating UV-vis spectra of **PBDFTzTz** synthesized using conditions A in *o*-DCB from 30 °C to 150 °C. b) Cooling down UV-vis spectra of **PBDFTzTz** synthesized using conditions A in *o*-DCB from 150 °C to 30 °C. c) Heating UV-vis spectra of **PBDFTzTz** synthesized using conditions I in *o*-DCB from 30 °C to 150 °C. (Soluble fraction) d) Cooling down UV-vis spectra of **PBDFTzTz** synthesized using conditions I in *o*-DCB from 150 °C to 30 °C (soluble fraction).

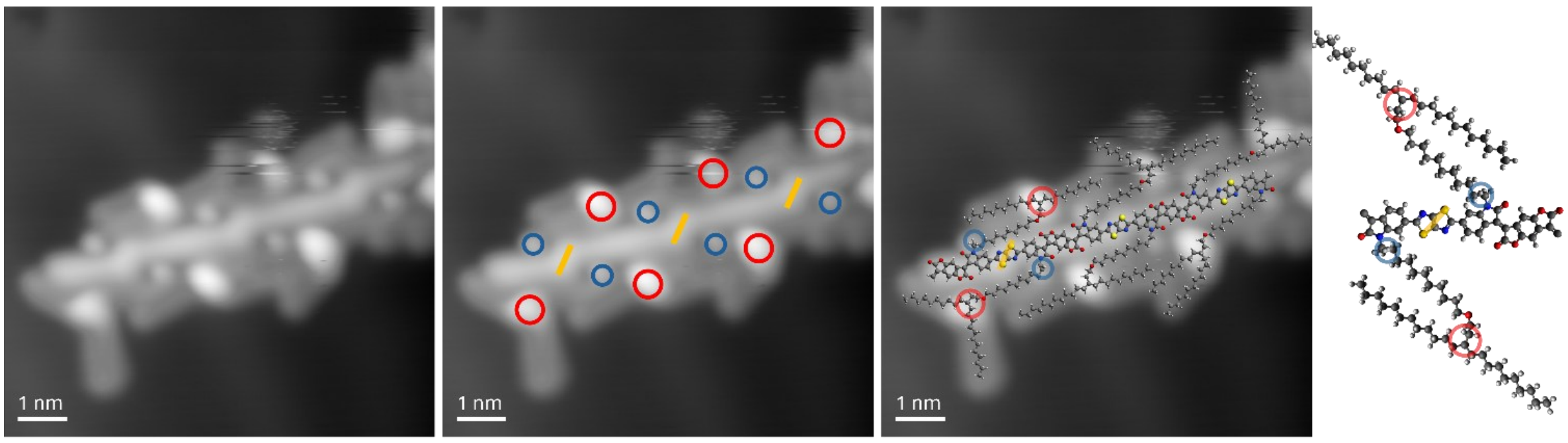

Figure S10. STM image of a defect-free isolated polymer and the method used to assign its molecular structure. Periodic features in the STM image are highlighted in different colors. Molecular repeat-unit models (shown on the right) were used as a reference to assign the structural motifs corresponding to these features. Analysis of multiple images allowed identification of the red circles as the branching points of the side chains, the smaller blue circles as the attachment points of the side chains to the backbone, and the yellow wiggles as the thiazolothiazole units.

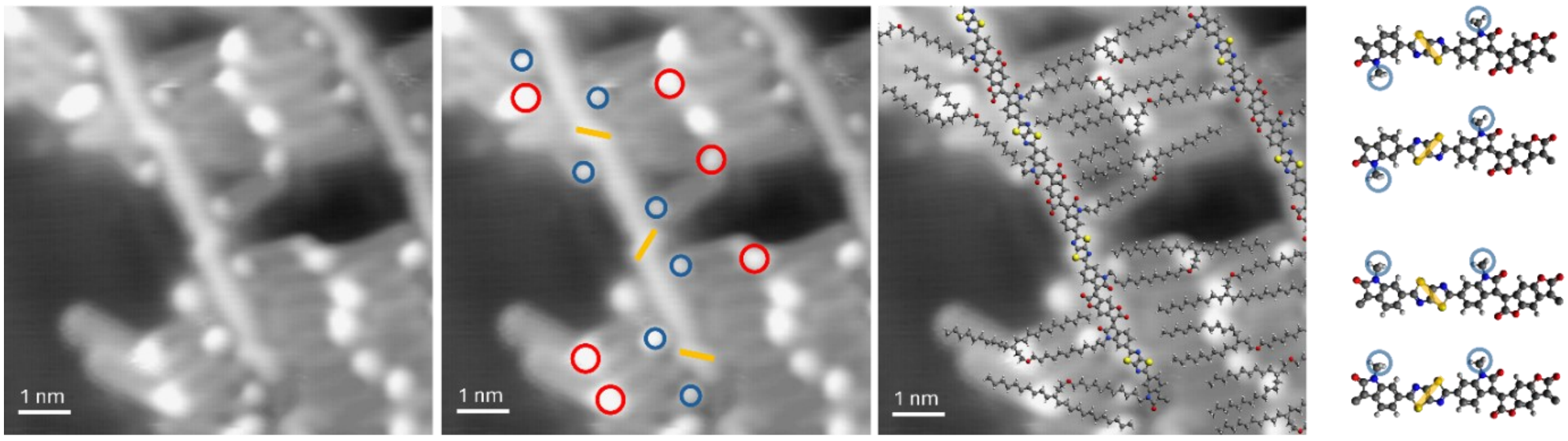

Figure S11. Procedure used to fit an STM image with a higher polymer coverage. All four possible conformations of the repeat unit (shown on the right) were considered to match the features observed in the STM image. No polymerization defects were identified; however, the conformation of the backbone affects the assembly, locally disrupting the regular interaction of the side chains.

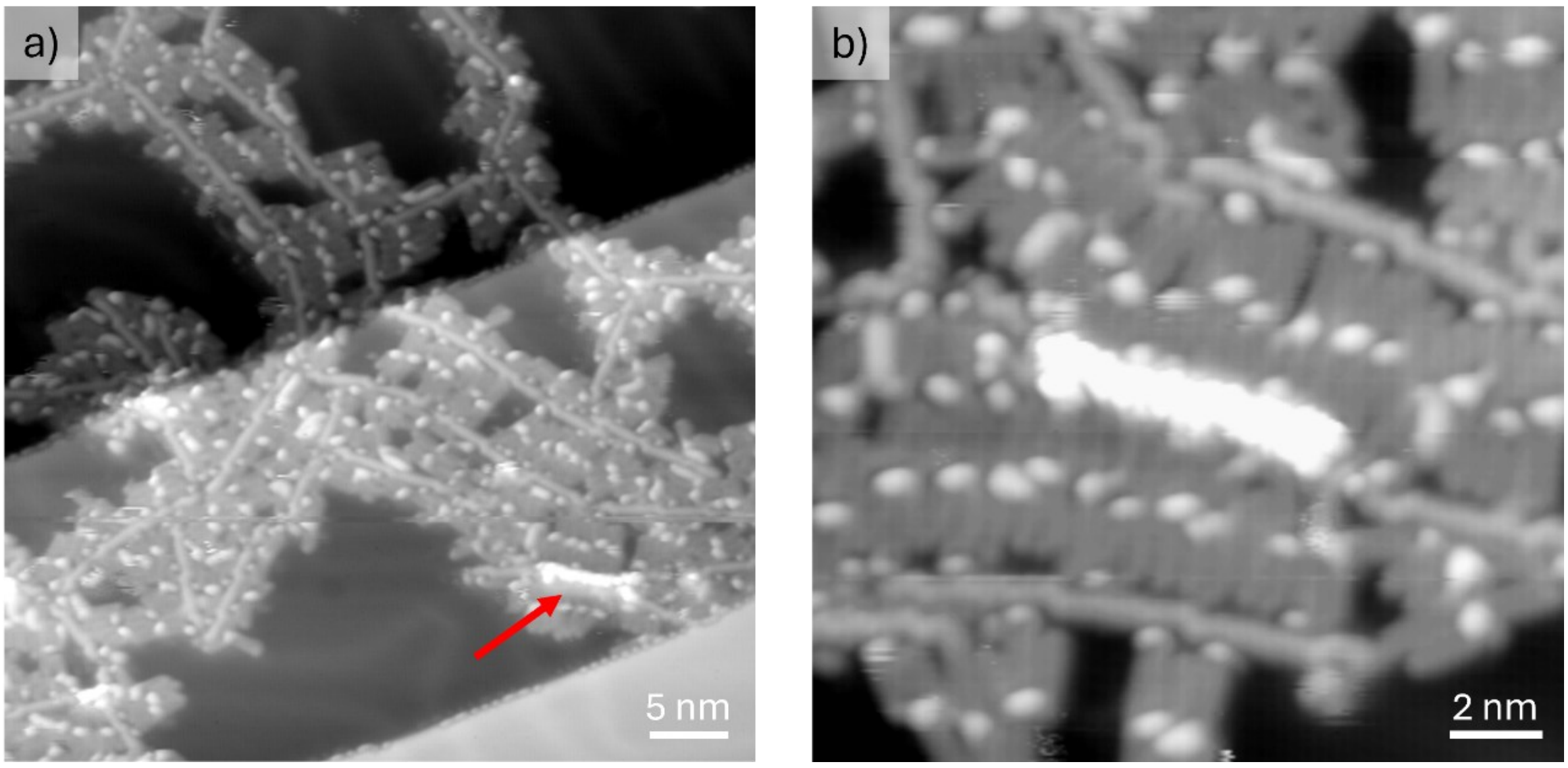

Figure S12. a) Large-scale STM image showing some polymers assembled on Au(111) terraces. The polymers exhibit a largely irregular arrangement due to the branched nature of the side chains. The arrow highlights a polymer laying on top of an underlying one, a feature frequently observed and indicative of favorable π-π stacking interactions. b) Higher resolution image of a different π-stacked polymer, in which the backbone appears brighter, while the side chains interdigitate with those of the underlying and neighboring polymers.

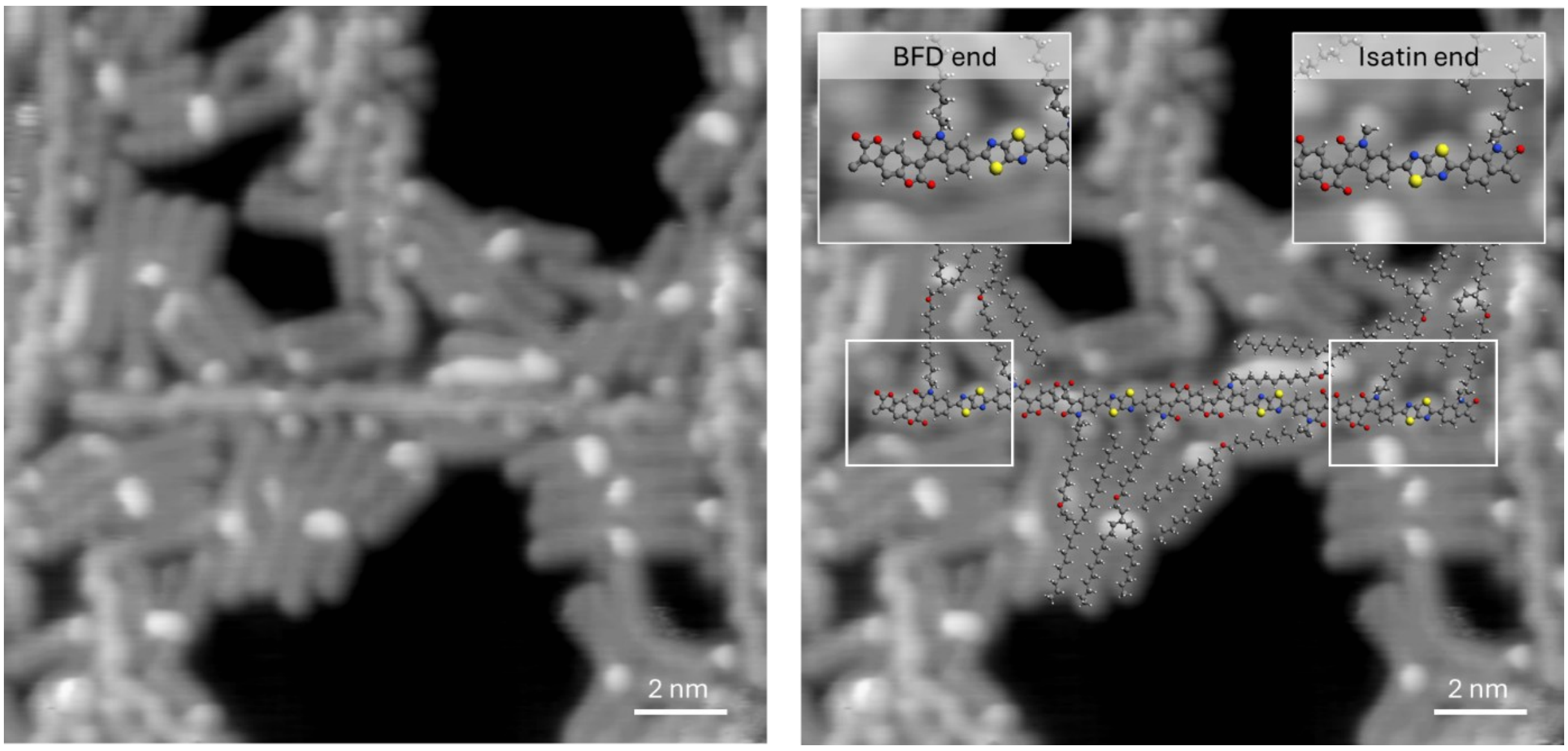

Figure S13. STM image and corresponding molecular fit of a four-repeat-unit polymer, terminating in a BDF unit on the left and an isatin unit on the right, as highlighted in the insets.

Conditions I*					
	Isatin hc	BDF hc	Kink defects	Tot defects	Tot bonds
STM counts	1	4	19	25	608
STM %	0.16	0.66	3.1	3.9	100
Yield model reaction %	<0.1	0.5	-	<5	100

Conditions I*		
End Isatin	End BDF	Tot ends
196	26	222
88.3	11.7	100

Conditions A					
	Isatin hc	BDF hc	Kink defects	Tot defects	Tot bonds
STM counts	10	11	39	60	583
STM %	1.7	1.9	6.7	10.3	100
Yield model reaction %	0.4	1.3	-	~18	100

Conditions A		
End Isatin	End BDF	Tot ends
161	13	174
92.5	7.5	100

Table S1. ESD-STM statistics of the defects and the ending monomers in **PBDFTzTz** synthesized under conditions A and I* (conditions I* are the same as conditions I but with a reaction time of 5h). In the two tables on the left, the frequencies obtained from the analysis of the STM images are compared to the relative yields (red) of the corresponding defects evaluated by integrating the NMR spectra for the small molecule model reactions (Table 1). Note that the frequency of isatin hc are compared with the relative yield of **15**, while the frequencies of BDF hc with the sum of the relative yields of **13** and **14**.

4. NMR Spectra

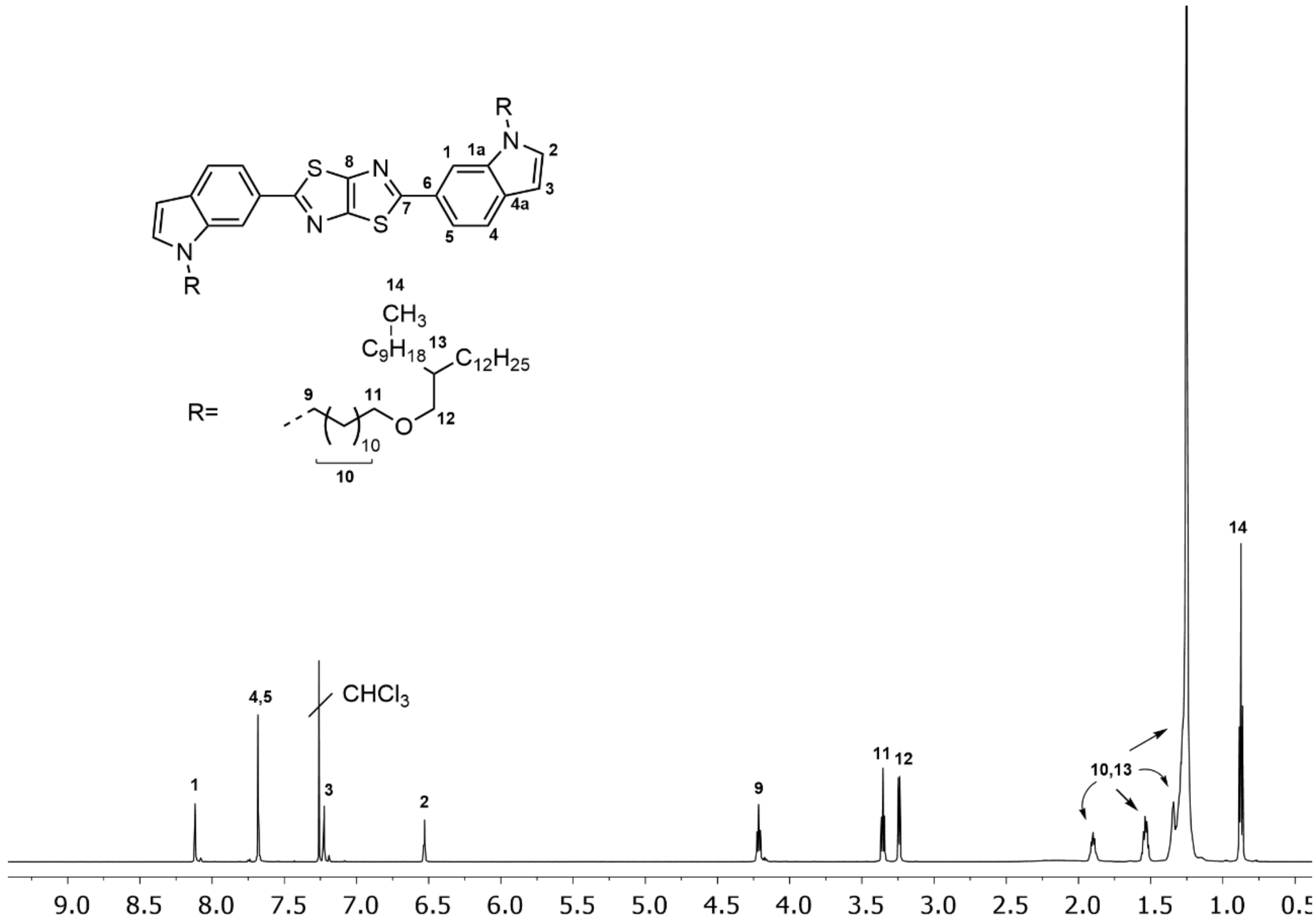

Figure S14. ^{1}H-NMR spectrum of (**3**) in CDCl$_3$ at 298K.

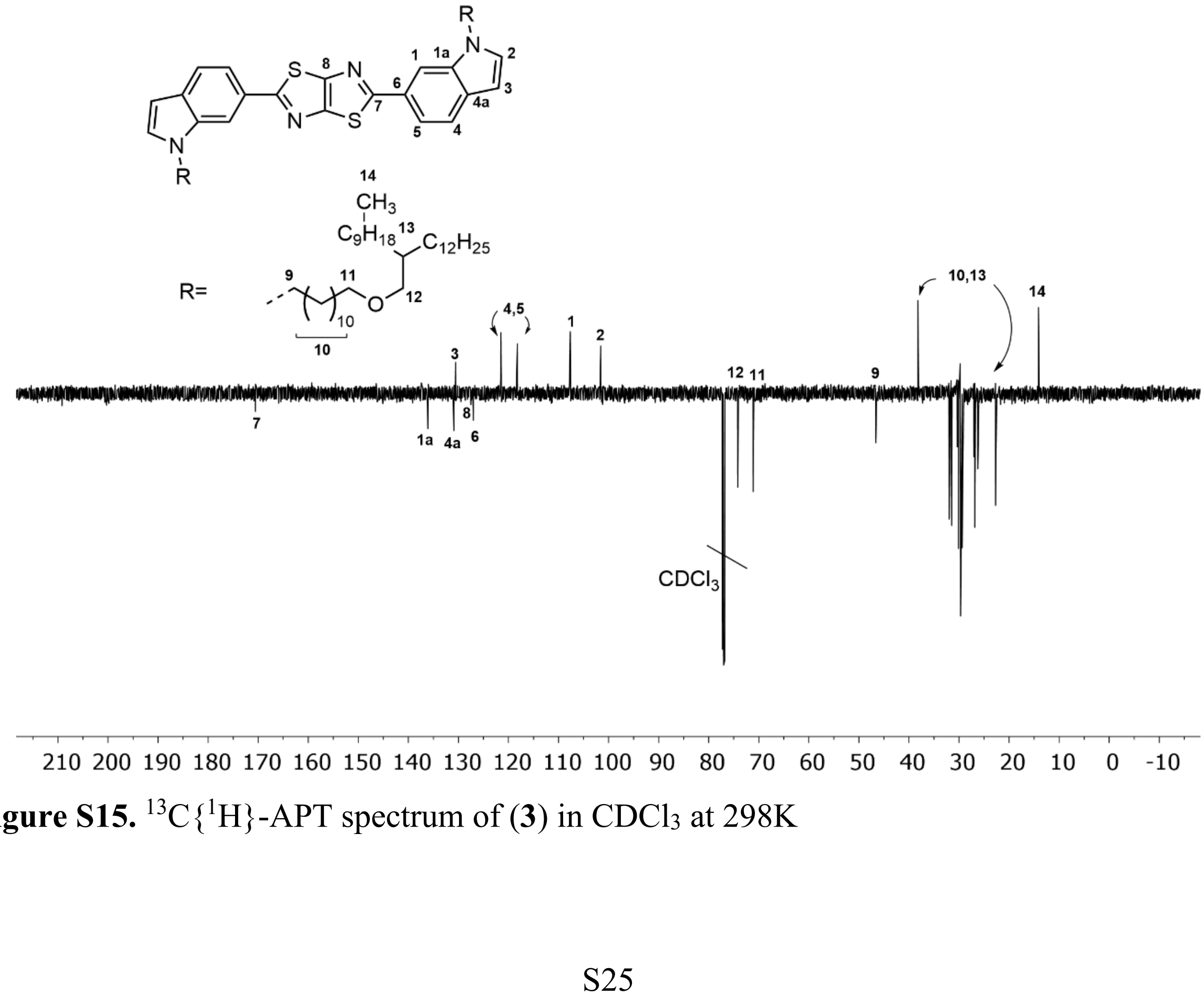

Figure S15. ^{13}C{^{1}H}-APT spectrum of (**3**) in CDCl$_3$ at 298K

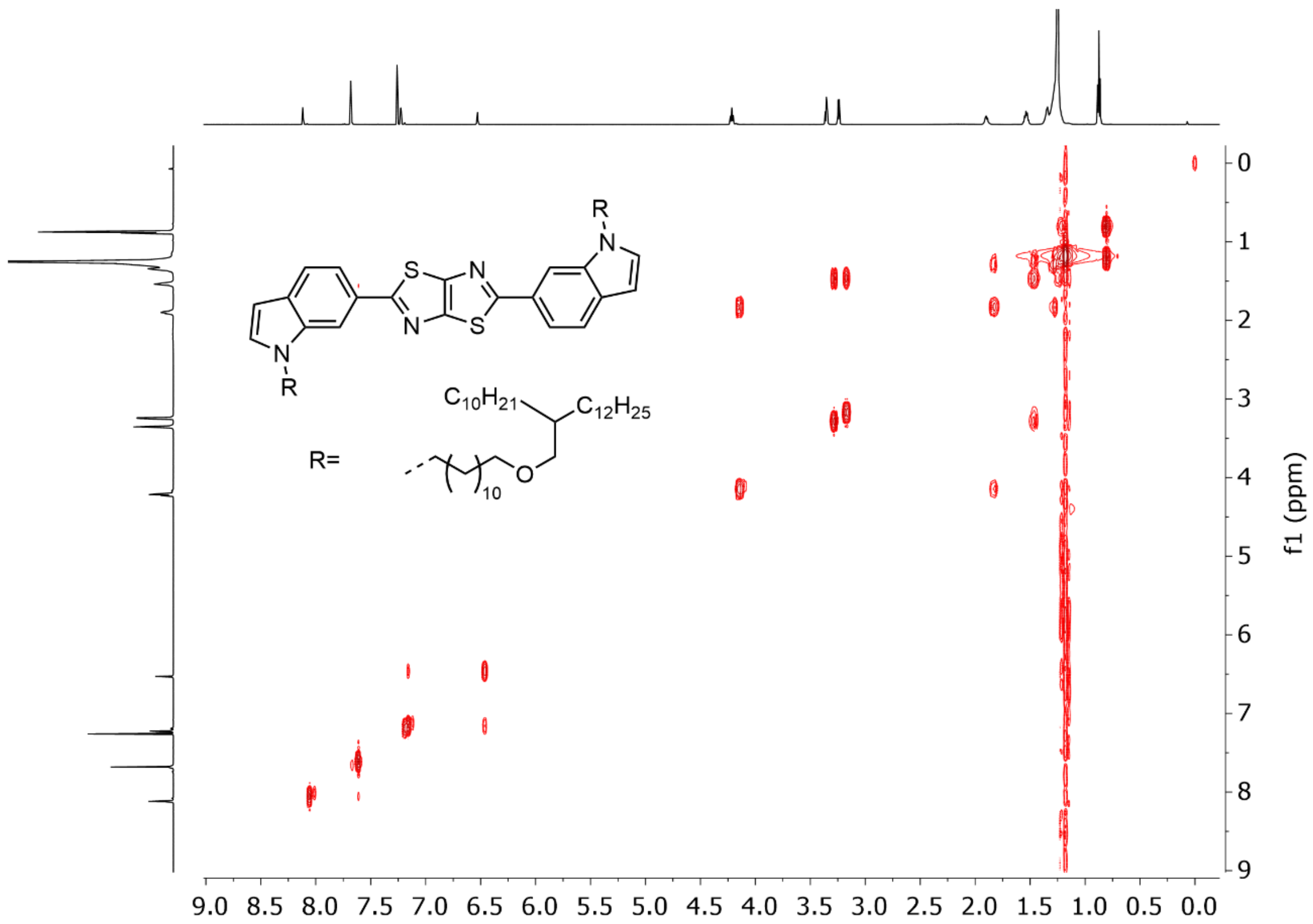

Figure S16. ^{1}H-^{1}H COSY spectrum of (**3**) in $CDCl_3$ at 298K.

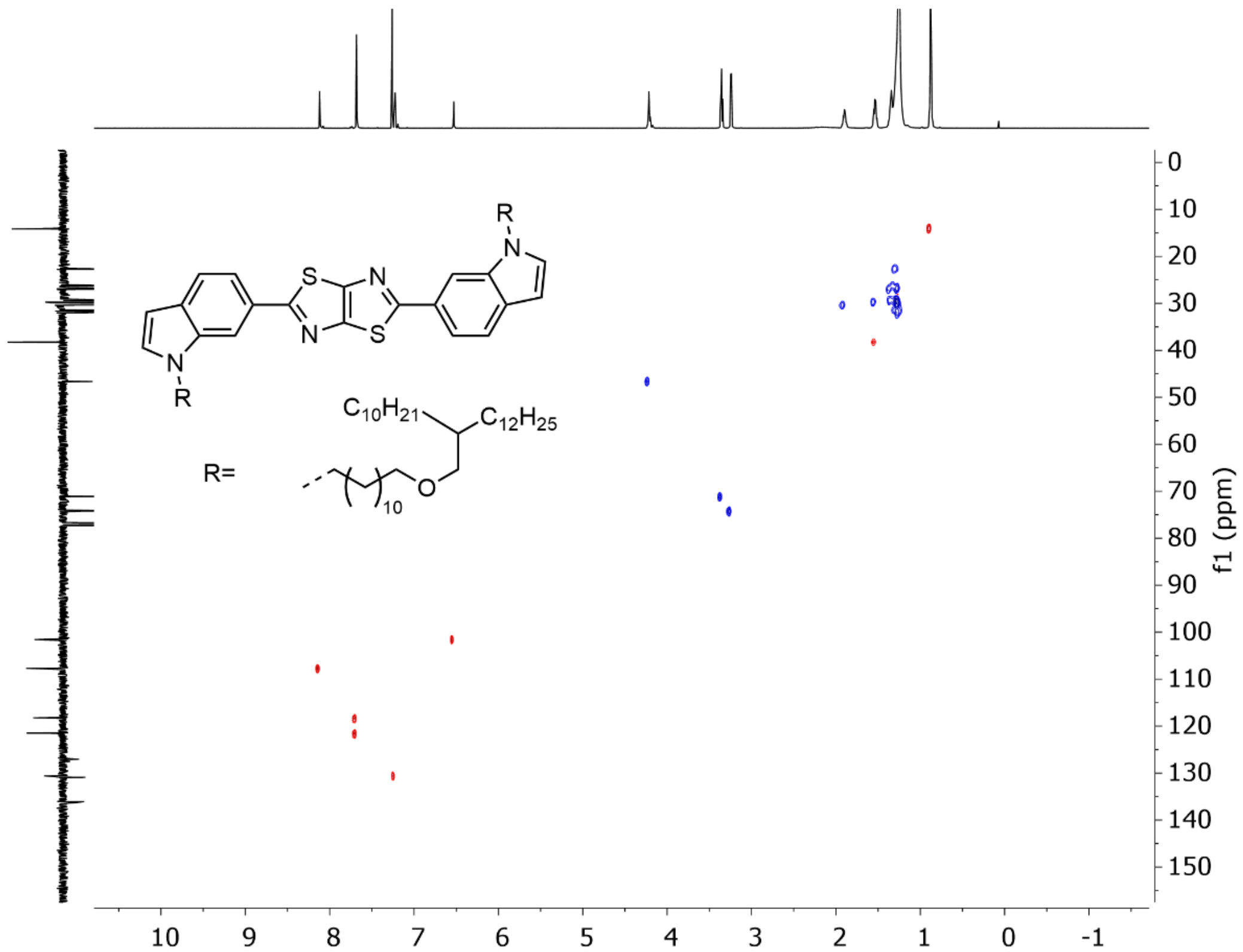

Figure S17. ^{1}H-^{13}C HSQC spectrum of (**3**) in $CDCl_3$ at 298K.

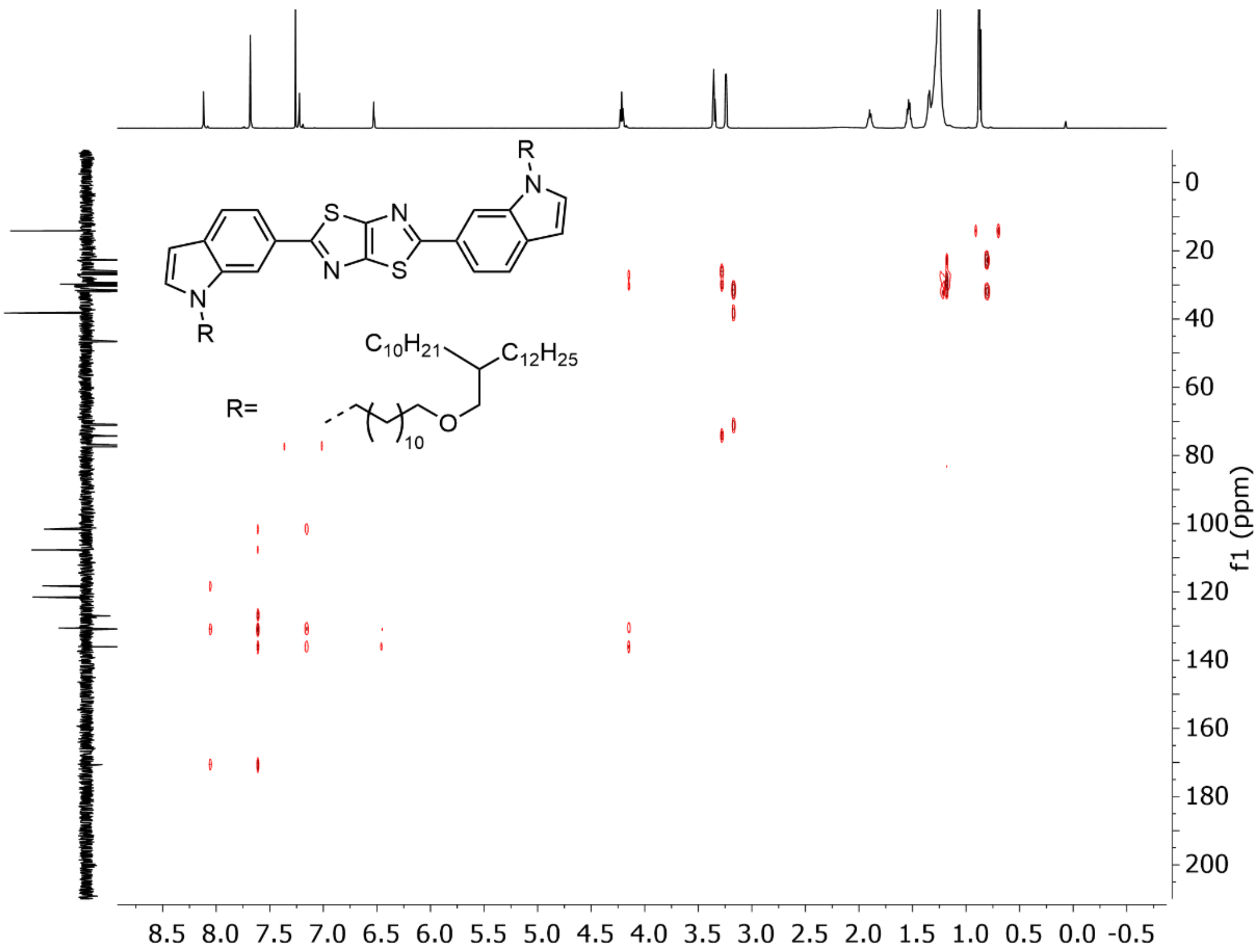

Figure S18. ^{1}H-^{13}C HMBC spectrum of (**3**) in $CDCl_3$ at 298K.

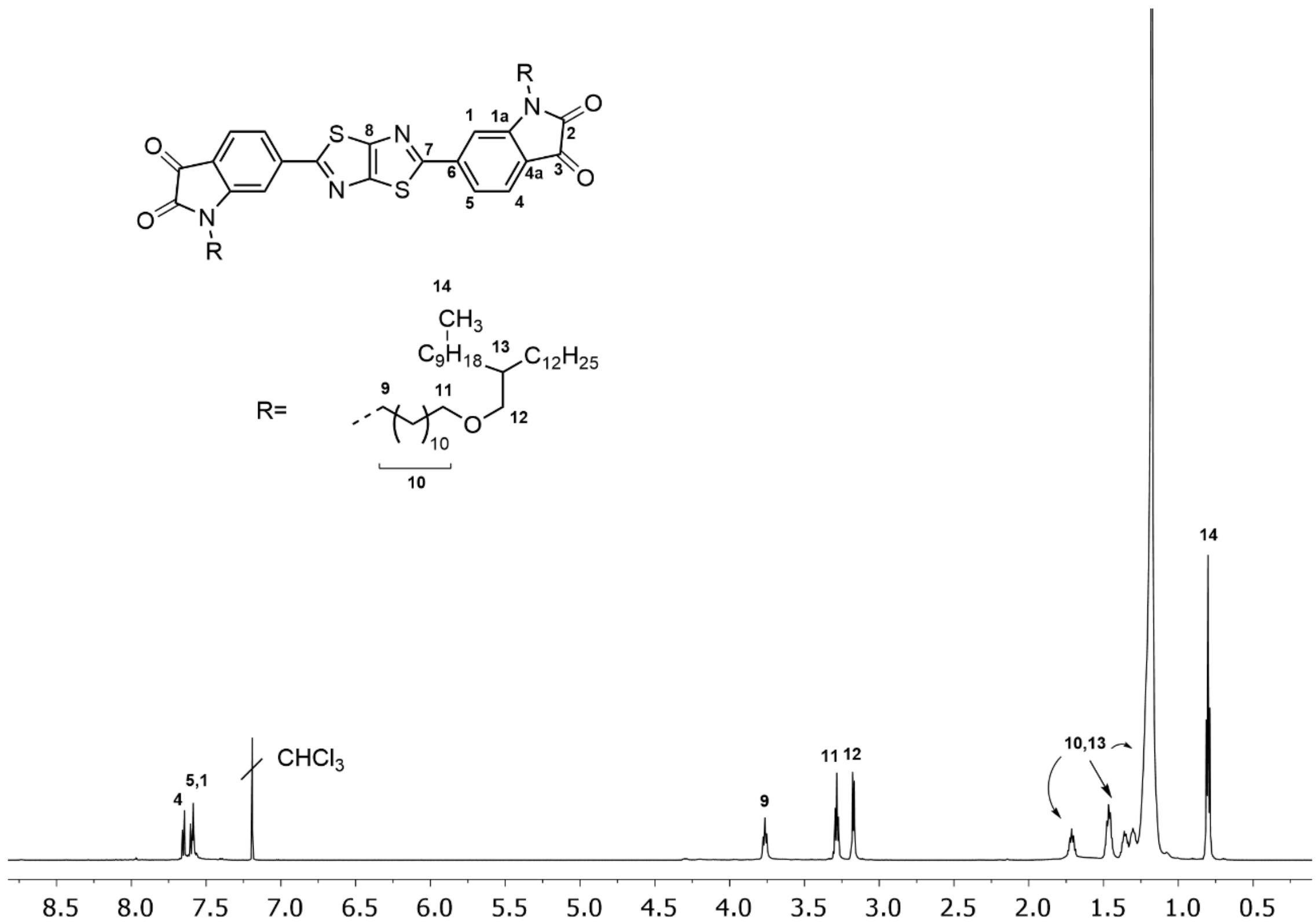

Figure S19. ^{1}H-NMR spectrum of (**4**) in $CDCl_3$ at 298K.

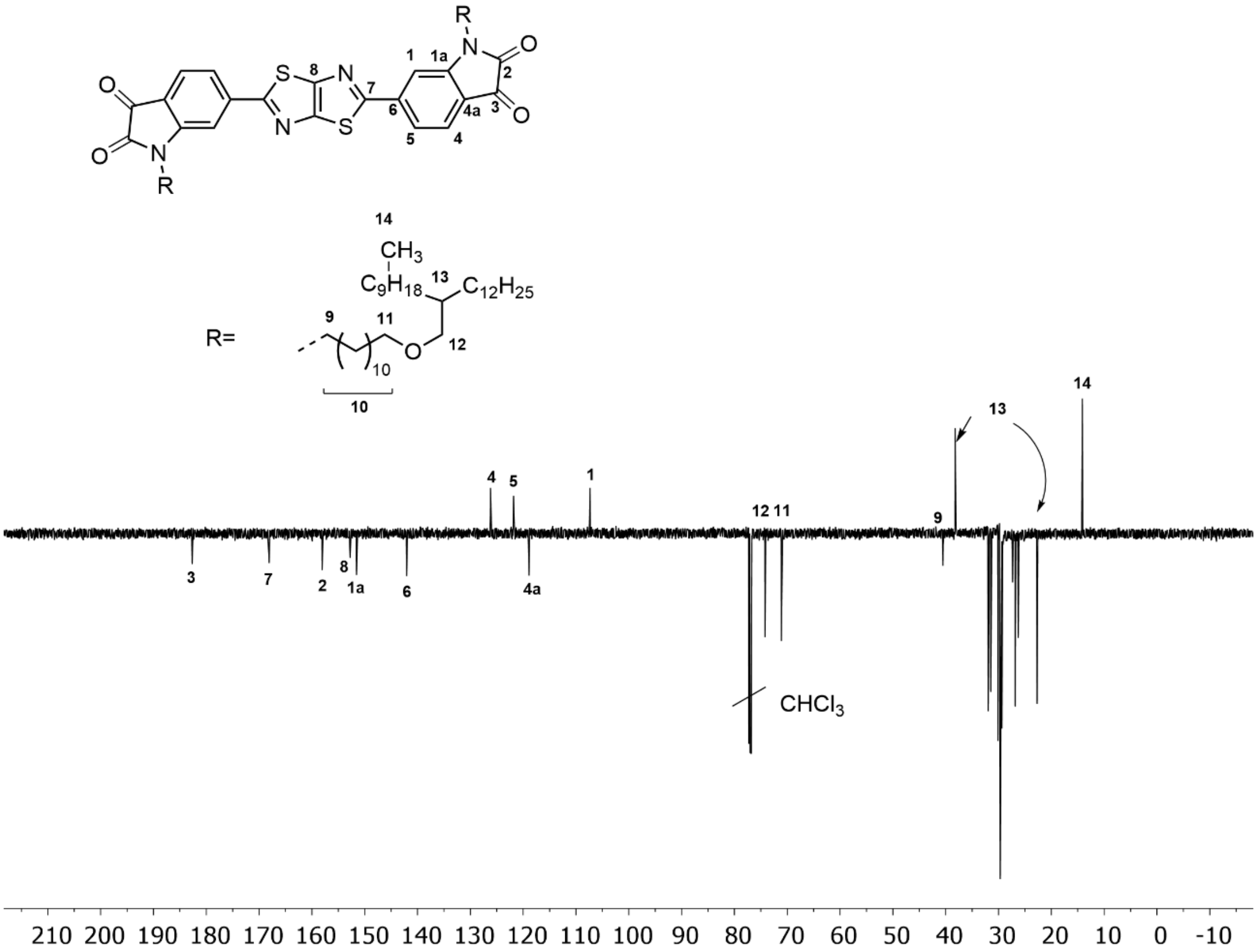

Figure S20. $^{13}C\{^{1}H\}$-APT spectrum of (**4**) in $CDCl_3$ at 298K

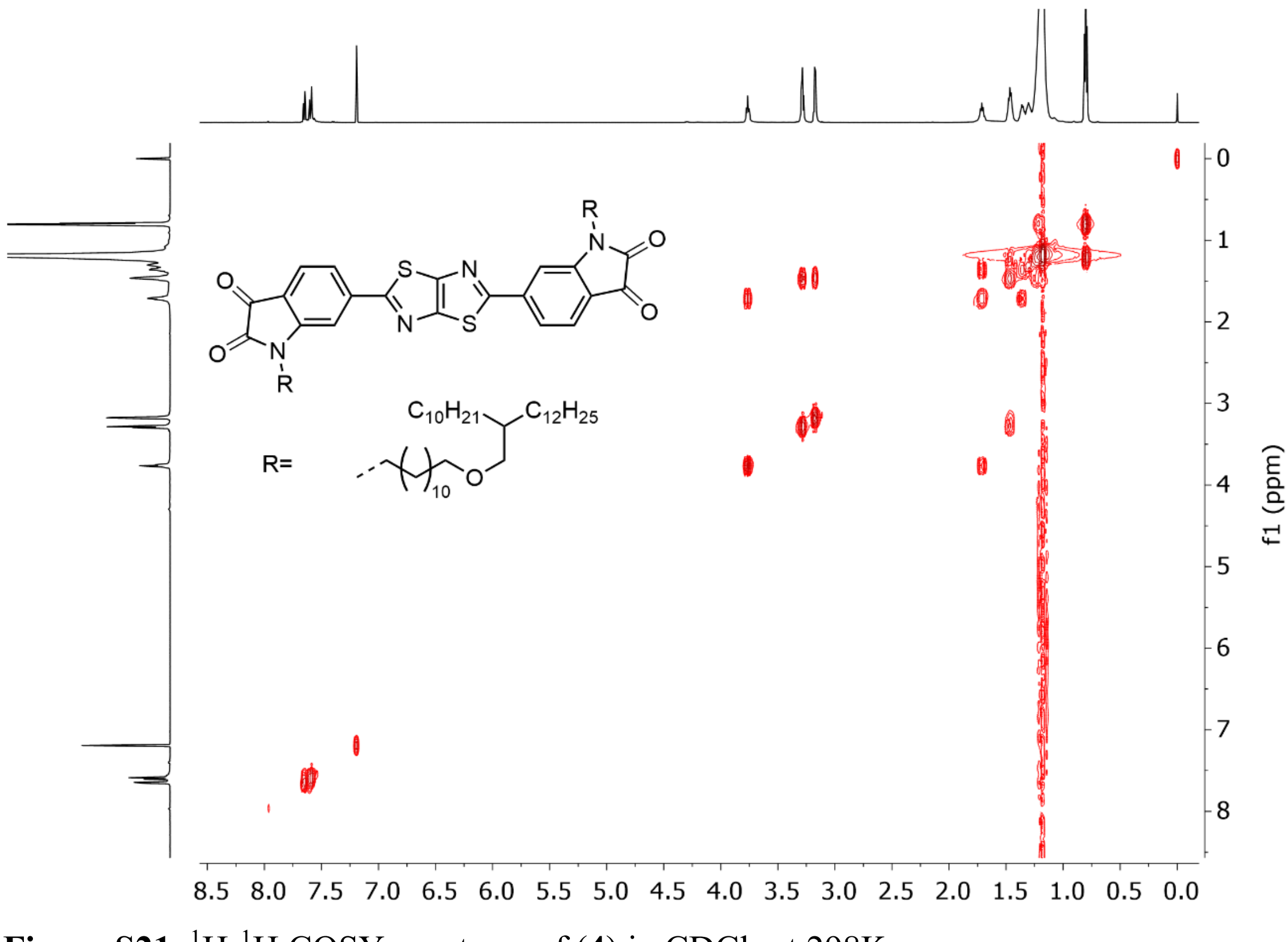

Figure S21. ^{1}H-^{1}H COSY spectrum of (**4**) in $CDCl_3$ at 298K.

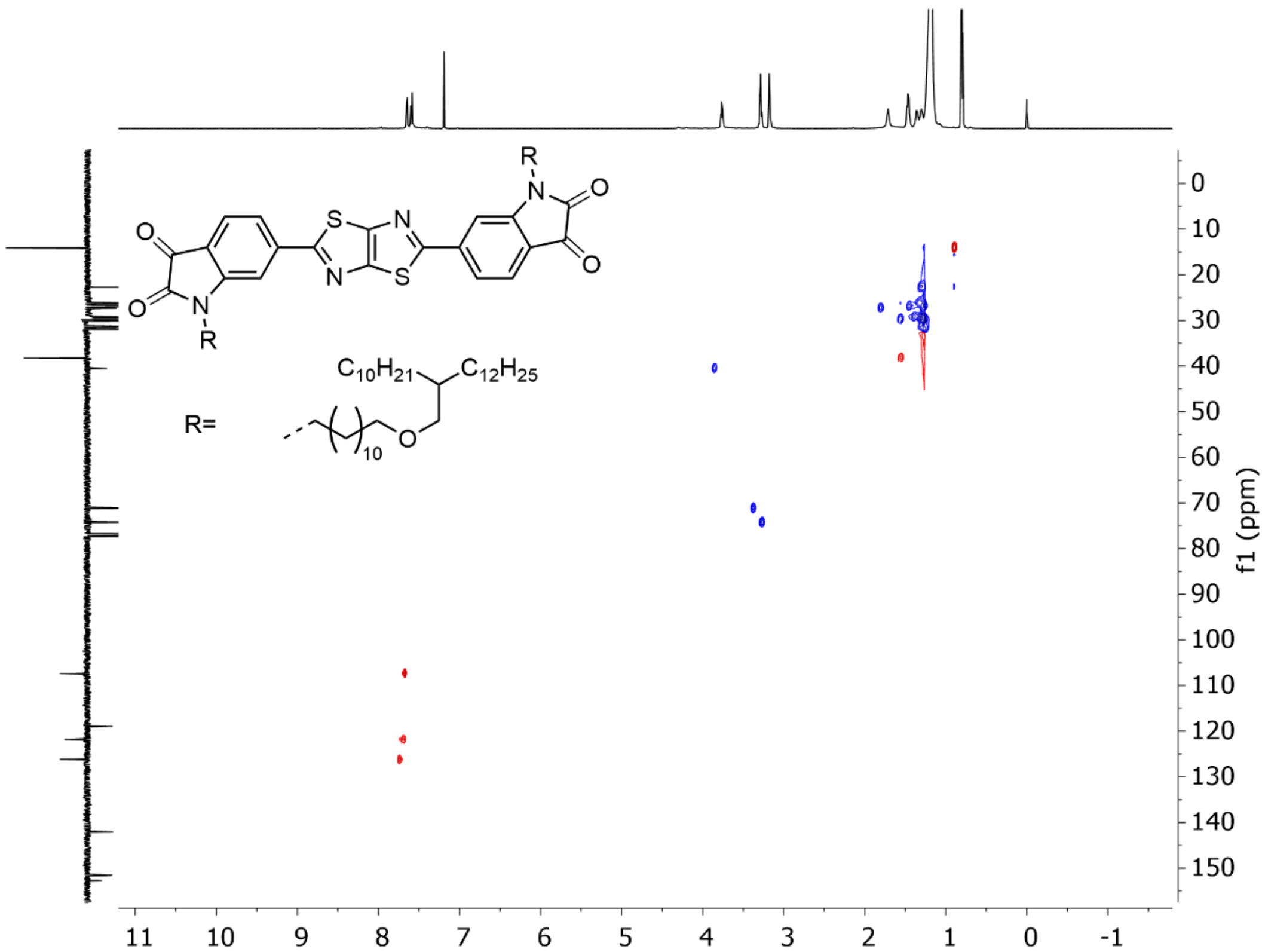

Figure S22. ^{1}H-^{13}C HSQC spectrum of (**4**) in $CDCl_3$ at 298K.

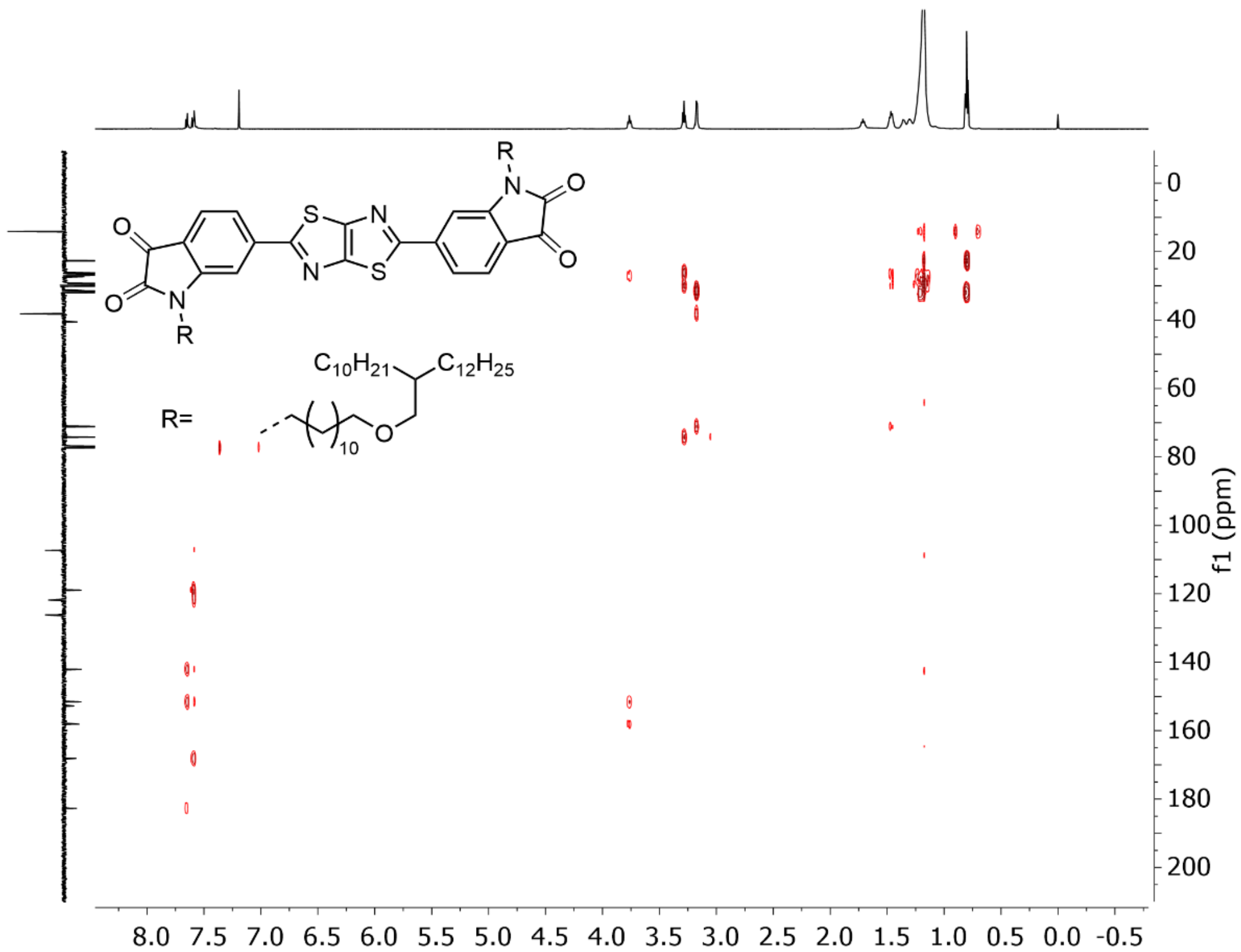

Figure S23. ^{1}H-^{13}C HMBC spectrum of (**4**) in $CDCl_3$ at 298K.

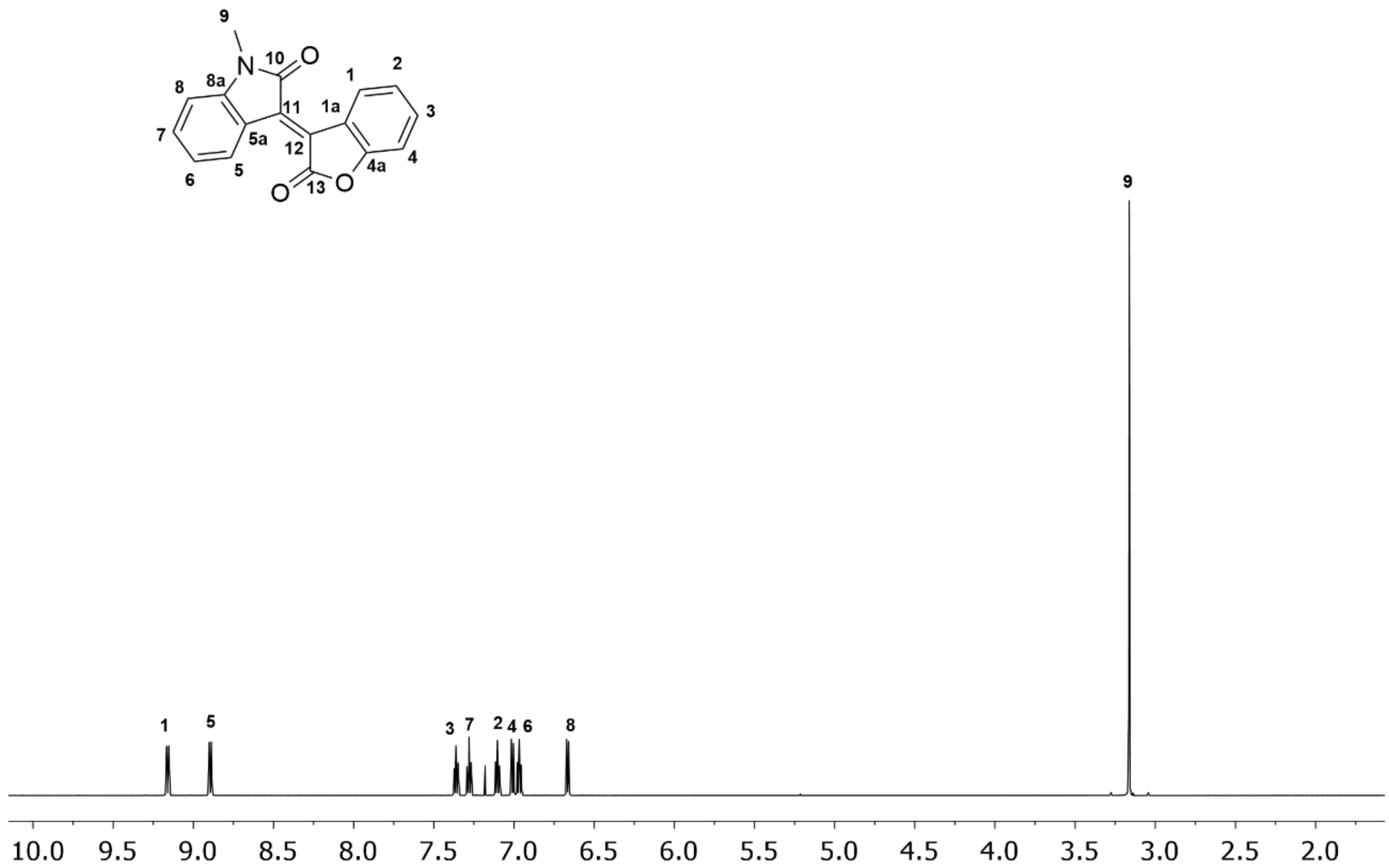

Figure S24. ^{1}H-NMR spectrum of (**11**) in $CDCl_3$ at 298K.

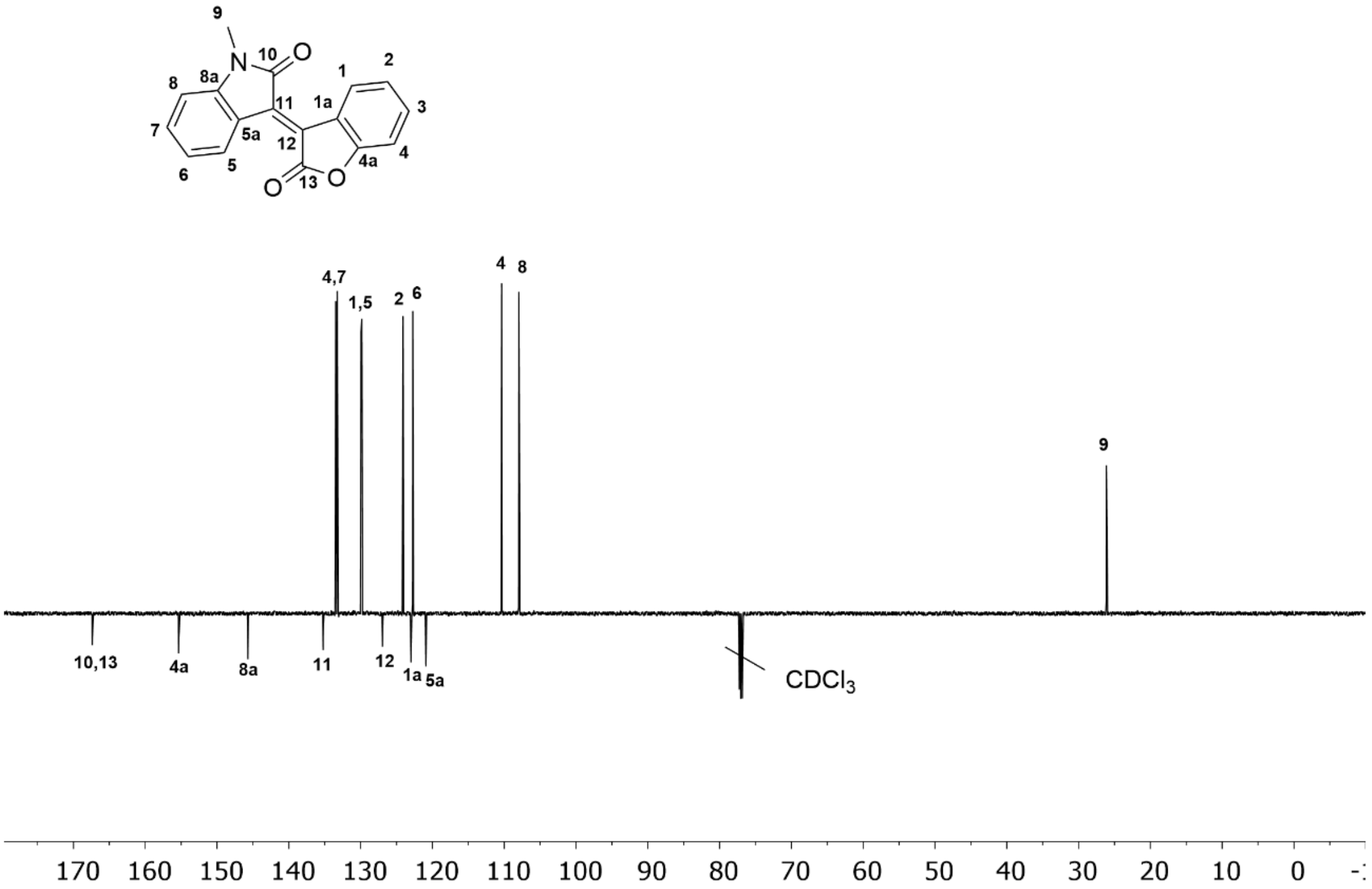

Figure S25. $^{13}C\{^{1}H\}$-APT spectrum of (**11**) in $CDCl_3$ at 298K

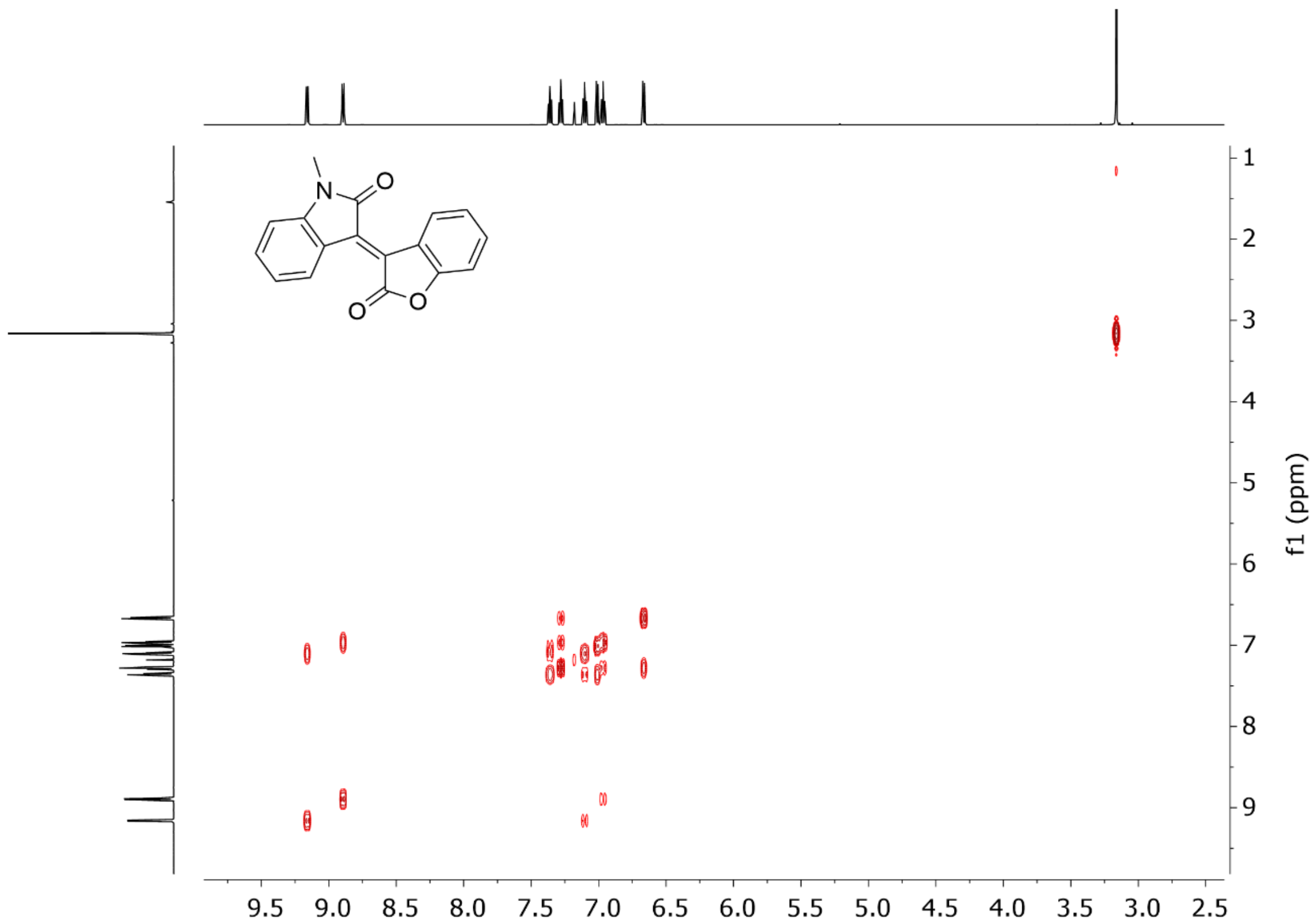

Figure S26. ¹H-¹H COSY spectrum of (**11**) in $CDCl_3$ at 298K.

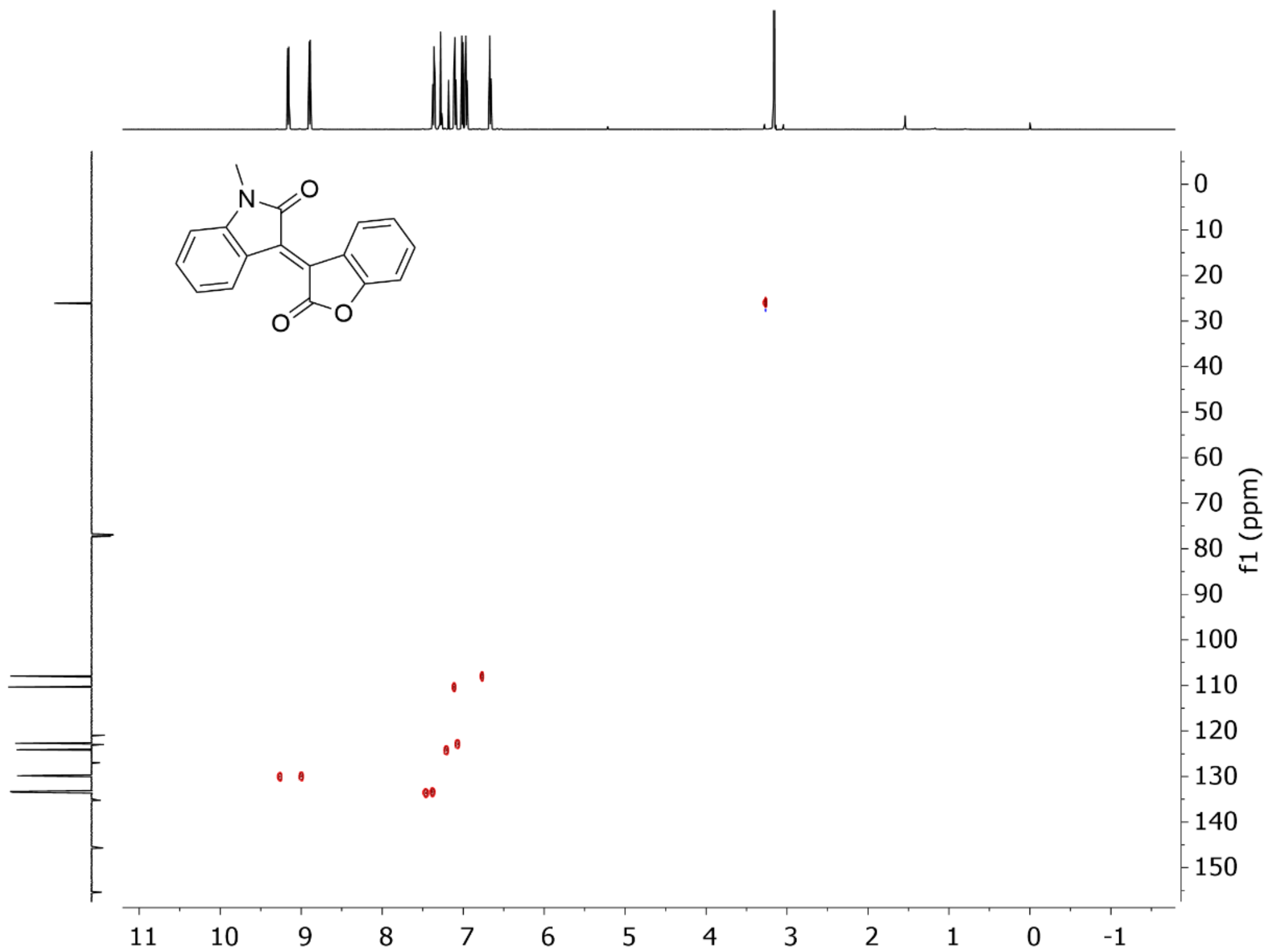

Figure S27. ¹H-¹³C HSQC spectrum of (**11**) in $CDCl_3$ at 298K.

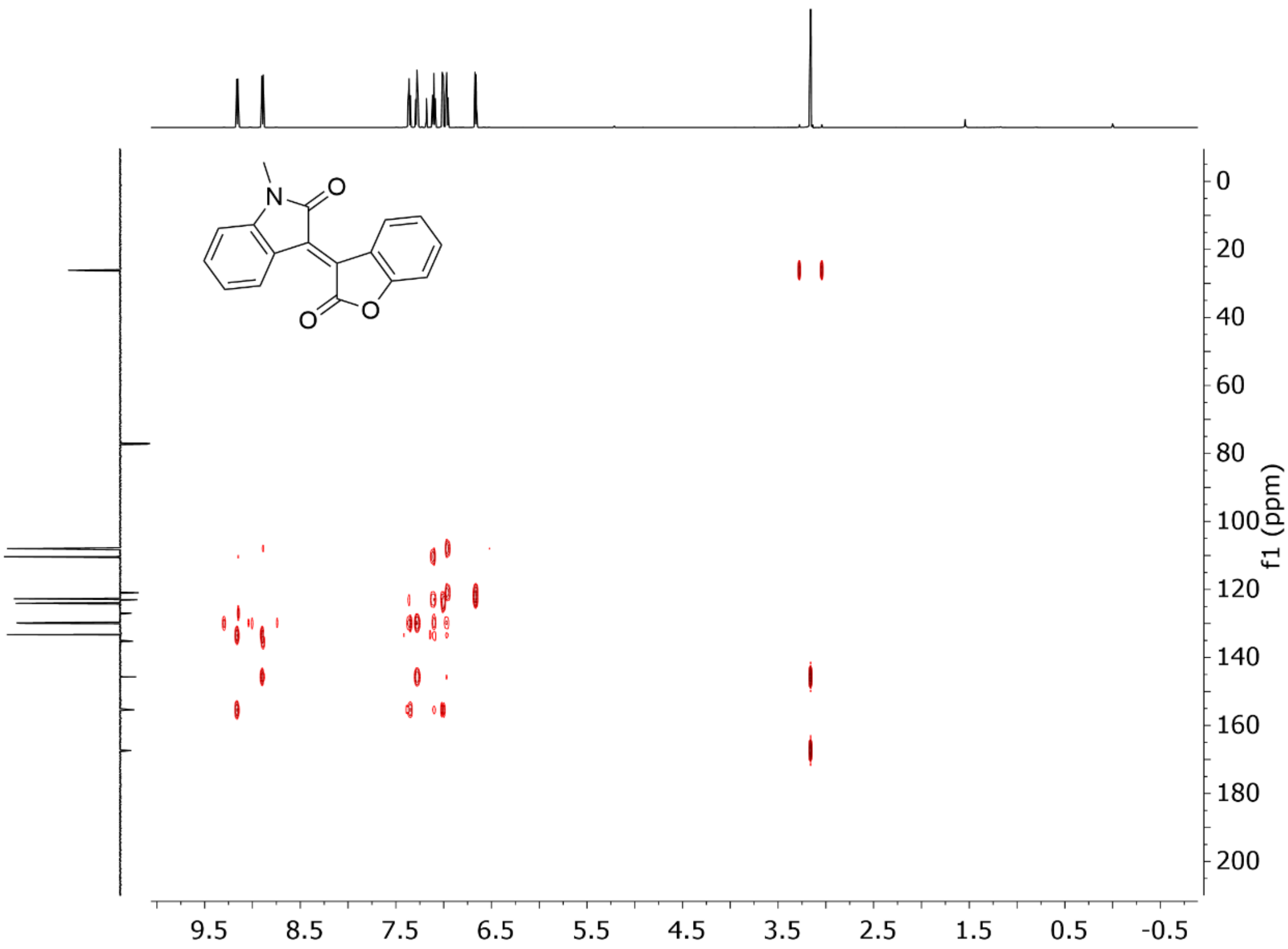

Figure S28. ^{1}H-^{13}C HMBC spectrum of (**11**) in $CDCl_3$ at 298K.

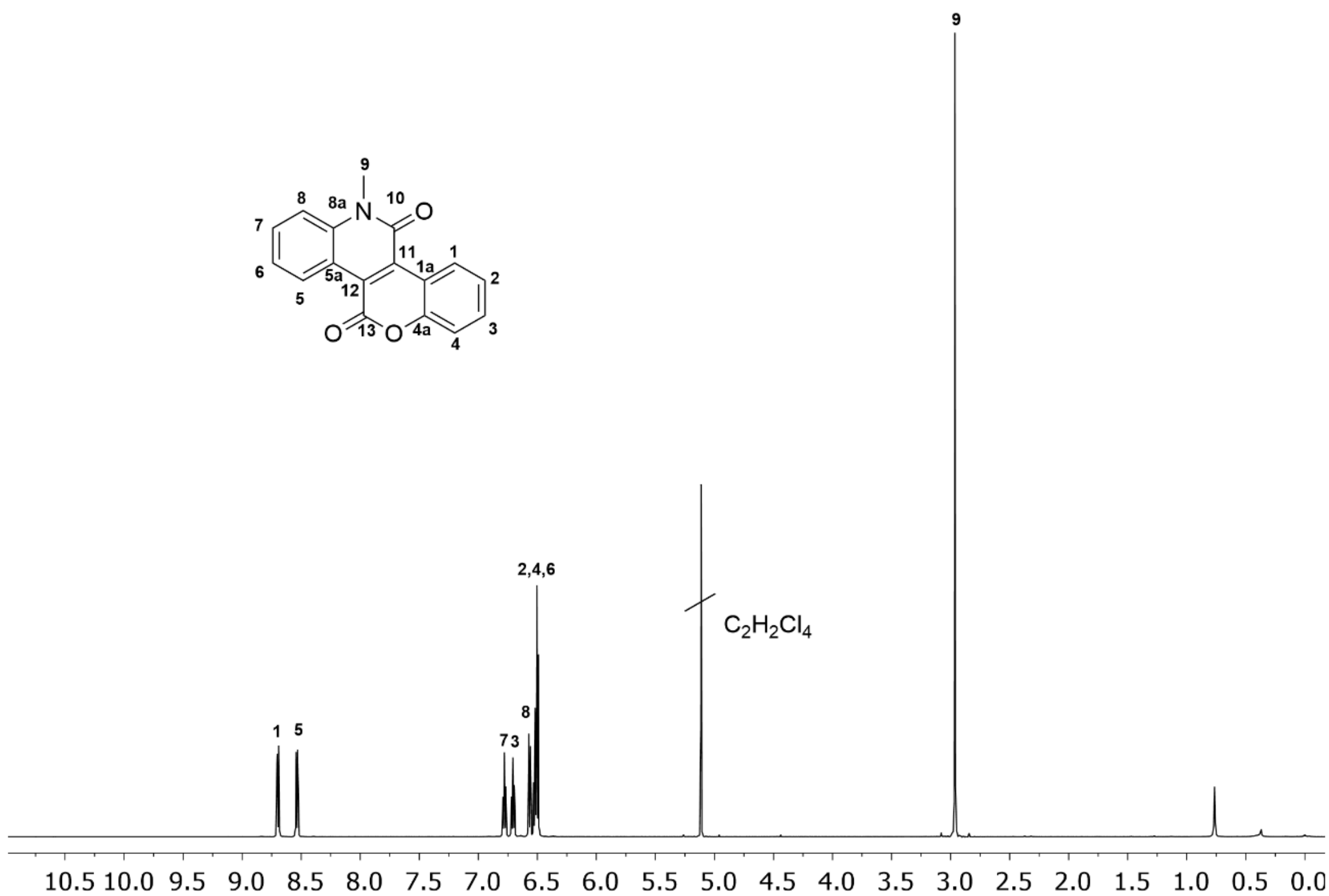

Figure S29. ^{1}H-NMR spectrum of (**12**) in $CD_2D_2Cl_2$ at 298K.

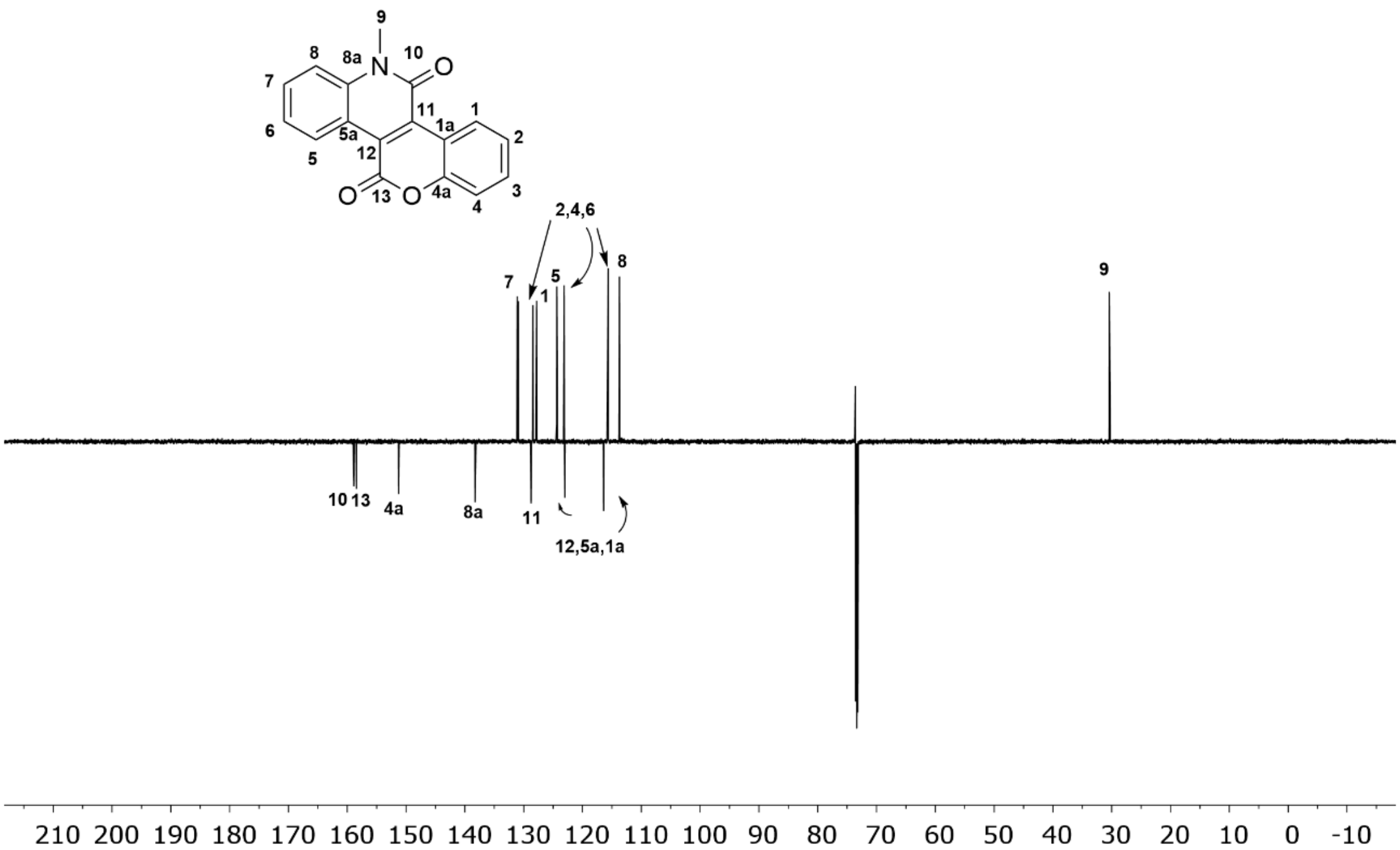

Figure S30. $^{13}C\{^{1}H\}$-APT spectrum of (**12**) in $CD_2D_2Cl_2$ at 298K.

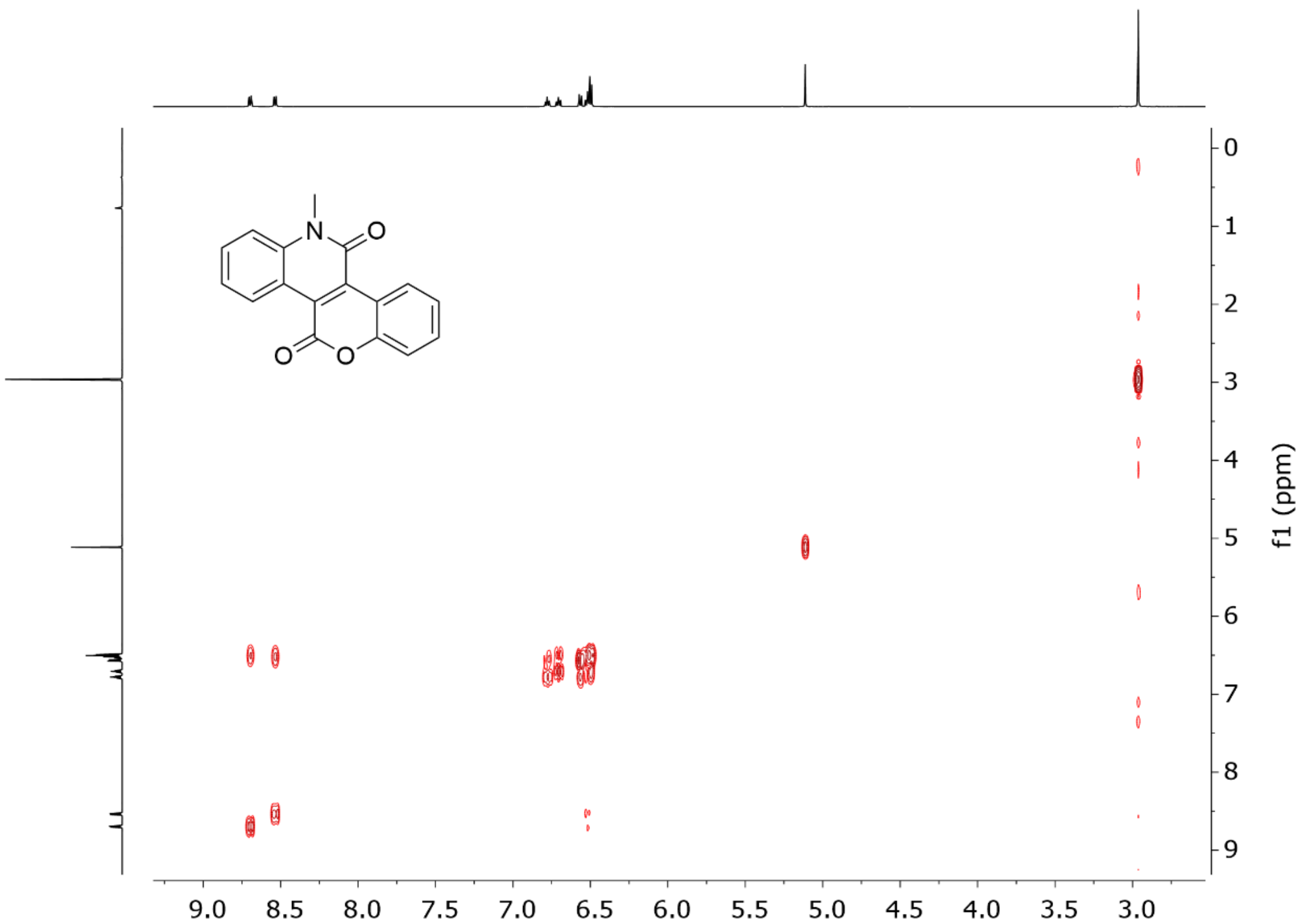

Figure S31. ^{1}H-^{1}H COSY spectrum of (**12**) in $CD_2D_2Cl_2$ at 298K.

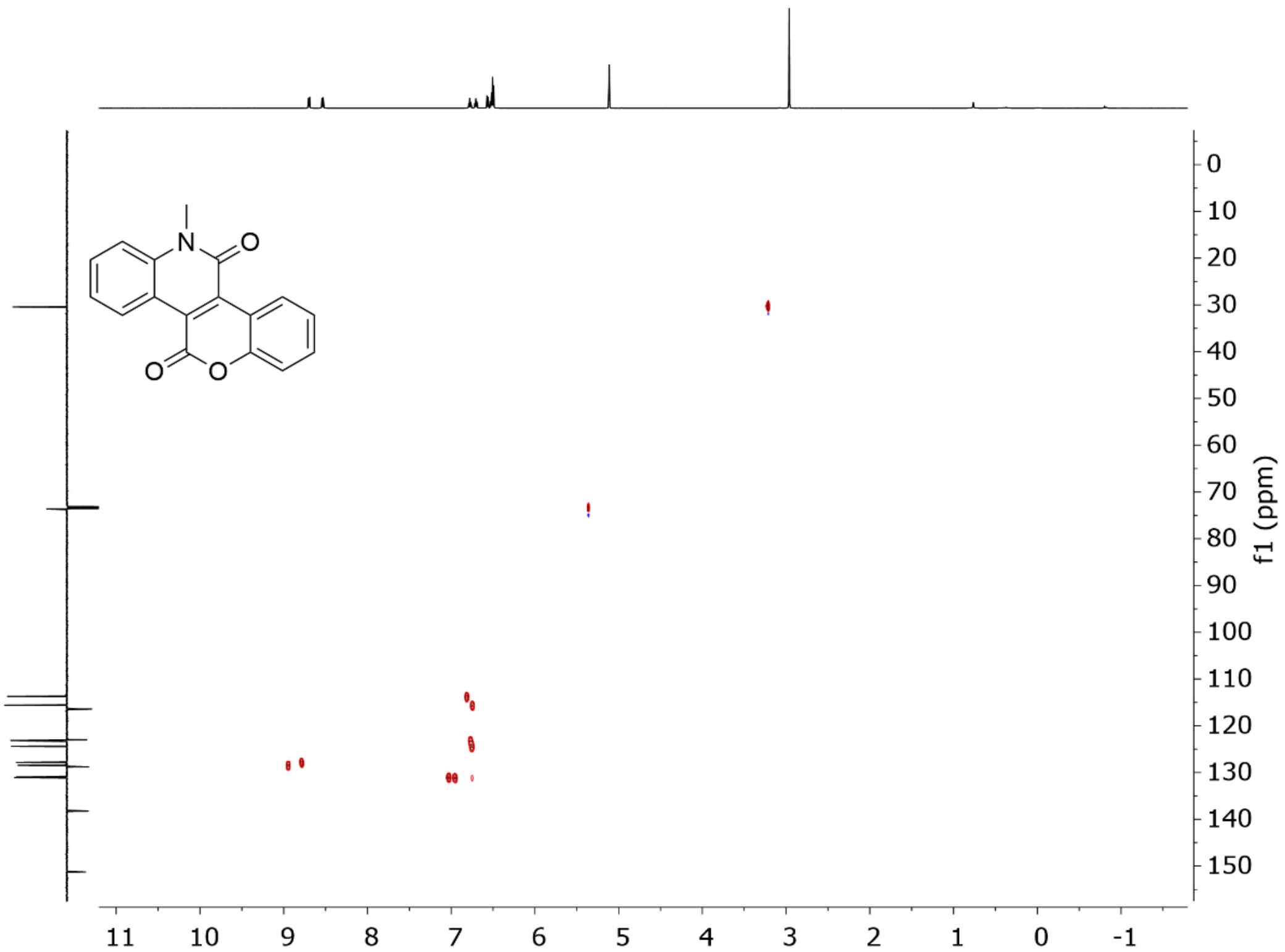

Figure S32. ^{1}H-^{13}C HSQC spectrum of (**12**) in $CD_2D_2Cl_2$ at 298K.

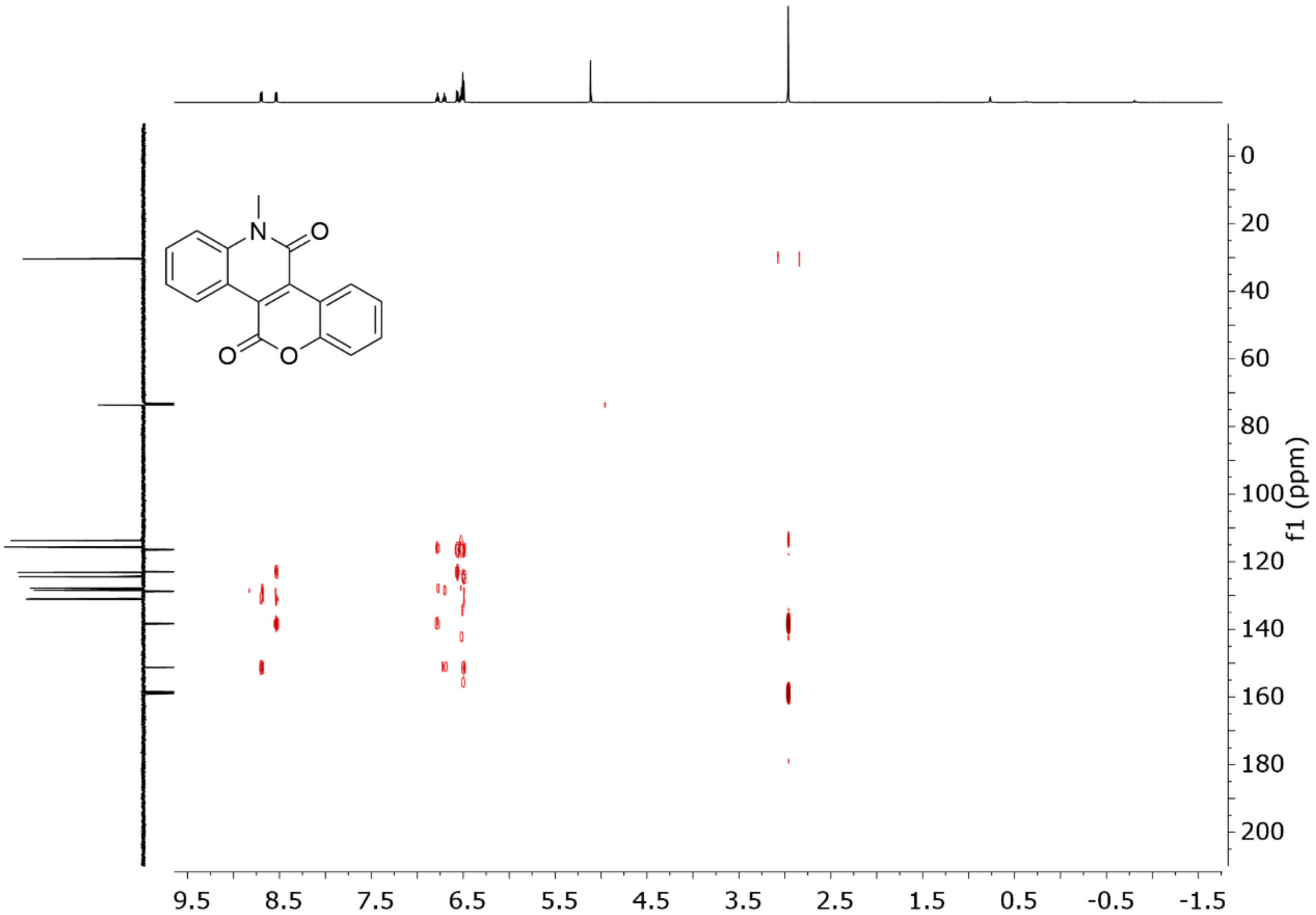

Figure S33. ^{1}H-^{13}C HMBC spectrum of (**12**) in $CD_2D_2Cl_2$ at 298K.

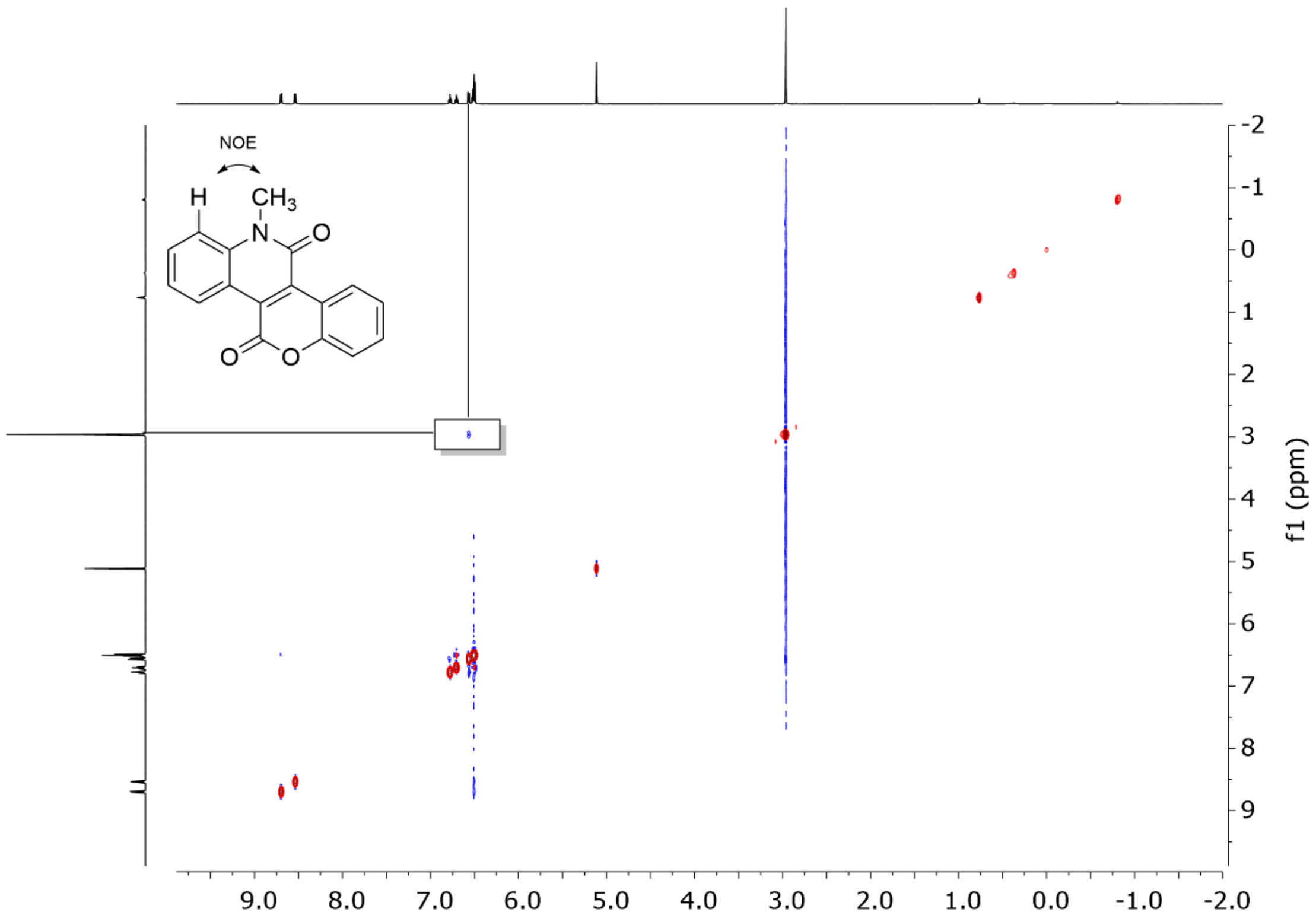

Figure S34. ^{1}H-^{1}H NOESY spectrum of (**12**) in $C_2D_2Cl_4$ at 298K.

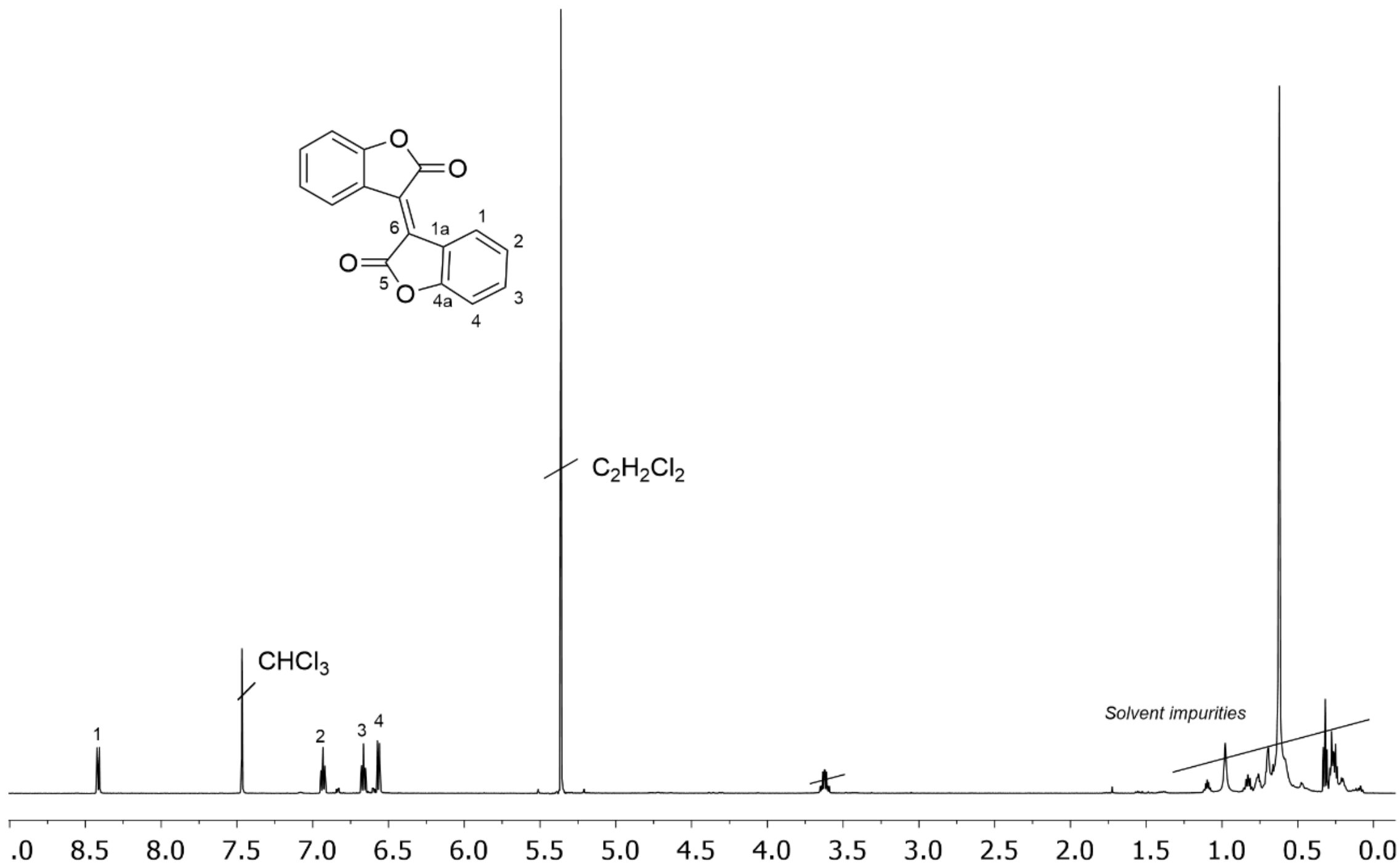

Figure S35. ^{1}H-NMR spectrum of (**13**) in $C_2D_2Cl_4$ at 298K.

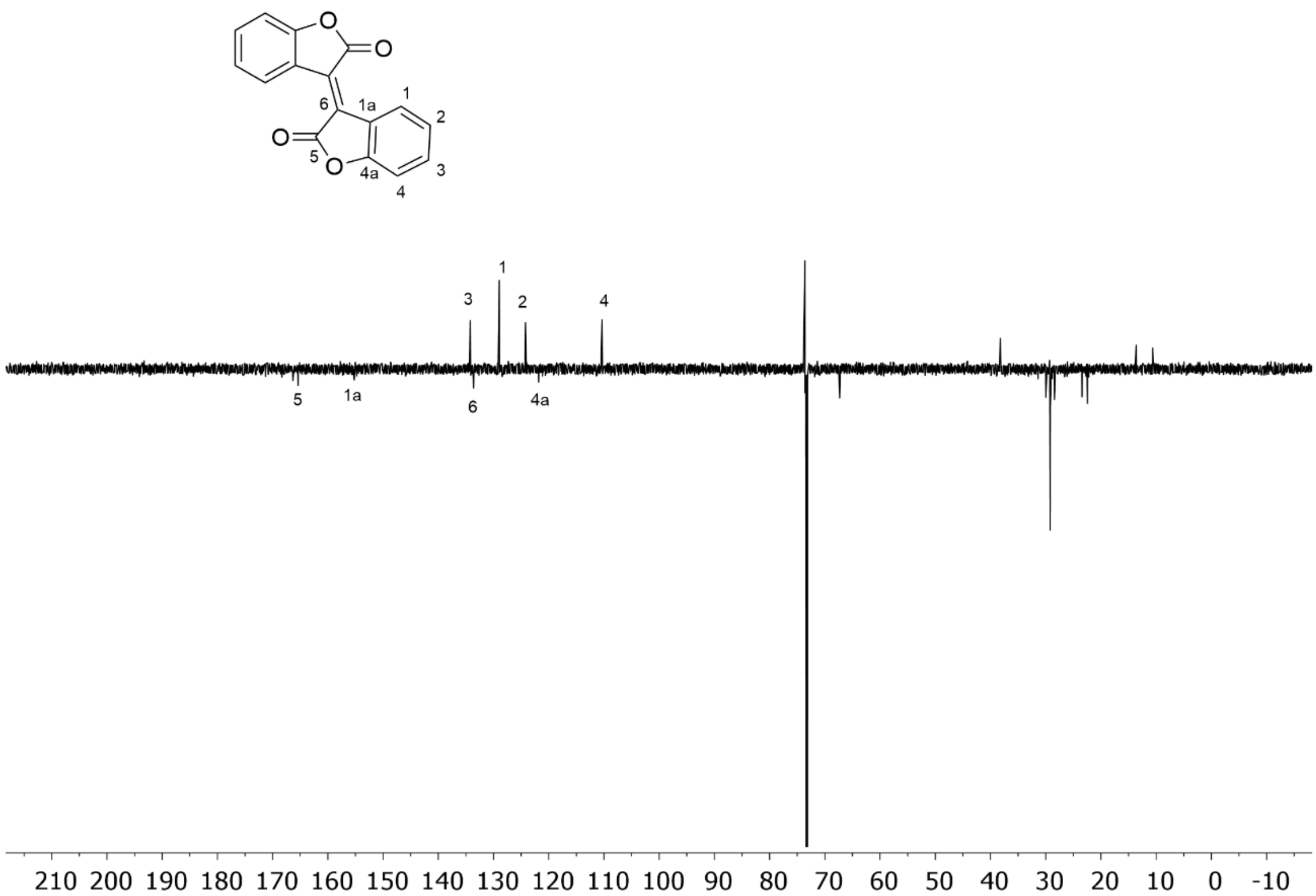

Figure S36. $^{13}C\{^{1}H\}$-APT spectrum of (**13**) in $CD_2D_2Cl_2$ at 298K.

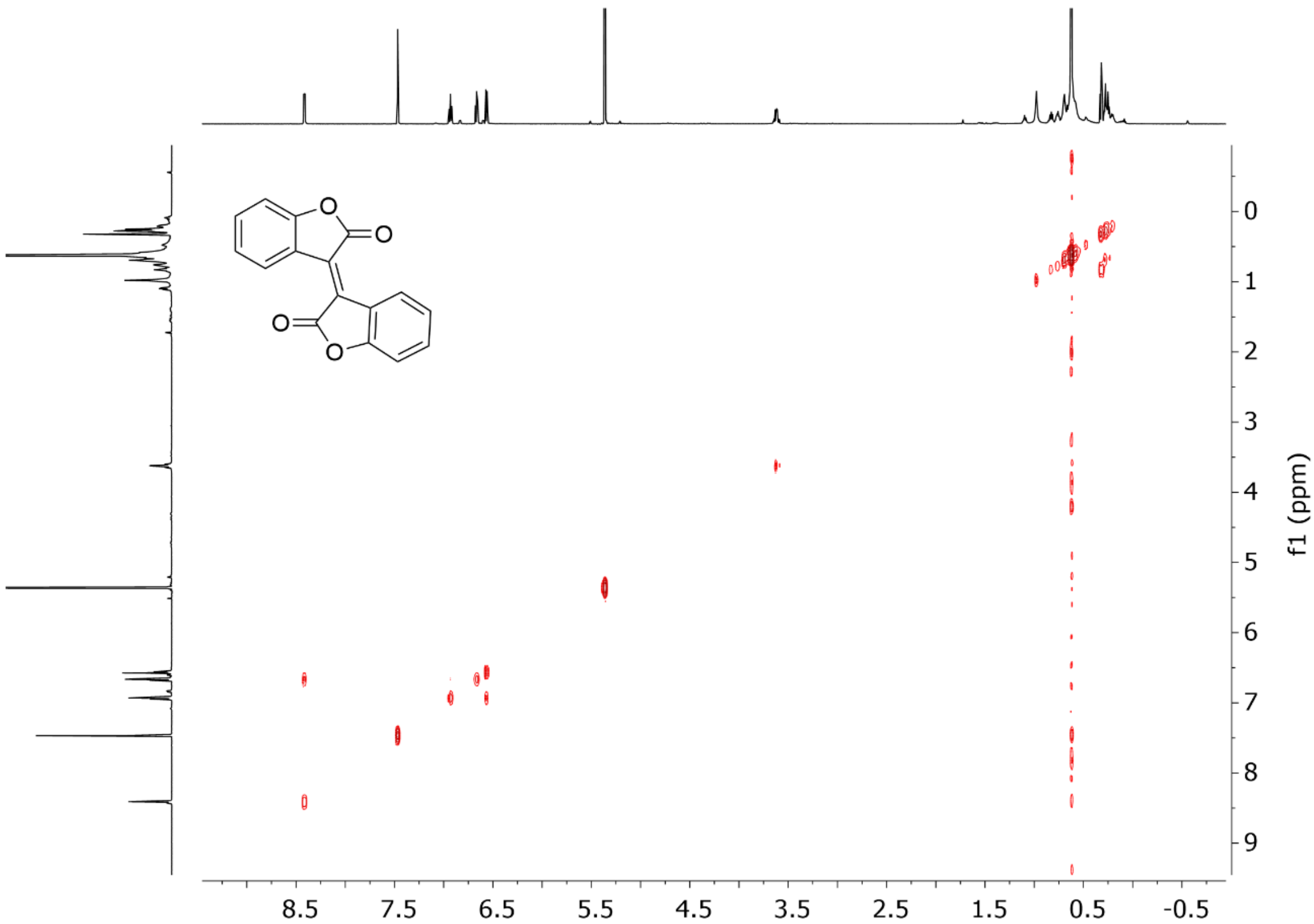

Figure S37. ^{1}H-^{1}H COSY spectrum of (**13**) in $CD_2D_2Cl_2$ at 298K.

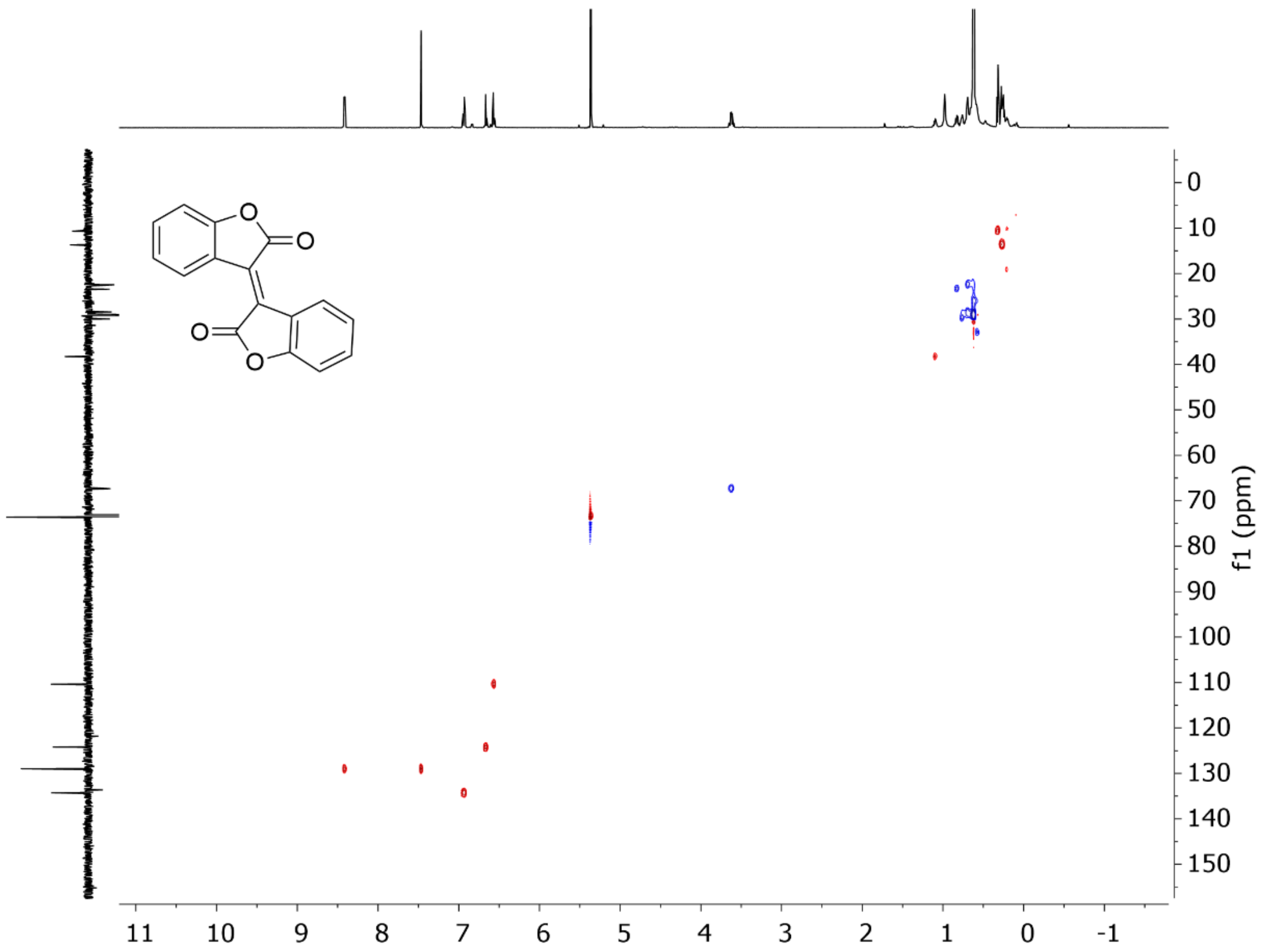

Figure S38. ^{1}H-^{13}C HSQC spectrum of (**13**) in $CD_2D_2Cl_2$ at 298K.

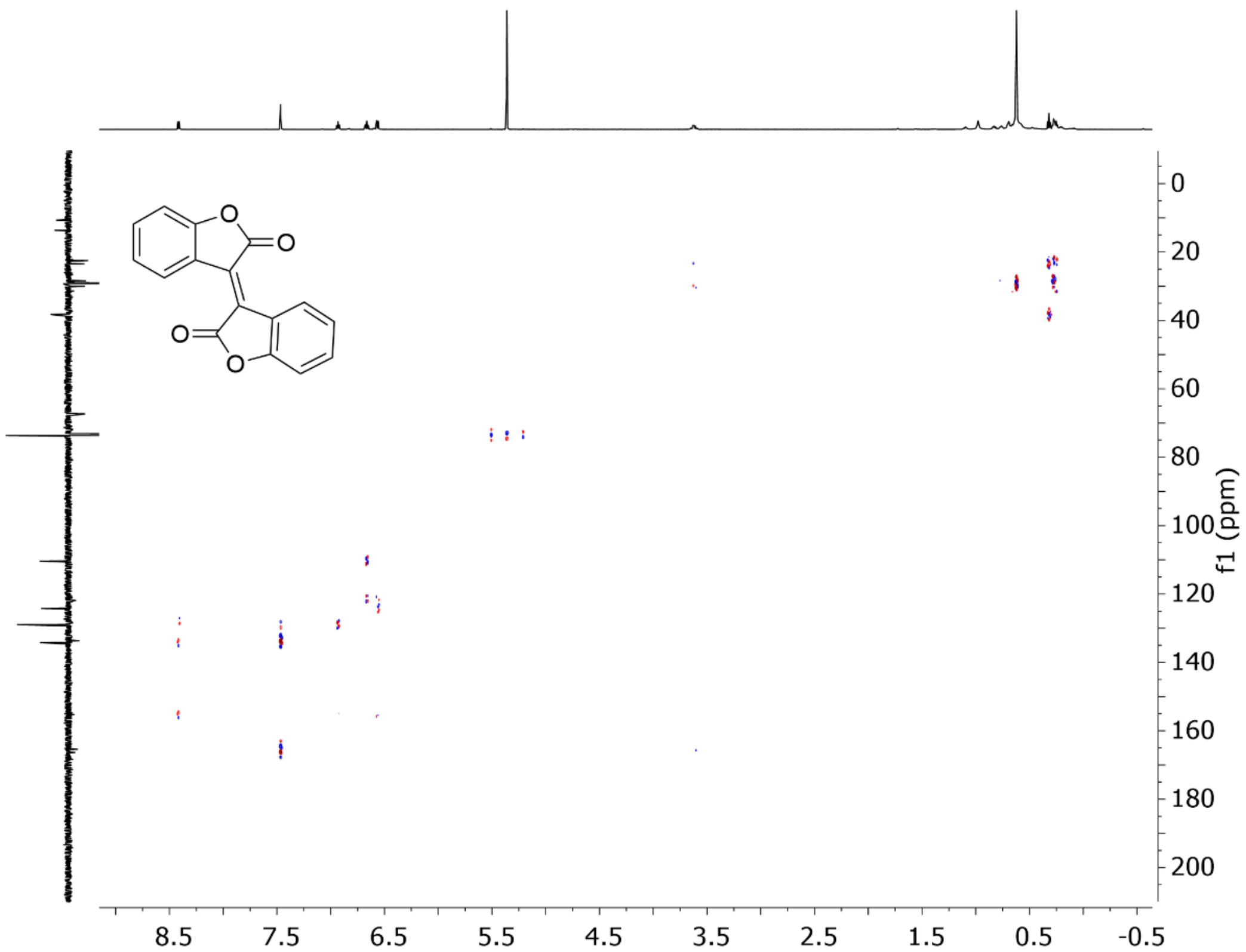

Figure S39. ^{1}H-^{13}C HMBC spectrum of (**13**) in $CD_2D_2Cl_2$ at 298K.

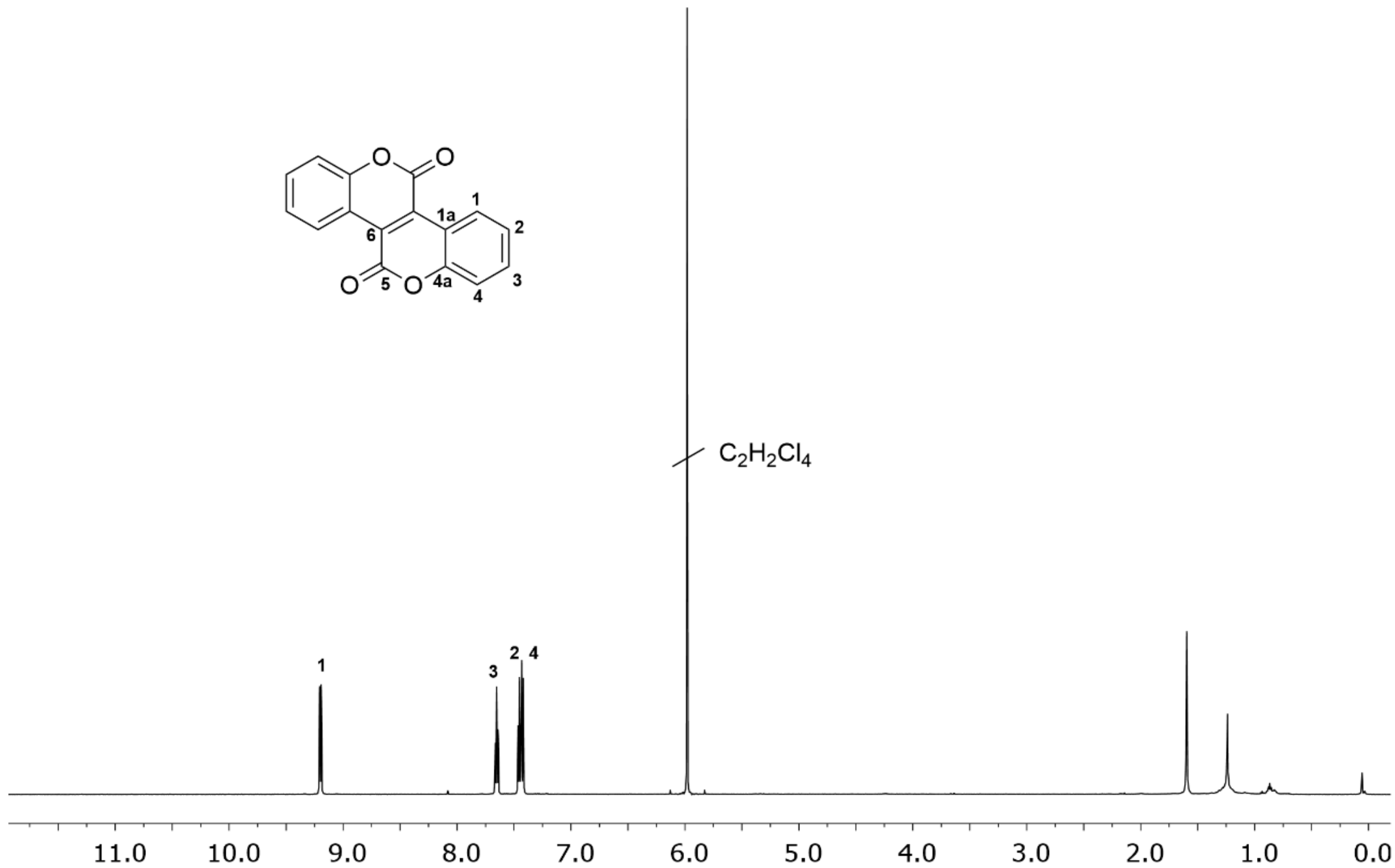

Figure S40. ^{1}H-NMR spectrum of (**14**) in $CD_2D_2Cl_2$ at 298K.

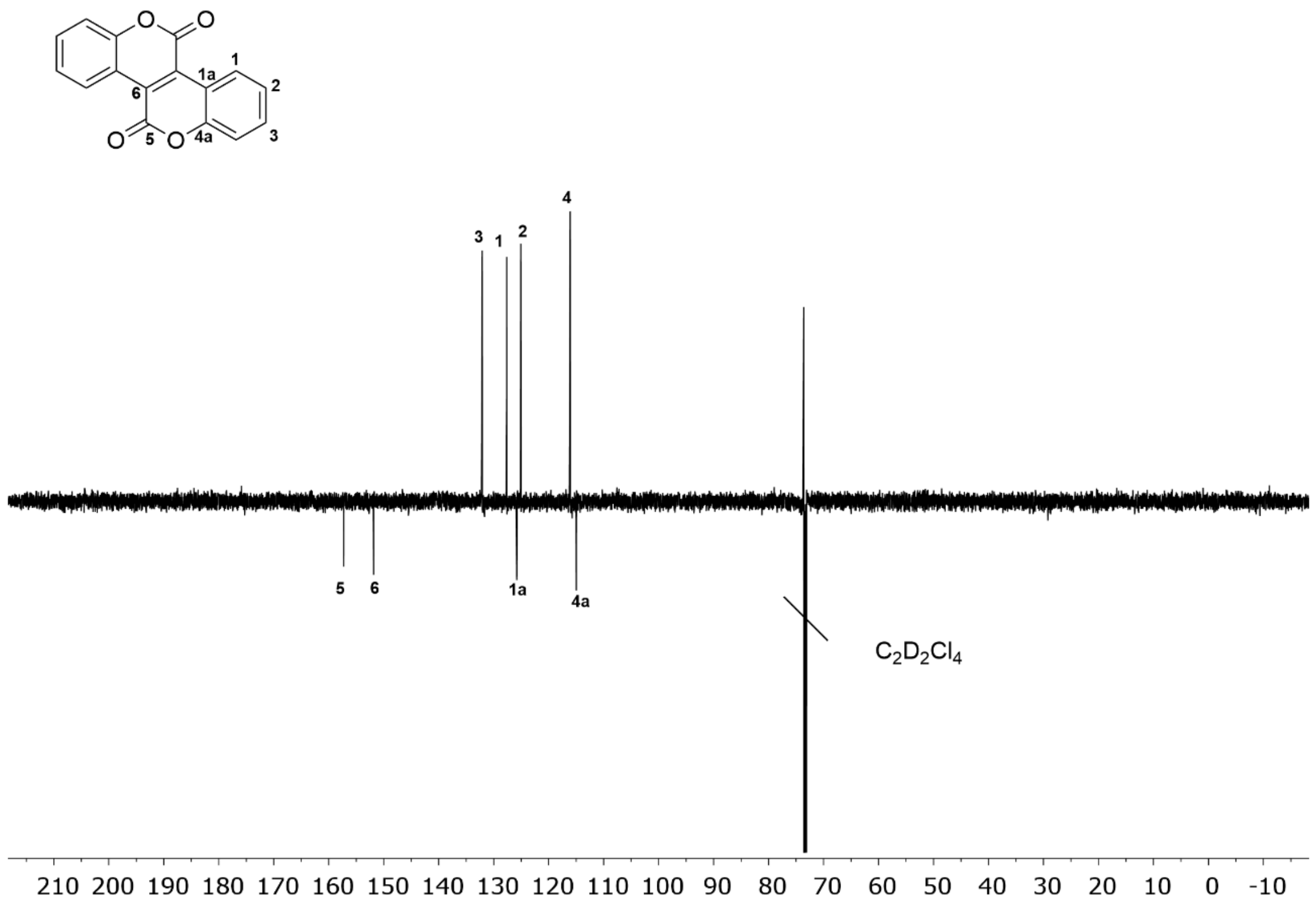

Figure S41. $^{13}C\{^{1}H\}$-APT spectrum of (**14**) in $CD_2D_2Cl_2$ at 298K.

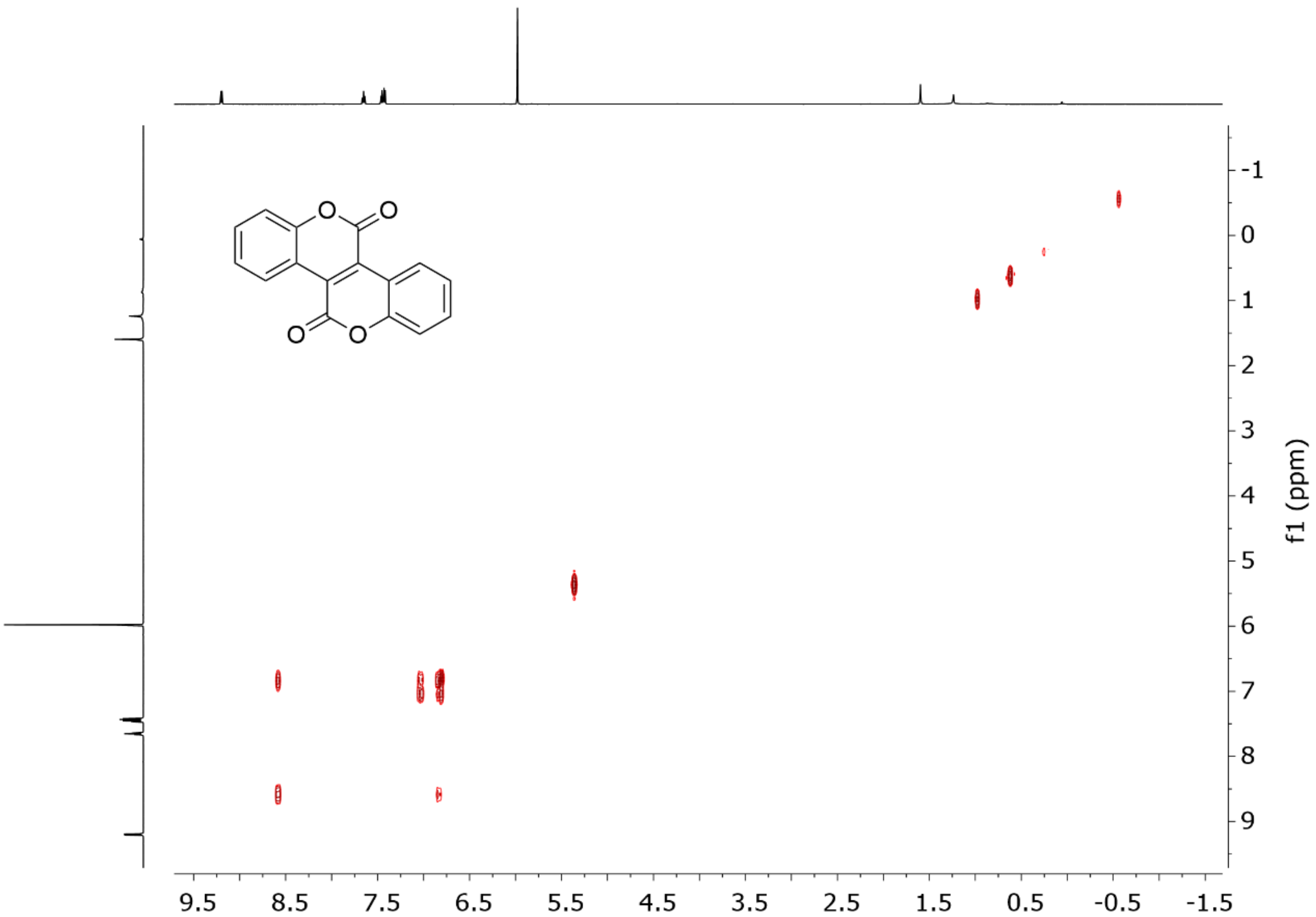

Figure S42. ^{1}H-^{1}H COSY spectrum of (**14**) in $CD_2D_2Cl_2$ at 298K.

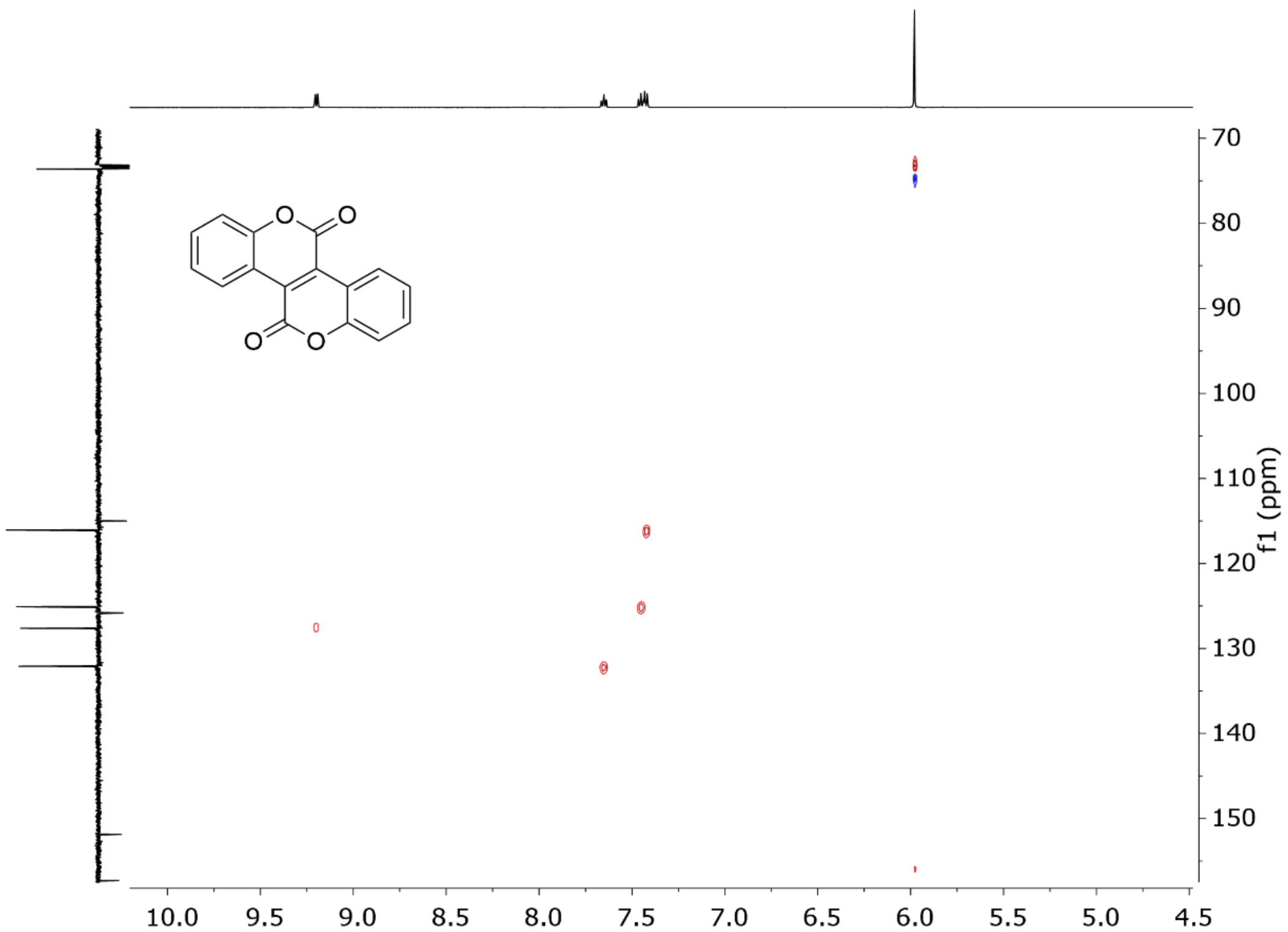

Figure S43. ^{1}H-^{13}C HSQC spectrum of (**14**) in $CD_2D_2Cl_2$ at 298K.

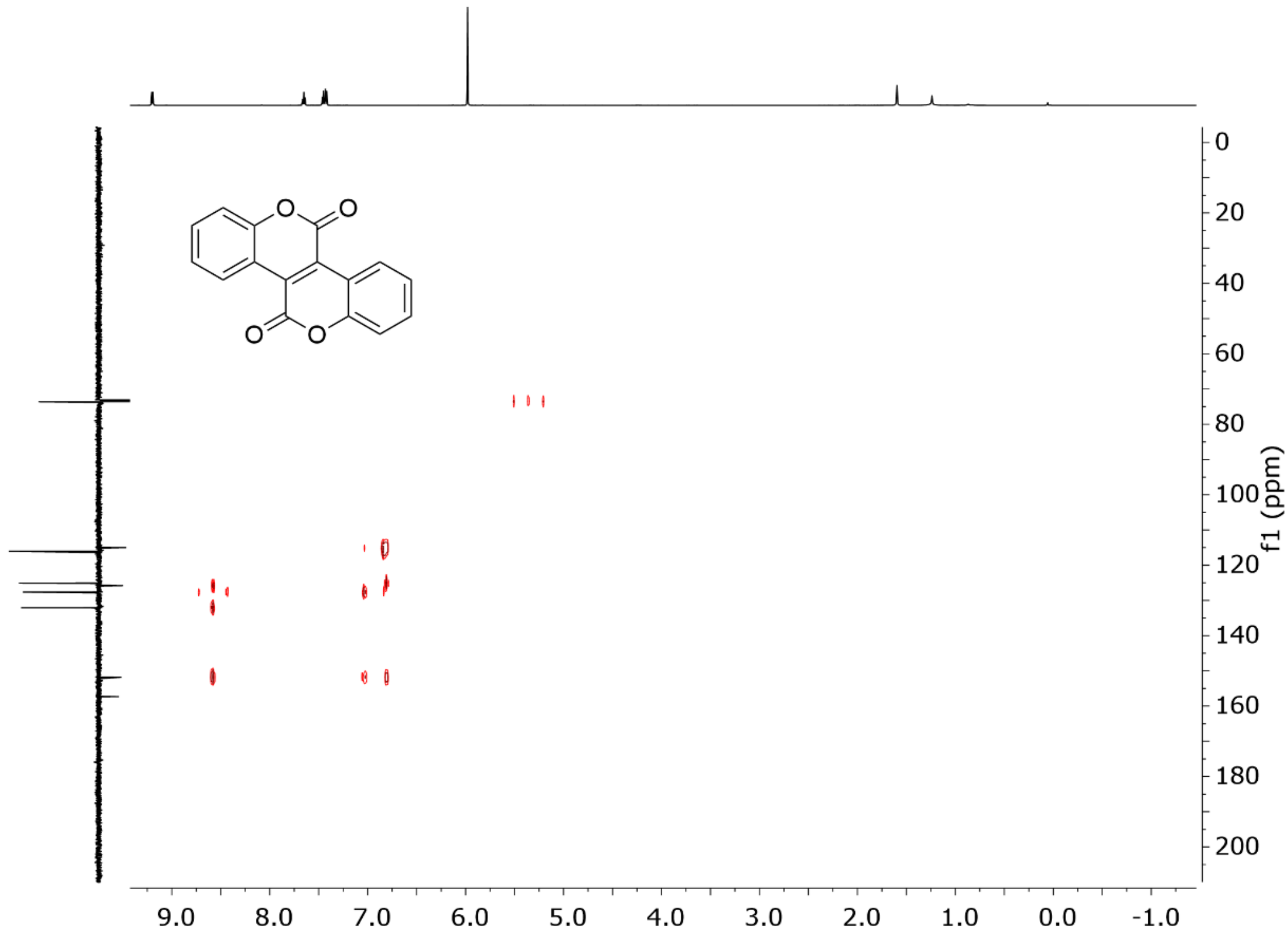

Figure S44. ^{1}H-^{13}C HMBC spectrum of (**14**) in $CD_2D_2Cl_2$ at 298K.

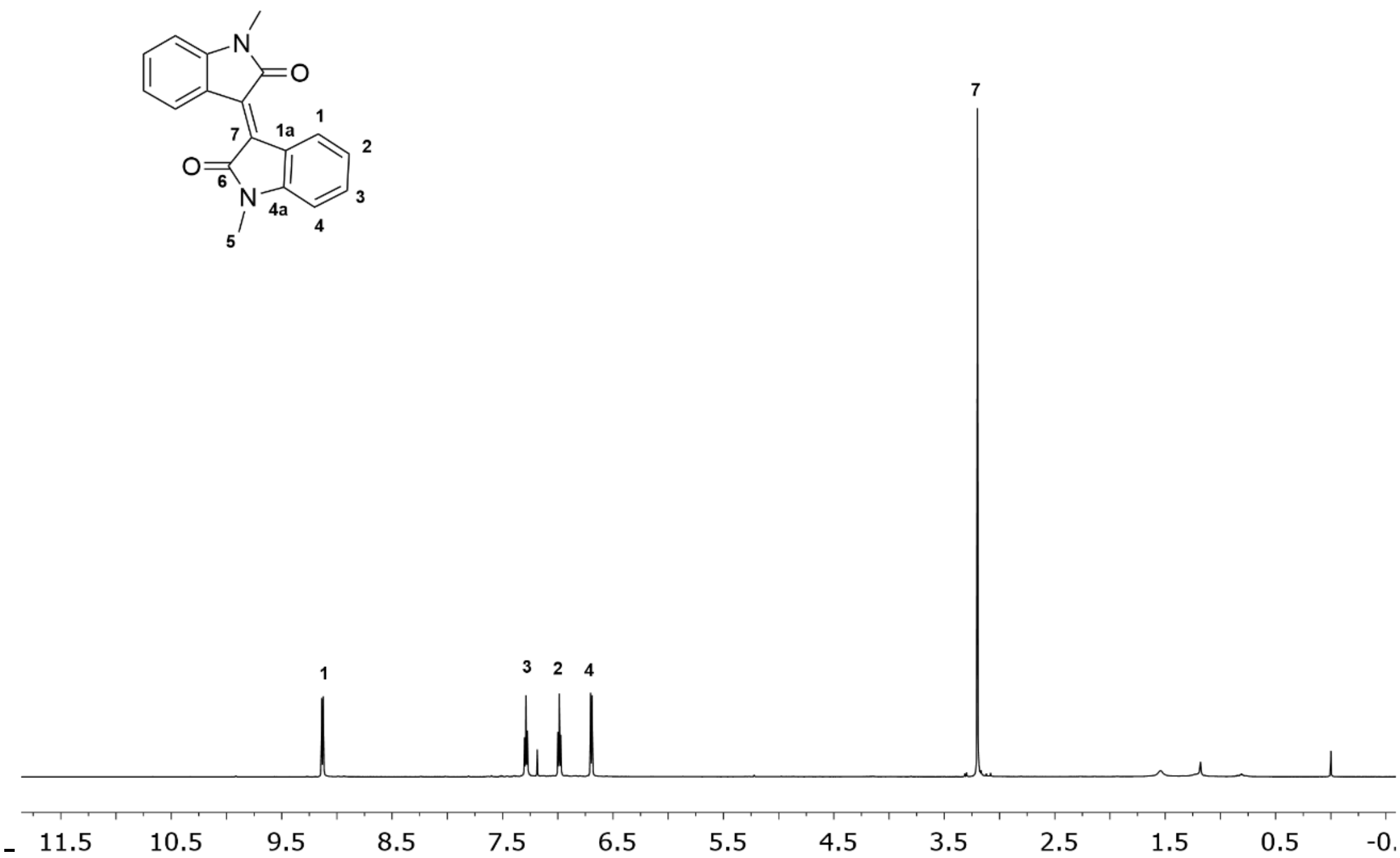

Figure S45. ^{1}H-NMR spectrum of (**15**) in $CDCl_3$ at 298K.

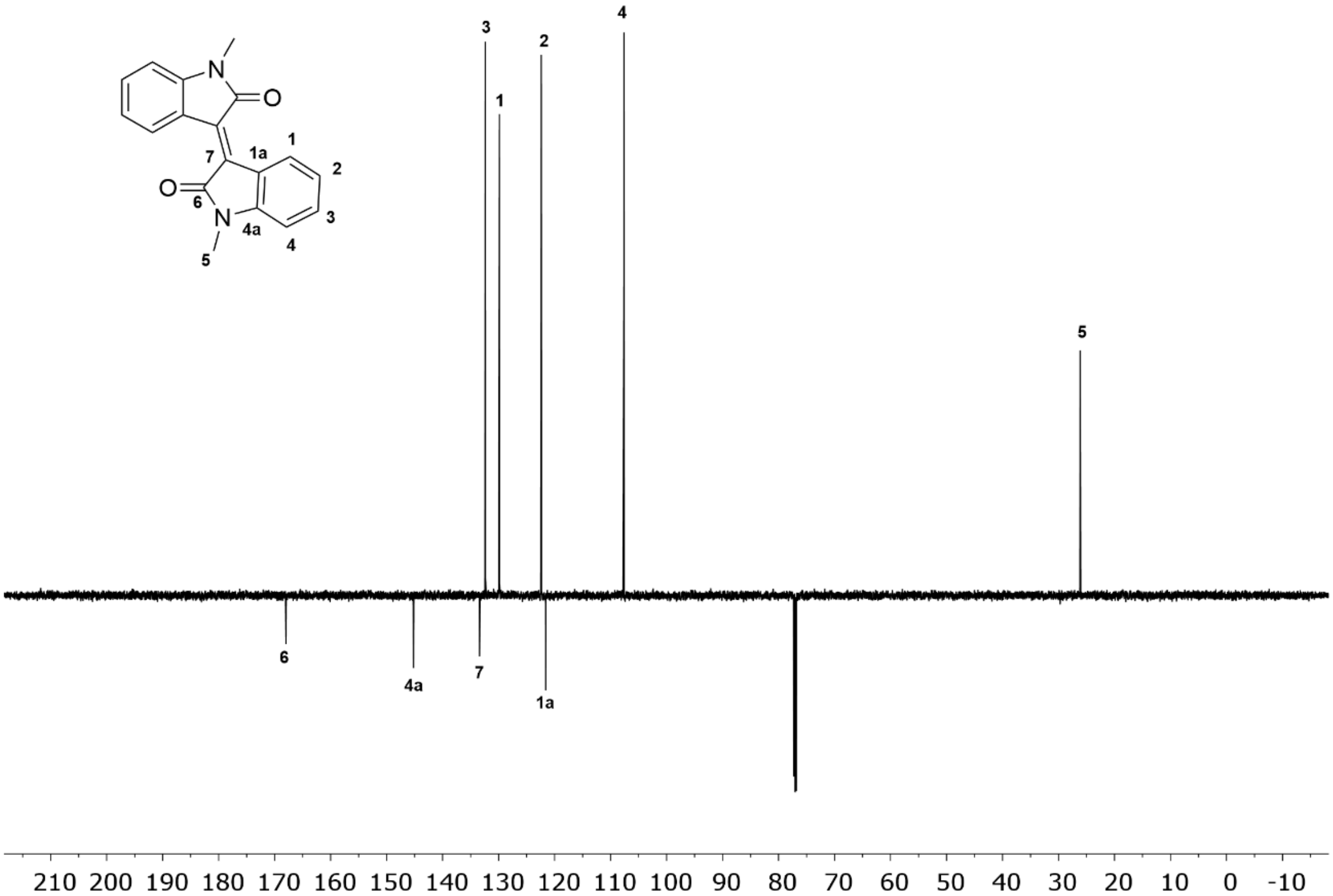

Figure S46. $^{13}C\{^{1}H\}$-APT spectrum of (**15**) in $CDCl_3$ at 298K

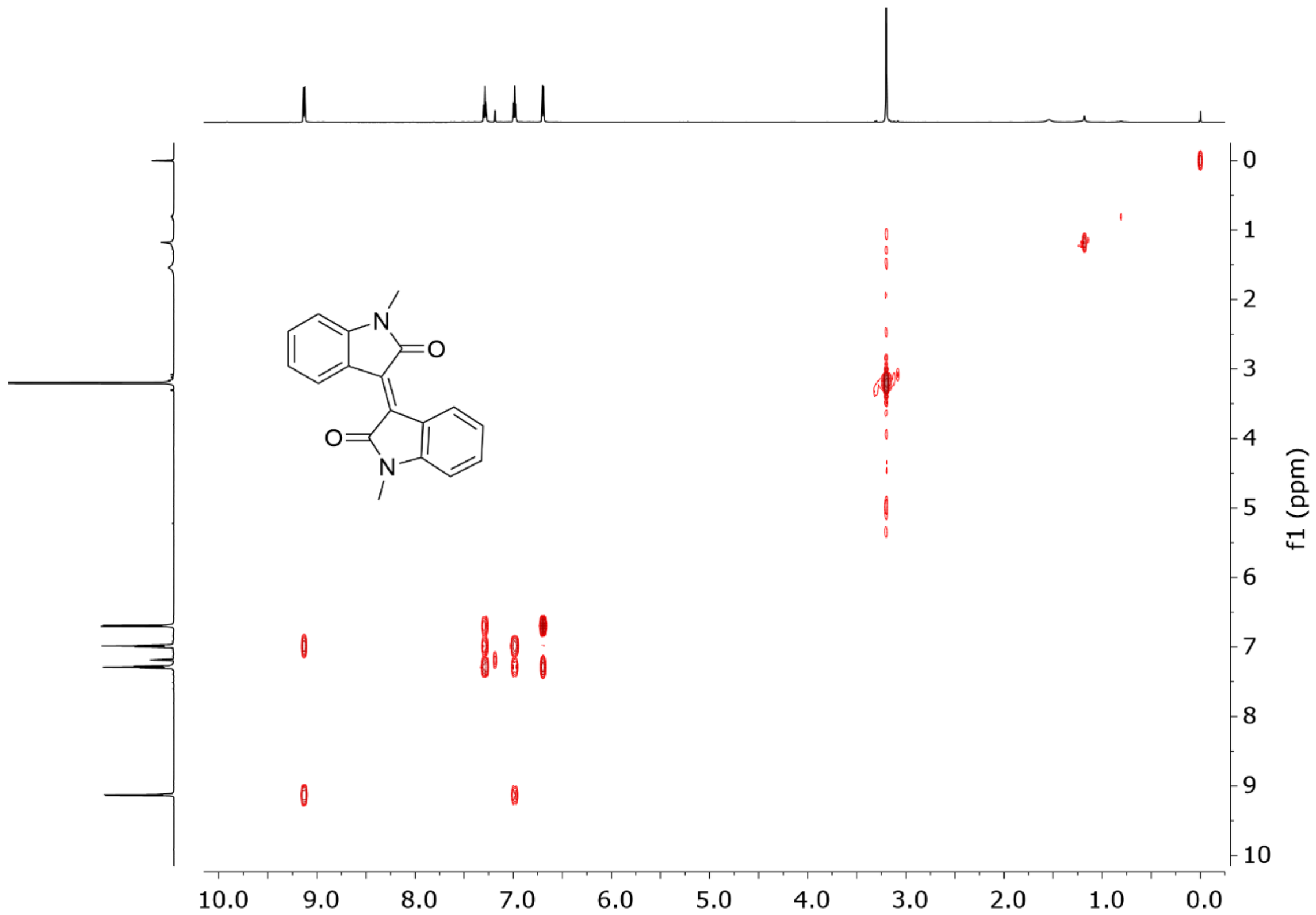

Figure S47. ^{1}H-^{1}H COSY spectrum of (**15**) in $CDCl_3$ at 298K.

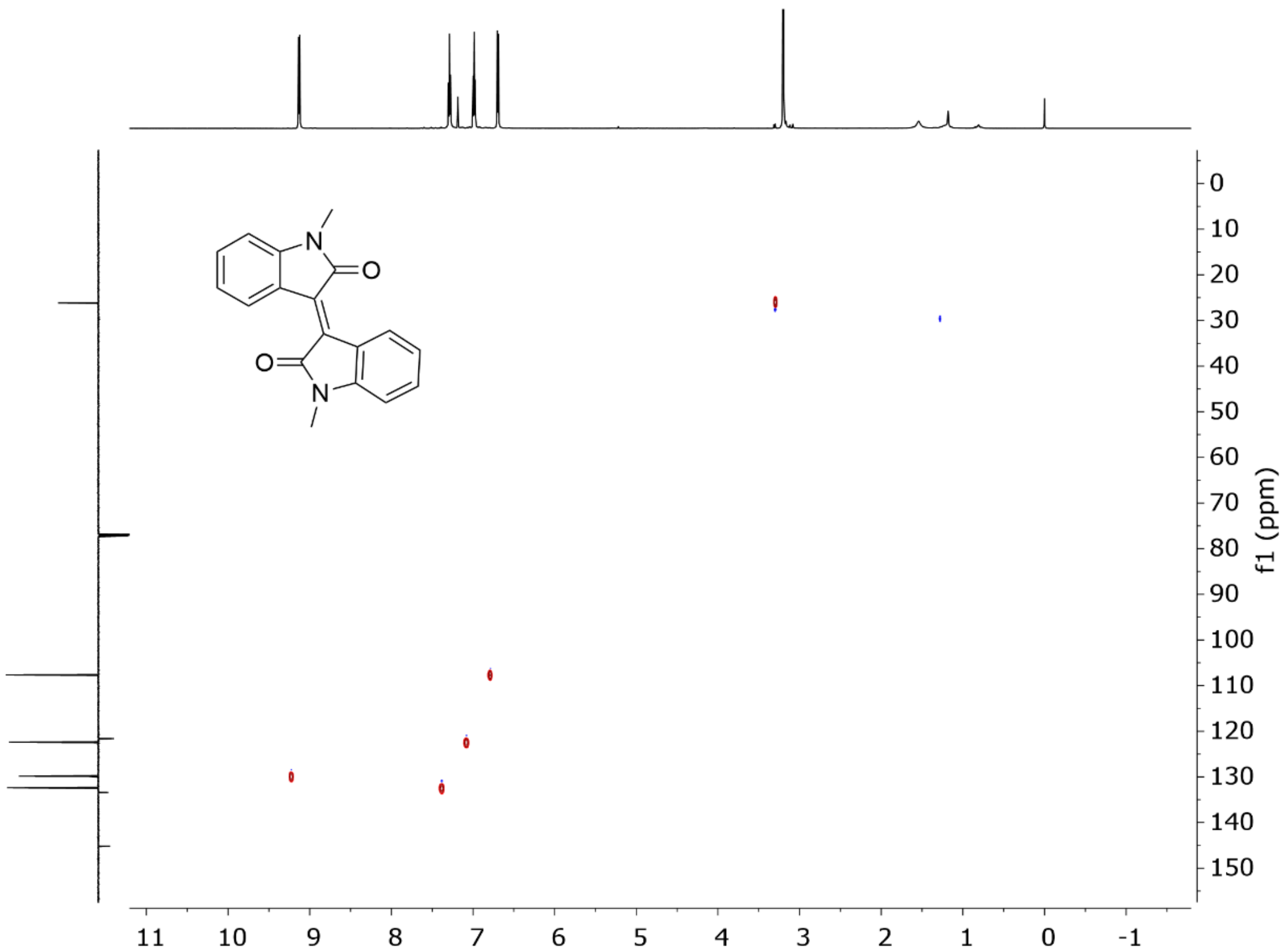

Figure S48. ^{1}H-^{13}C HSQC spectrum of (**15**) in CDCl3 at 298K.

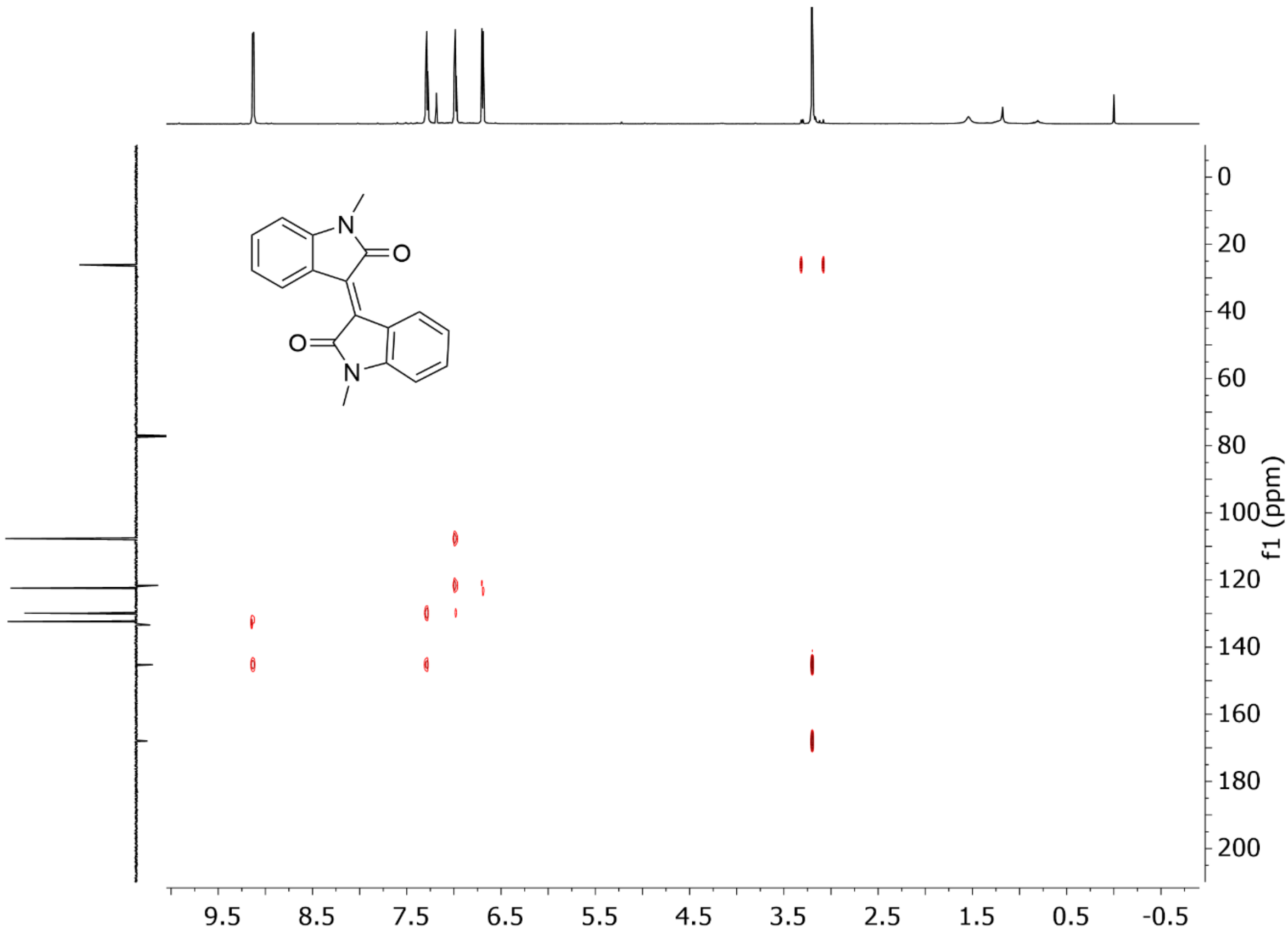

Figure S49. ^{1}H-^{13}C HMBC spectrum of (**15**) in $CDCl_3$ at 298K.

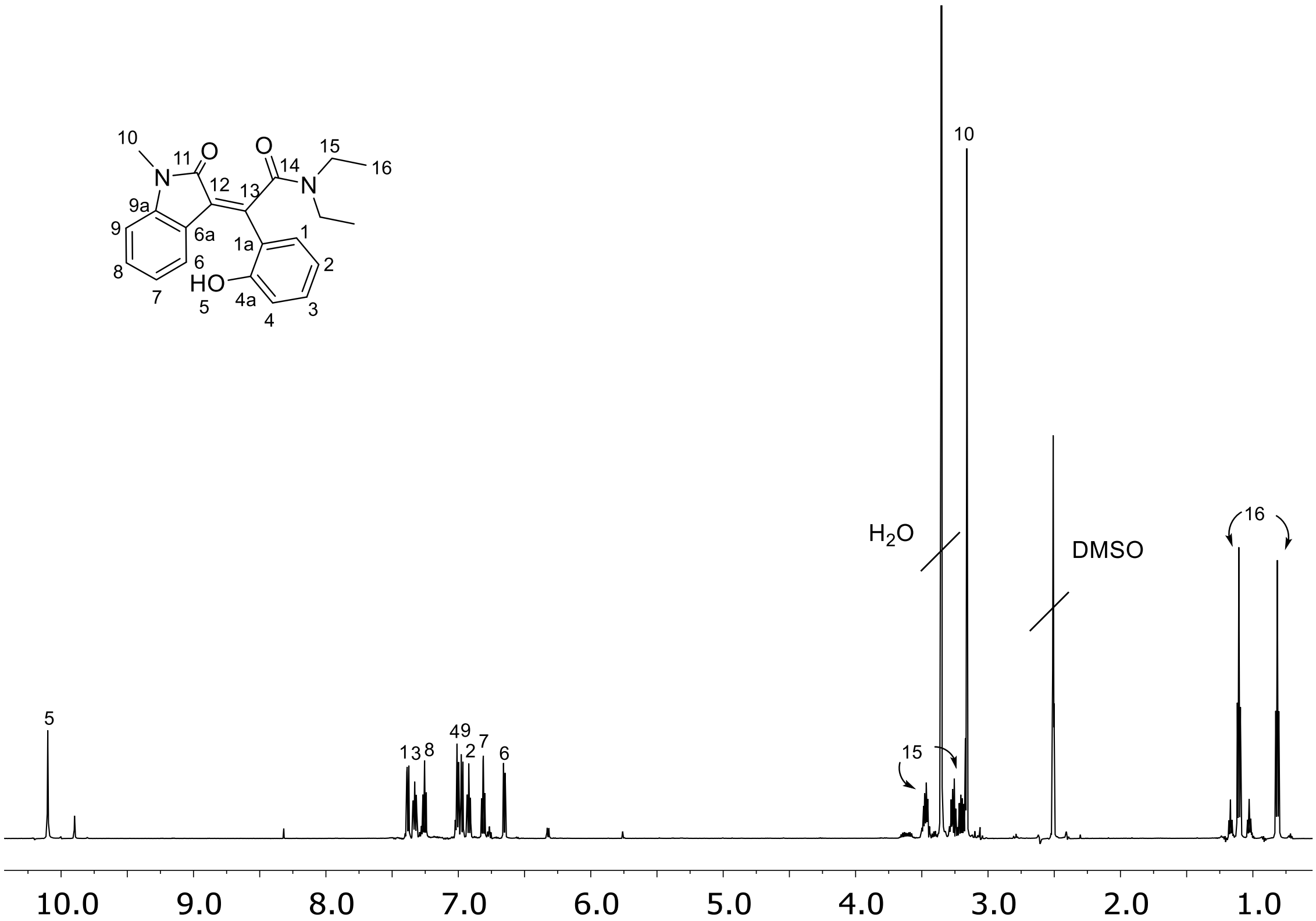

Figure S50. ^{1}H-NMR spectrum of (**16**) in DMSO-d6 at 298K.

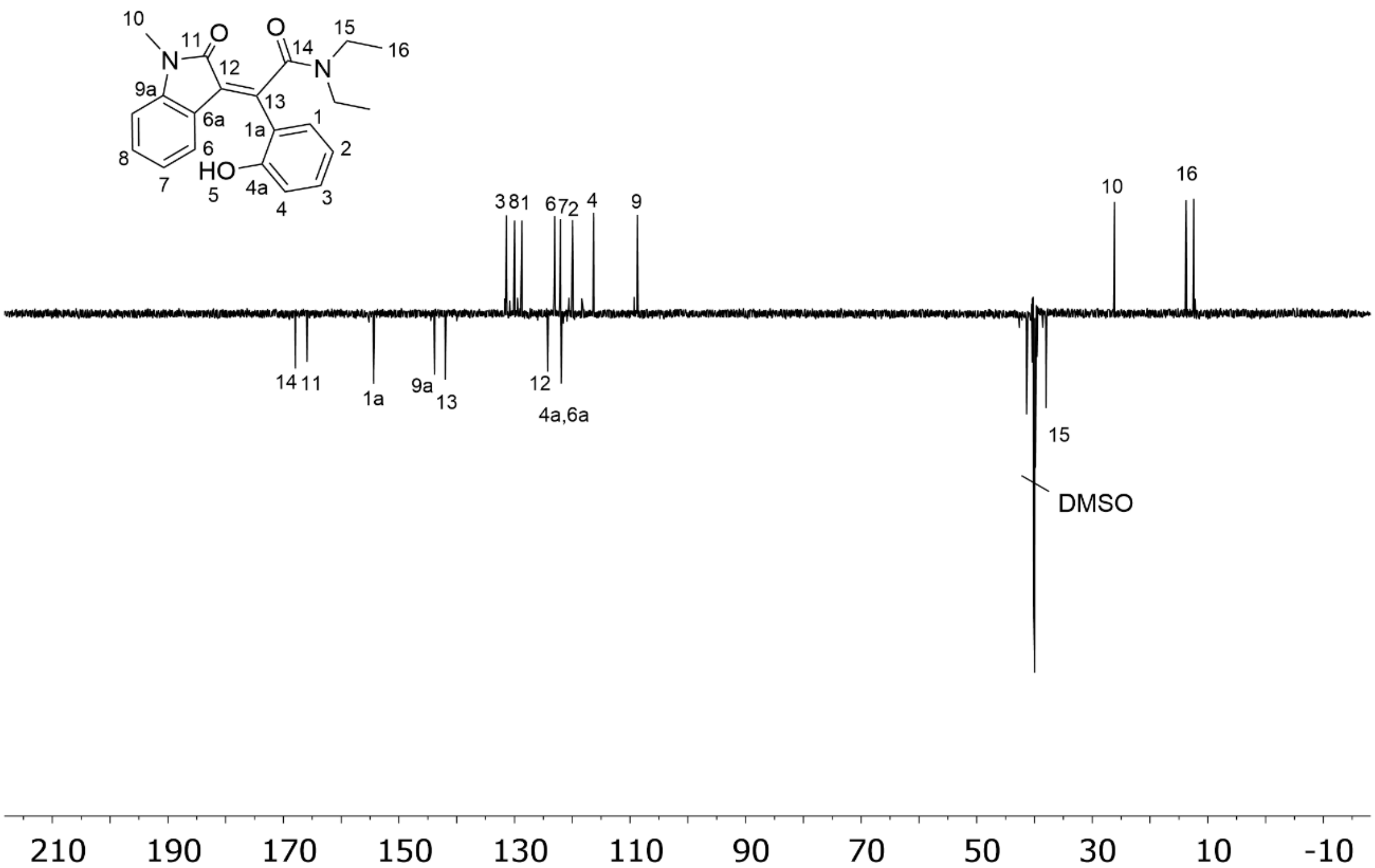

Figure S51. ^{13}C-NMR spectrum of (**16**) in DMSO-d6 at 298K.

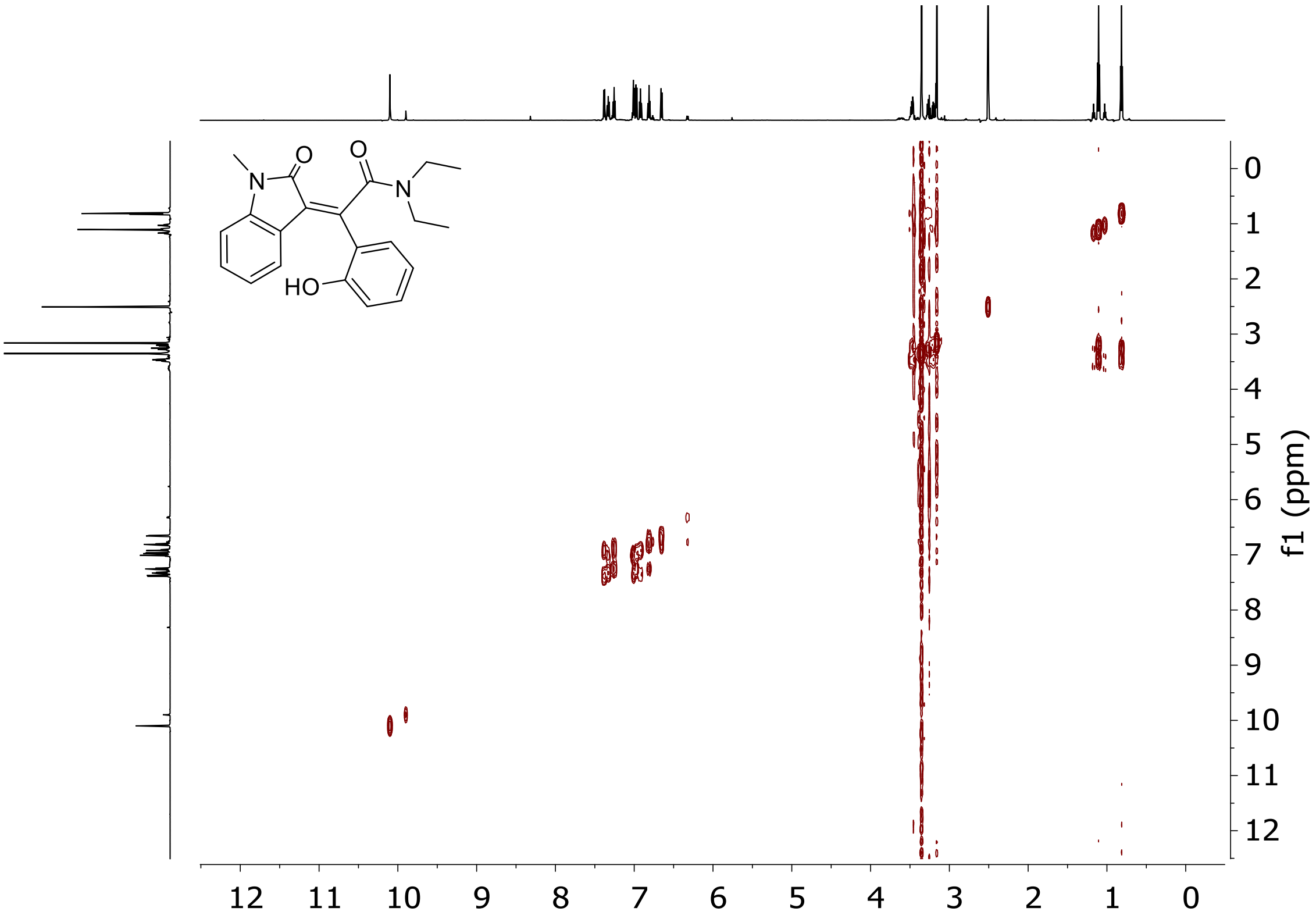

Figure S52. ^{1}H-^{1}H COSY spectrum of (**16**) in DMSO-d6 at 298K.

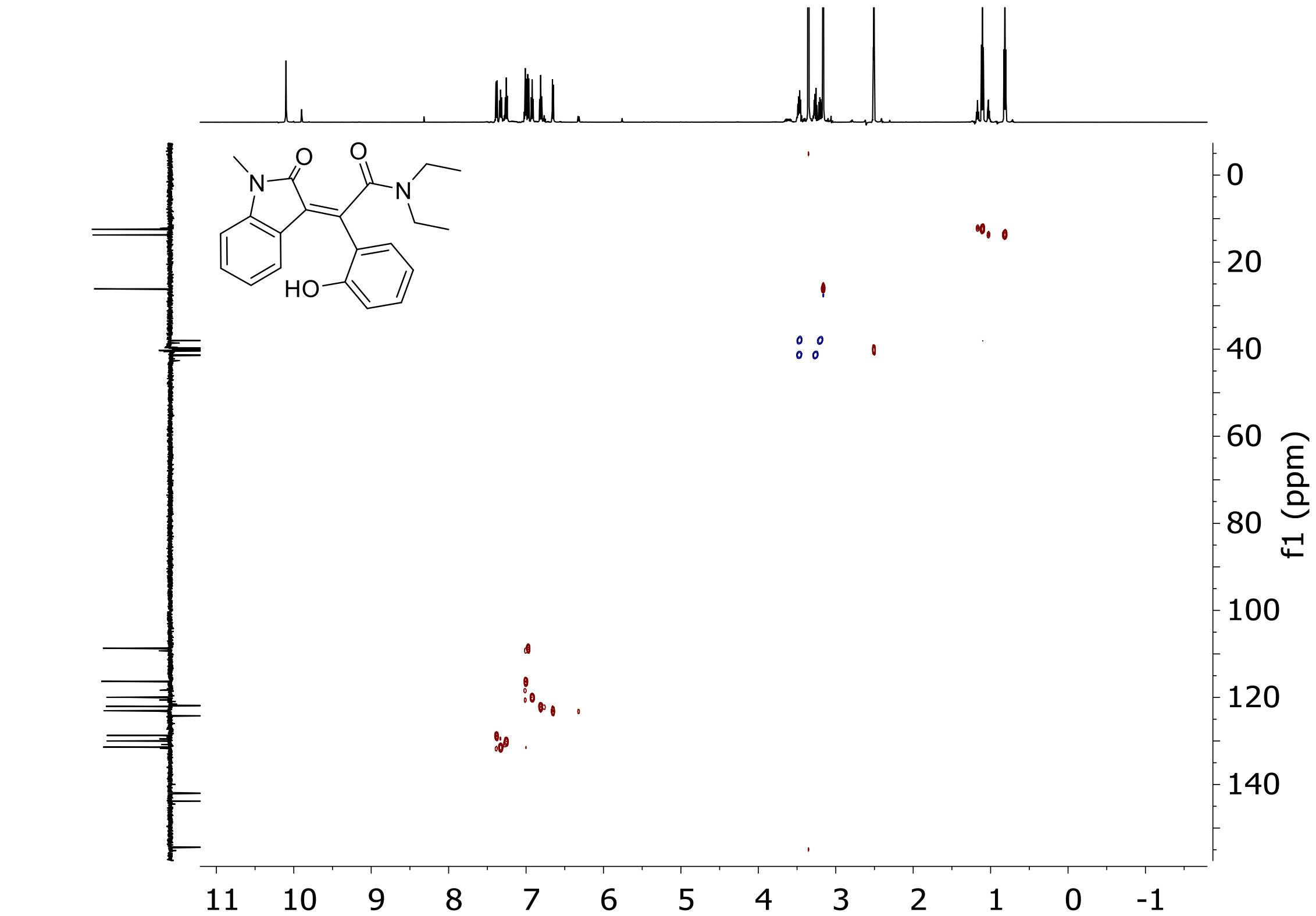

Figure S53. ^{1}H-^{13}C HSQC spectrum of (**16**) in DMSO-d6 at 298K.

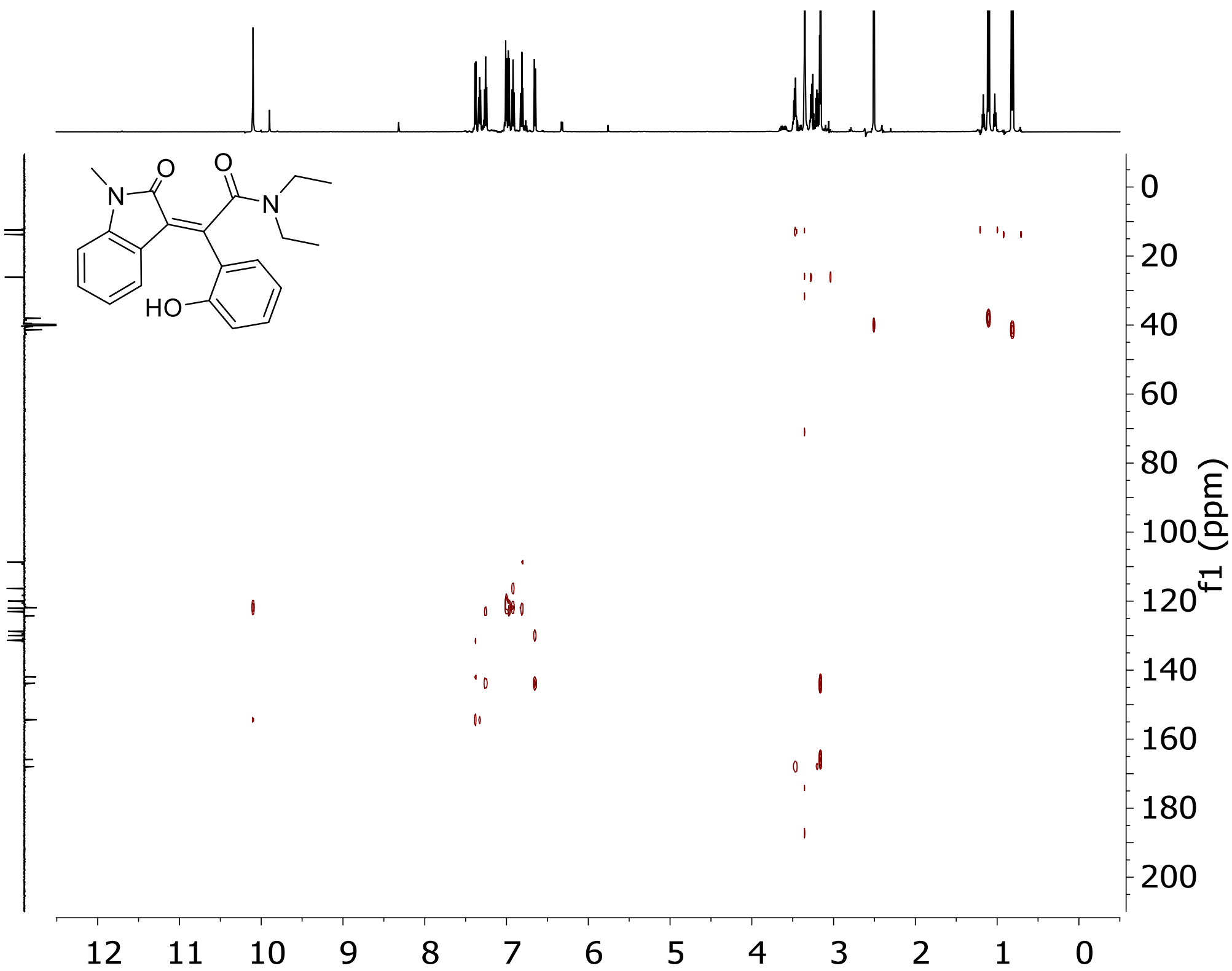

Figure S54. ^{1}H-^{13}C HMBC spectrum of (**16**) in DMSO-d6 at 298K.

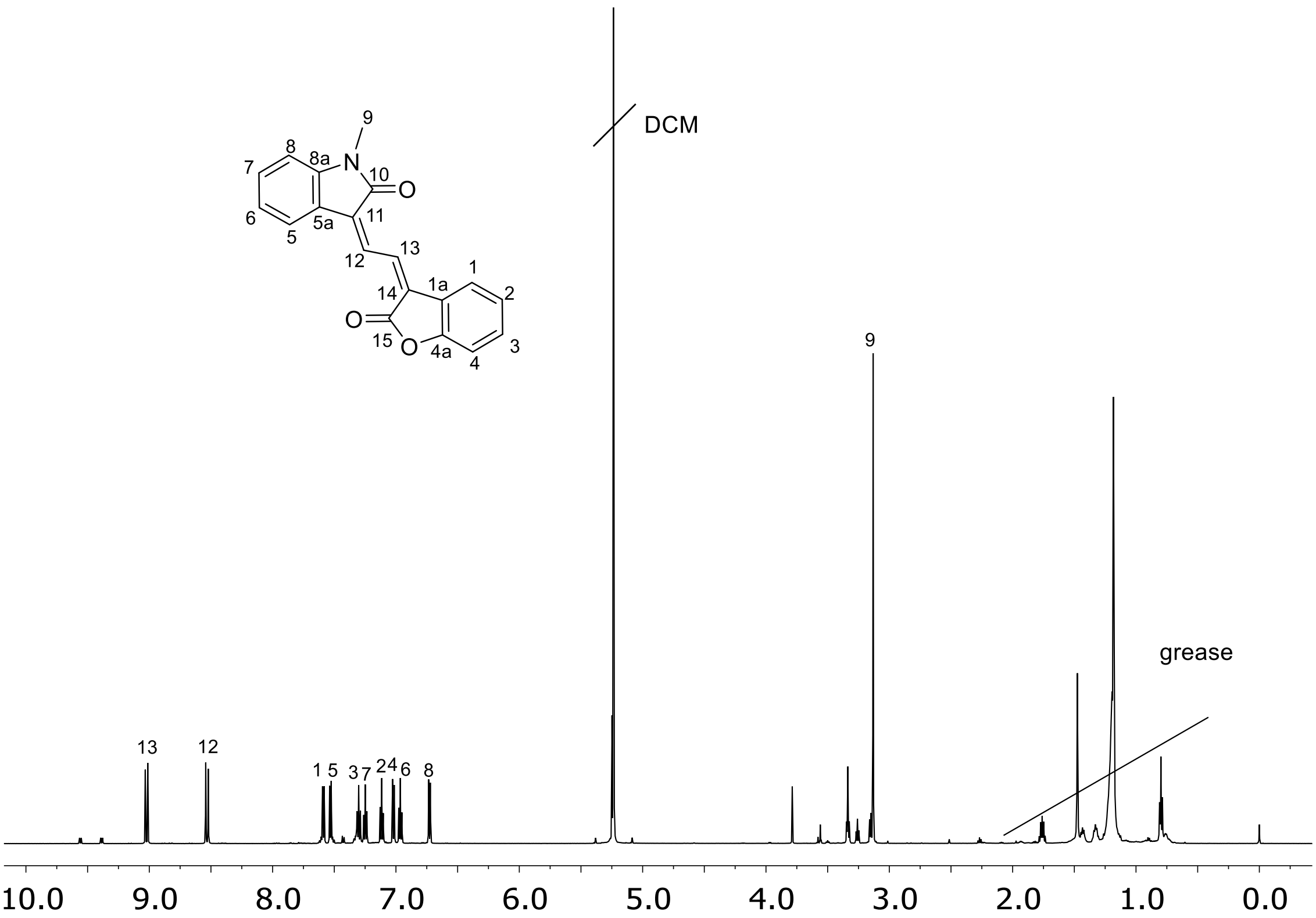

Figure S55. ^{1}H-NMR spectrum of (**17**) in DCM-d2 at 298K.

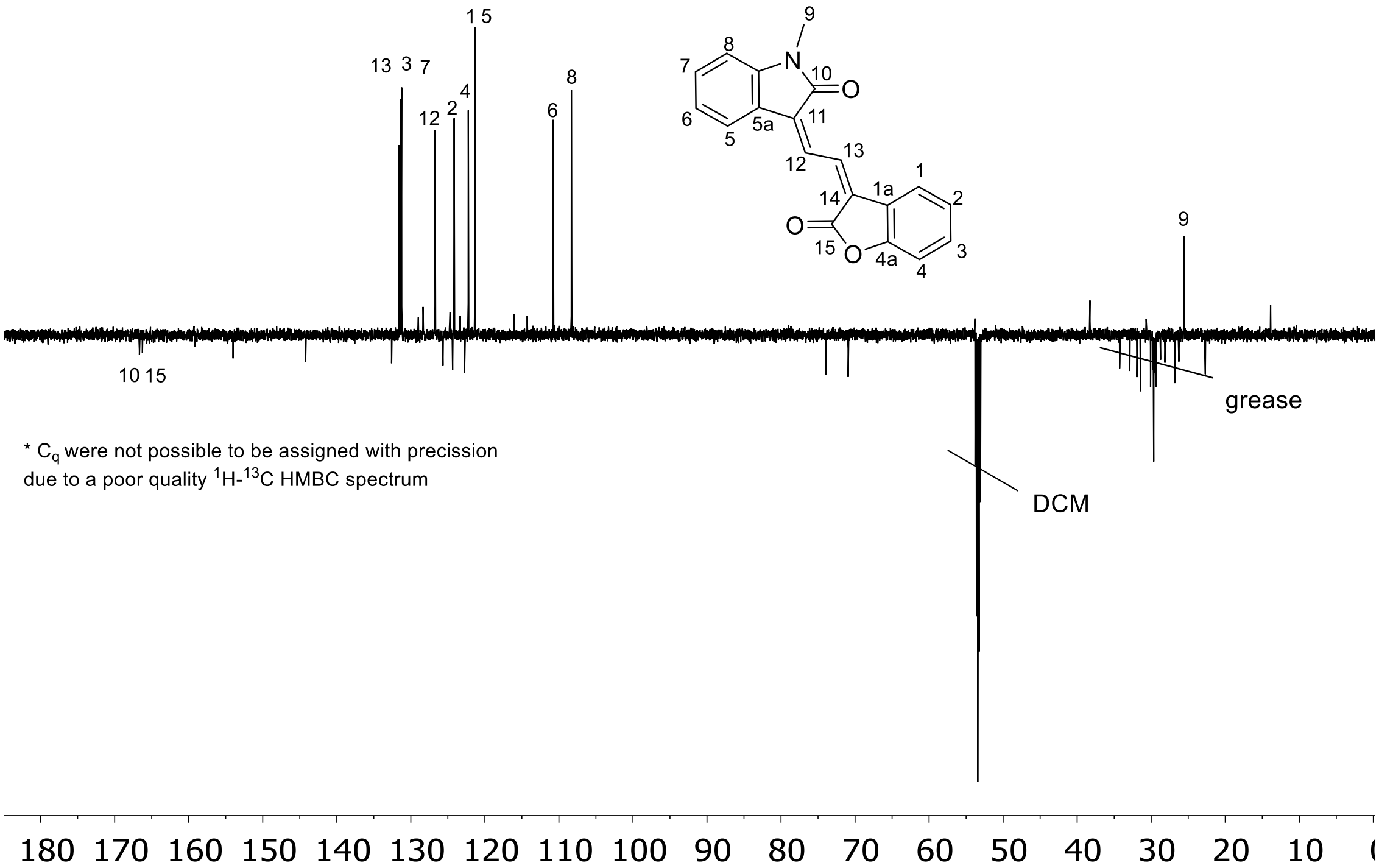

Figure S56. ^{13}C-NMR spectrum of (**17**) in DCM-d2 at 298K.

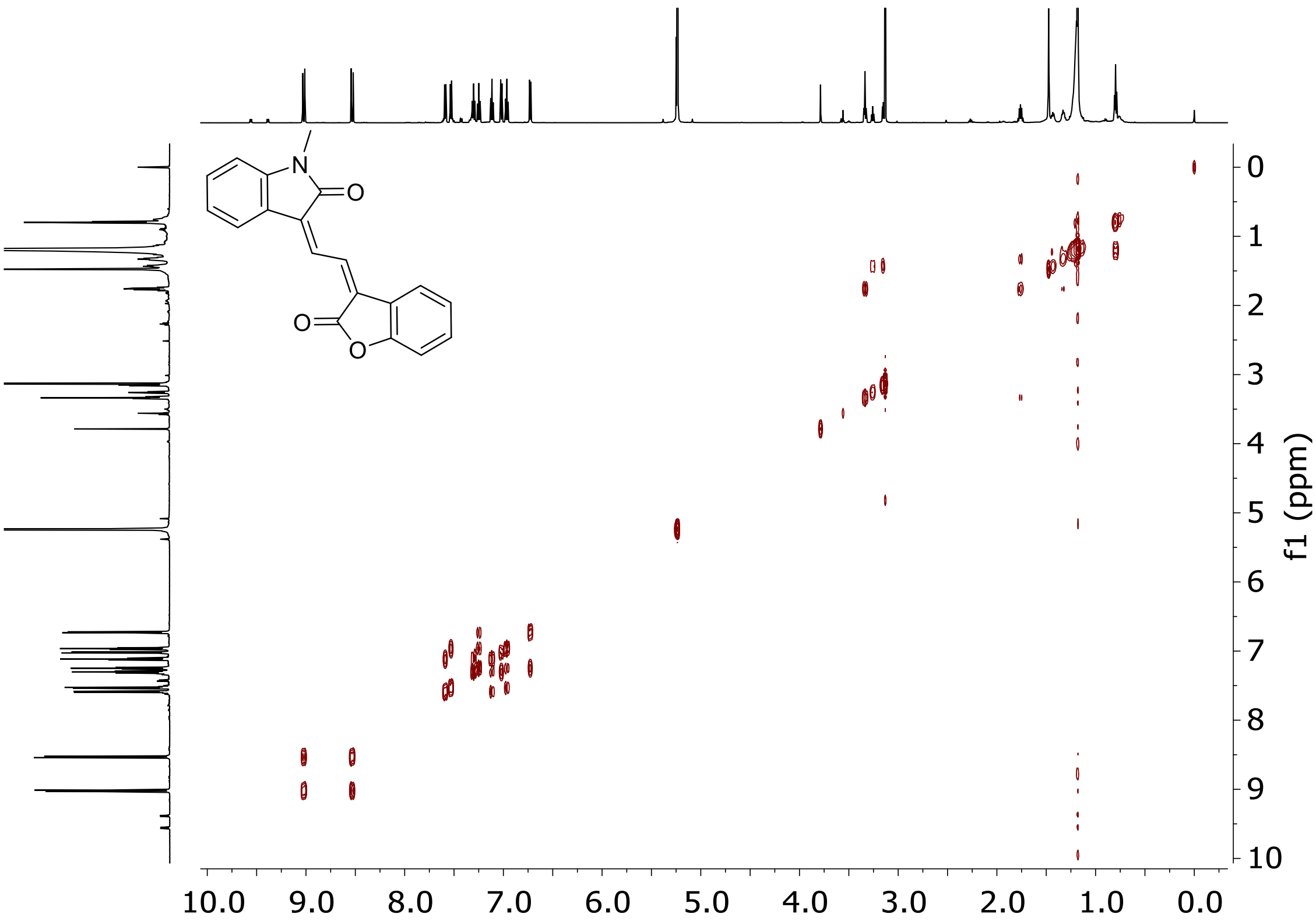

Figure S57. ^1H-^1H COSY spectrum of (**17**) in DCM-d2 at 298K.

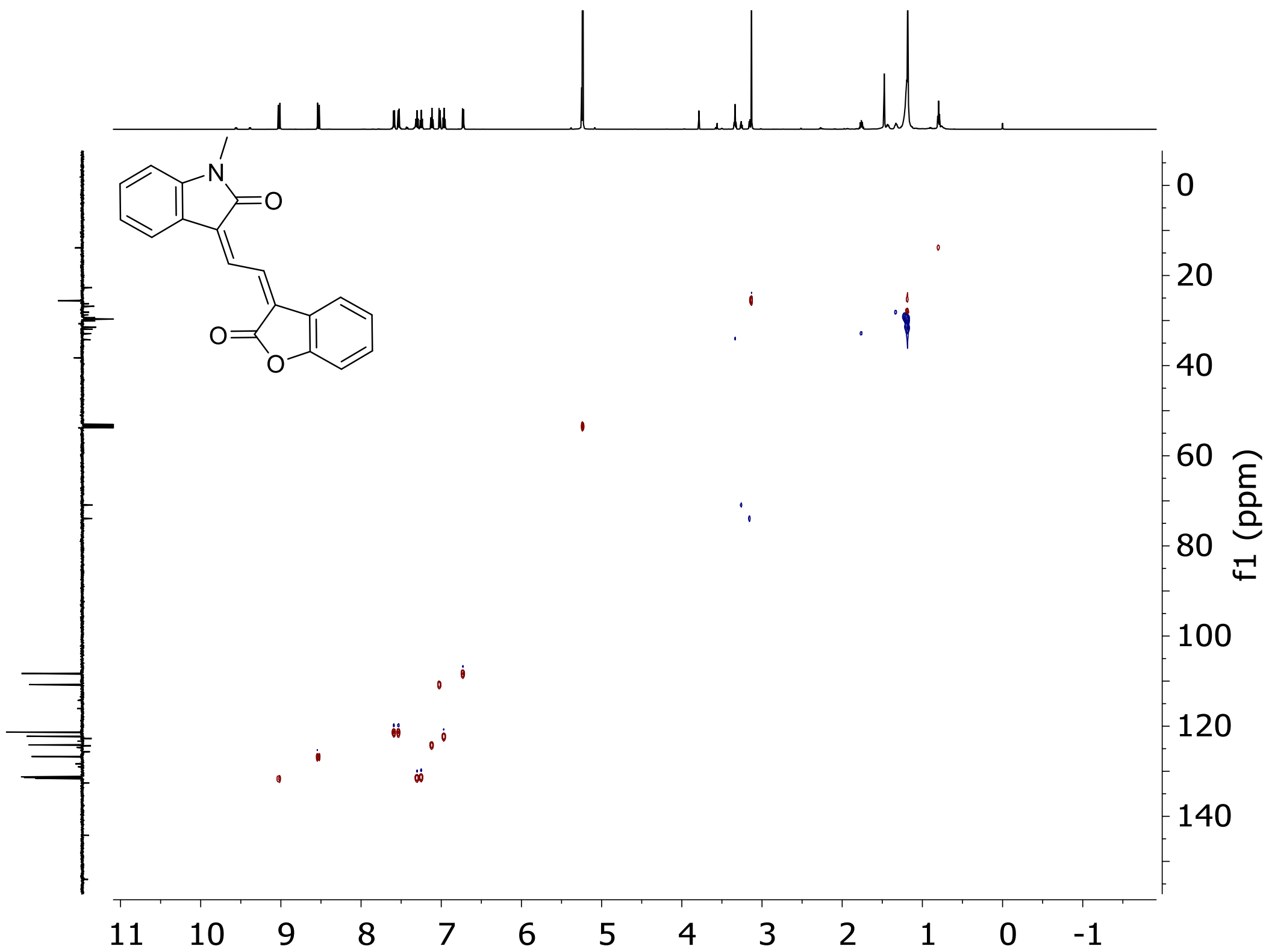

Figure S58. ^{1}H-^{13}C HSQC spectrum of (**17**) in DCM-d2 at 298K.

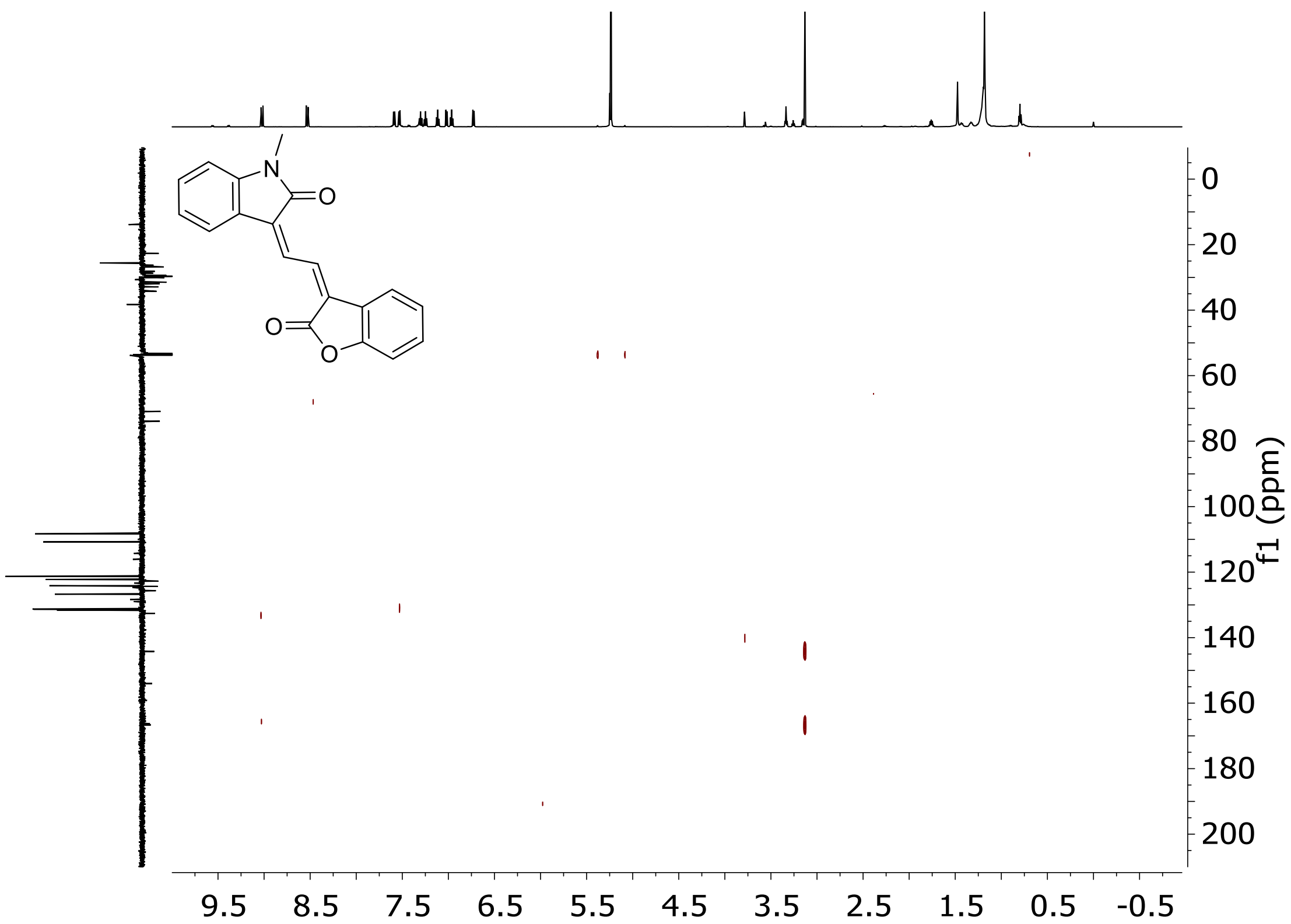

Figure S59. ^{1}H-^{13}C HMBC spectrum of (**17**) in DCM-d2 at 298K.

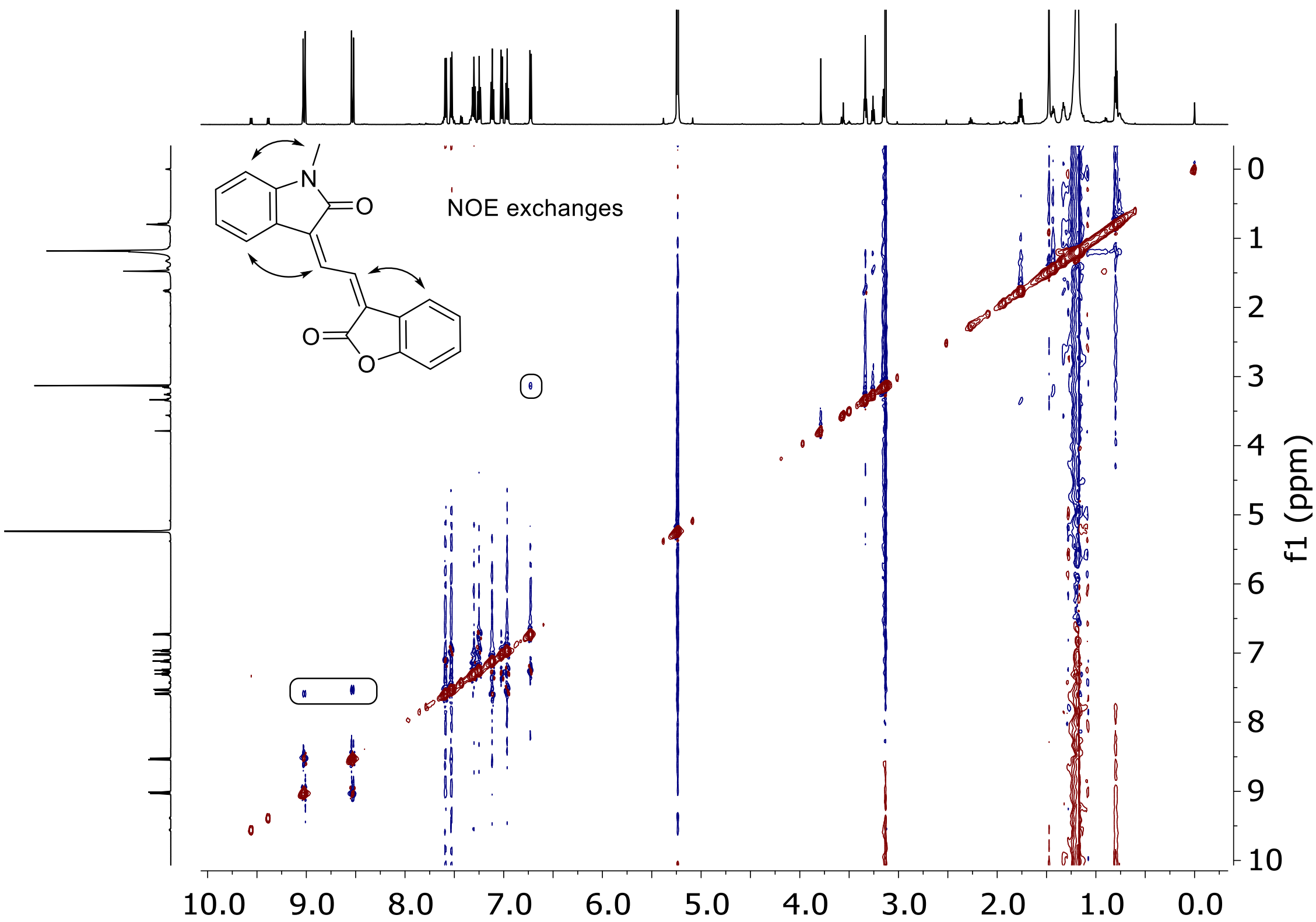

Figure S60. ^{1}H-^{1}H NOESY spectrum of (**17**) in DCM-d2 at 298K.

5. MALDI ToF MS data

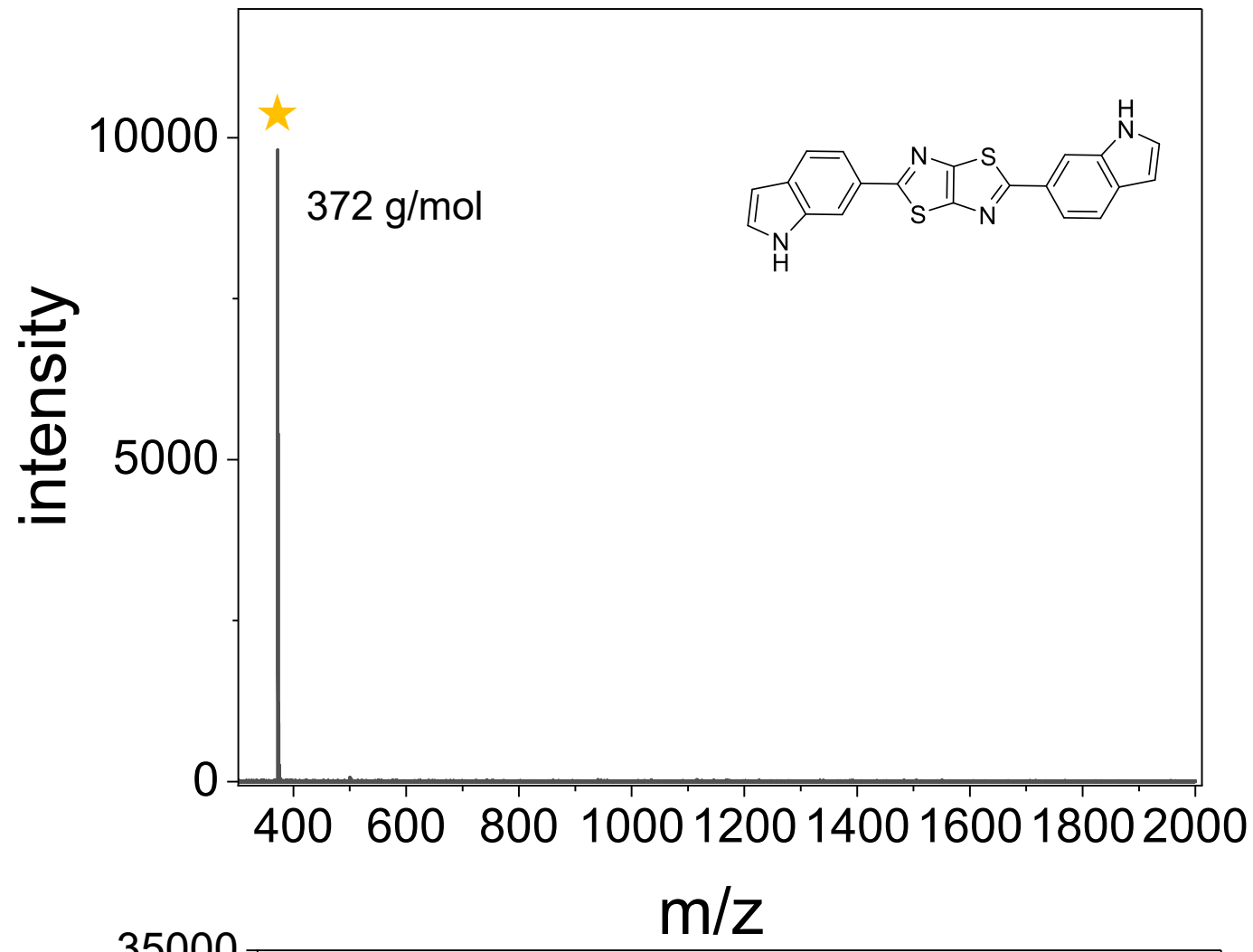

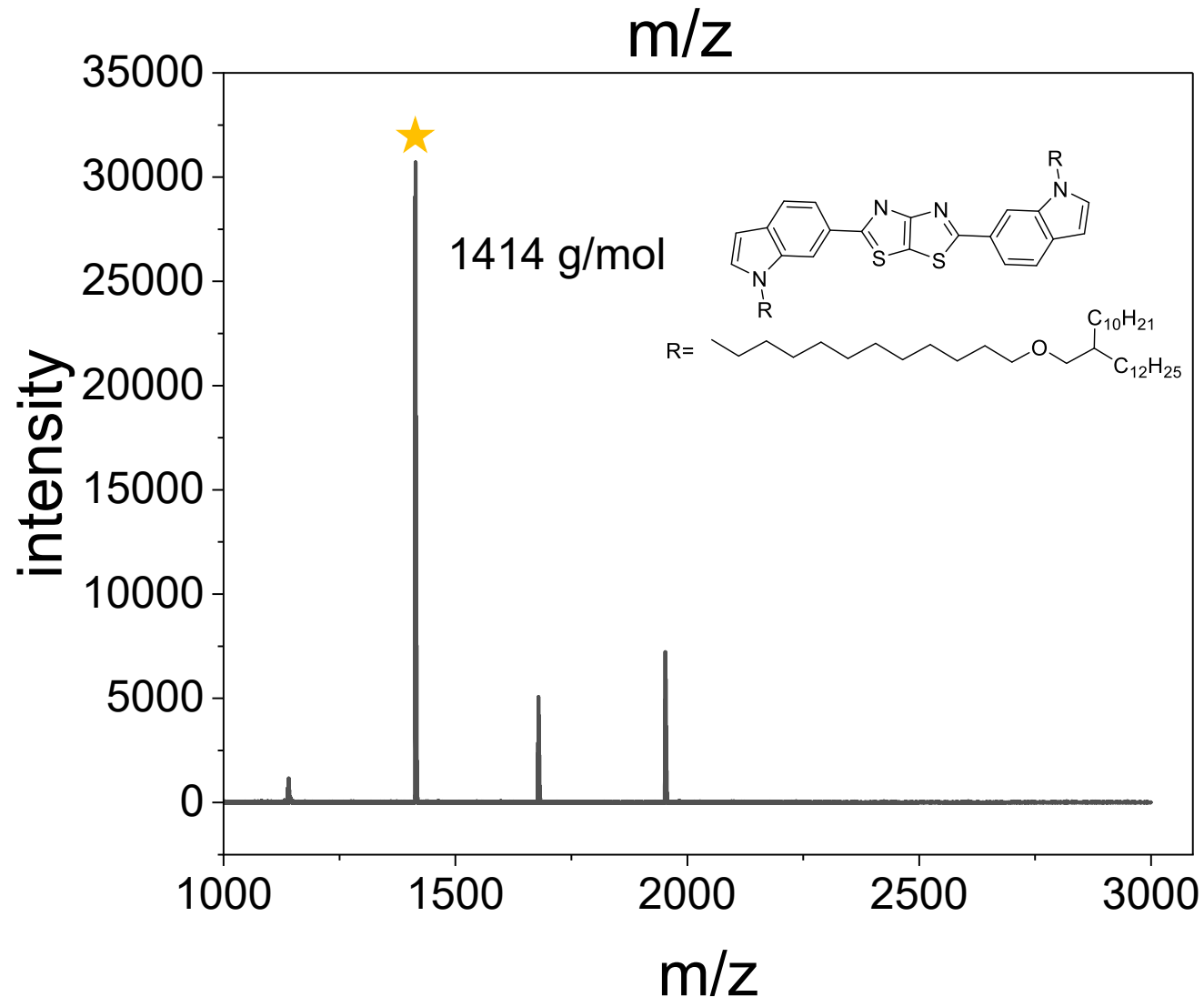

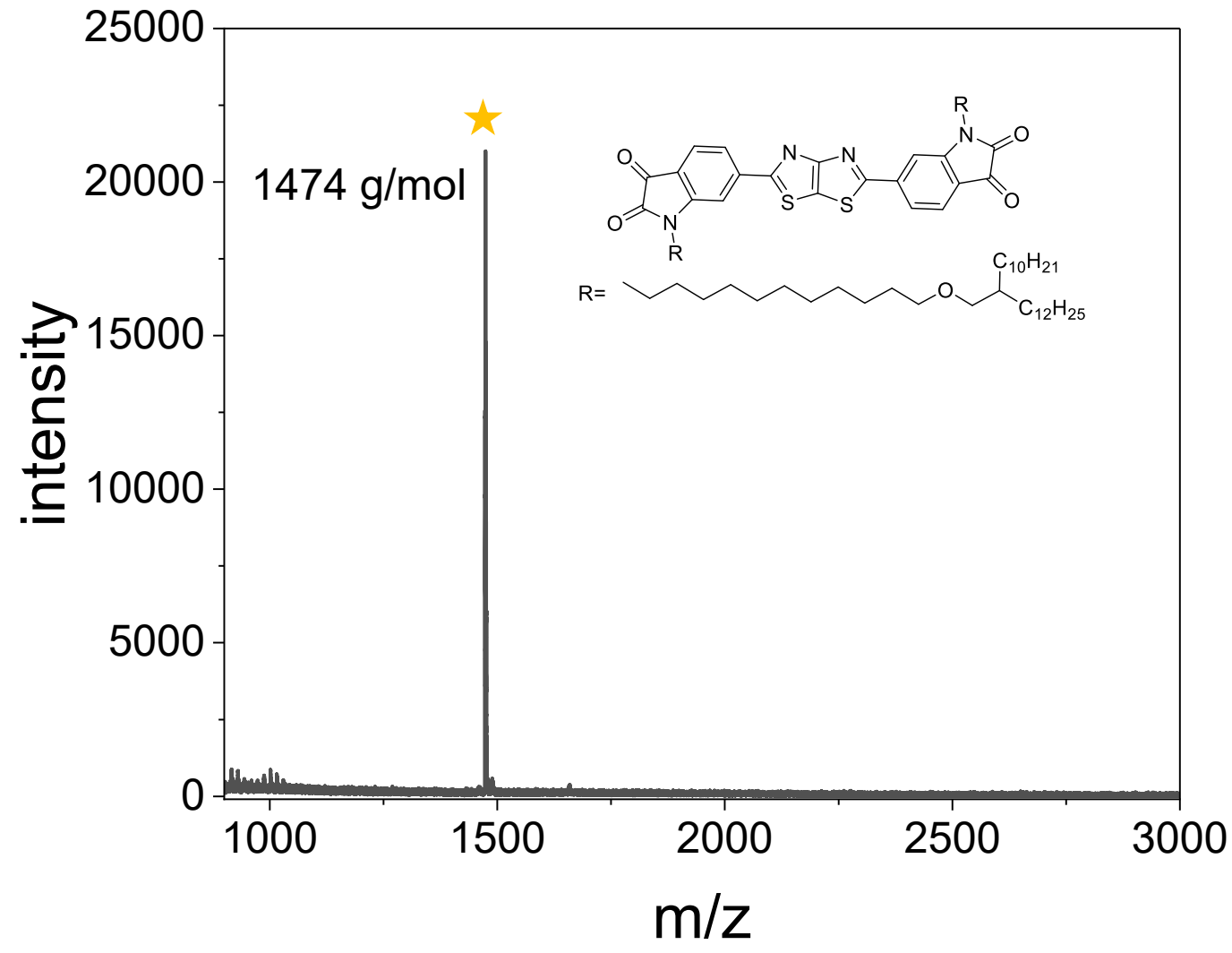

Figure S61. MALDI spectra of intermediates **2** (top), **3** (middle) and monomer 6,6 bisisatinthiazolo [5,4-d] thiazole **4** (bottom)**. 3** was not chromatographically purified. The additional signals are from unknown impurities and likely overestimated.

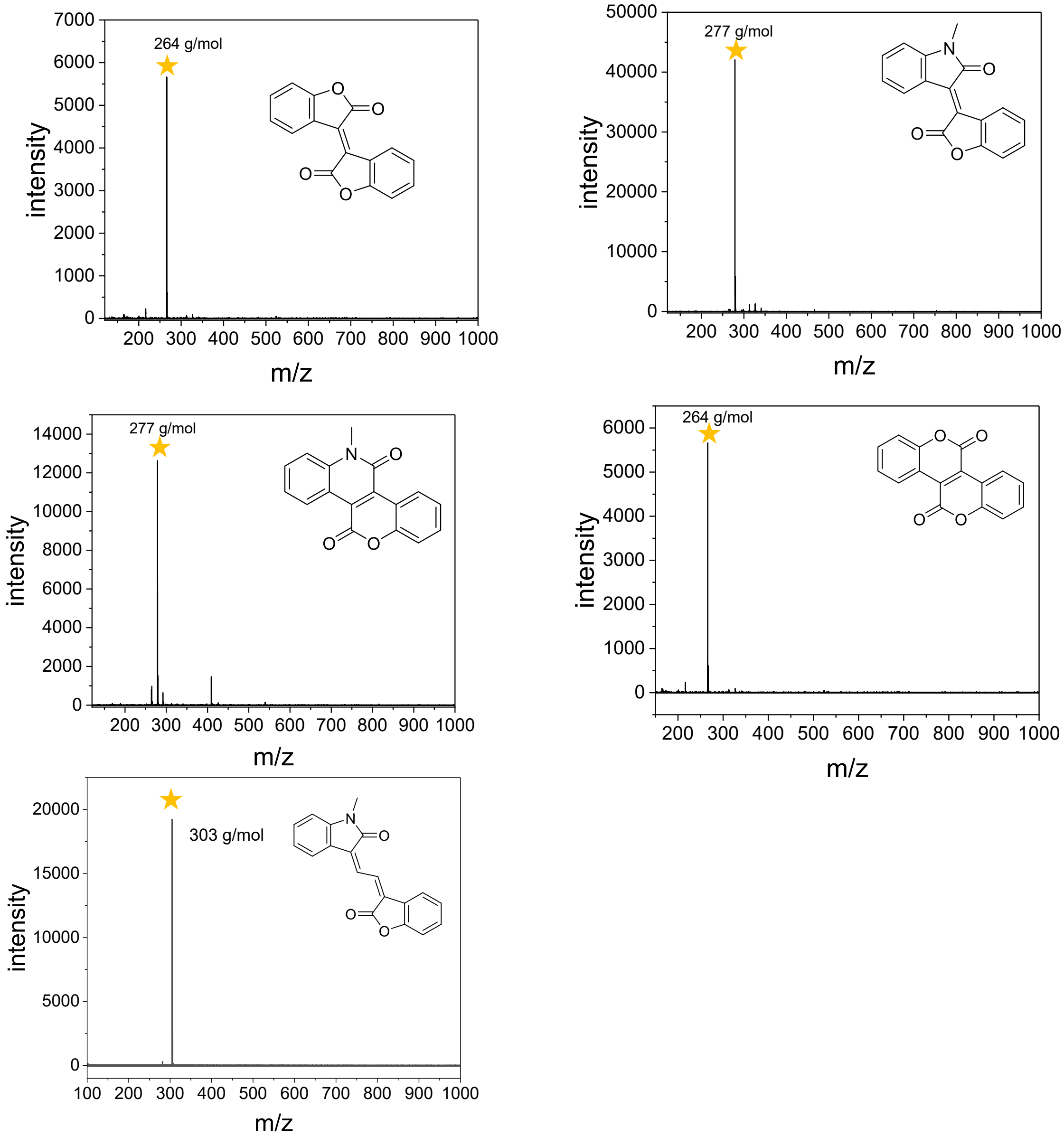

Figure S62. MALDI spectra of isolated fractions in model reaction studies.

6. X-ray diffraction

Table S2. Selected crystal and structural refinement data of **11** and **15–17**.

	**11**	**15**	**16**	**17**
Empirical formula	$C_{17}H_{11}NO_3$	$C_{18}H_{14}N_2O_2$	$C_{21}H_{22}N_2O_3$	$C_{19}H_{13}NO_3$
Formula mass (g·mol^{-1})	277.27	290.31	350.40	303.30
Crystal system	orthorhombic	orthorhombic	triclinic	orthorhombic
Space group	*Pna2₁*	*Pna2₁*	*P-1*	*Pbca*
a (Å)	27.9926(10)	18.4388(5)	9.0634(10)	6.7716(2
b (Å)	18.9491(7)	4.6249(1)	11.2134(14)	18.0735(6)
c (Å)	4.7125(1)	15.6887(4)	18.1078(17)	22.8000(8)
α (°)	90	90	90.204(9)	90
β (°)	90	90	101.989(8)	90
γ (°)	90	90	93.164(11)	90
V (Å^3)	2499.67(14)	1337.89(6)	1797.2(3)	2790.41(16)
Z	8	4	4	8
D_{calc} (Mg·m^{-3})	1.474	1.441	1.295	1.444
Wavelength (Å)	1.54178	1.54178	1.54178	1.54178
Temperature (K)	100	100	100	100
Absorption coefficient (mm^{-1})	0.839	0.772	0.703	0.803
F(000)	1152	608	744	1264
Reflection collected	20881	27525	39316	39415
Reflection unique / R_{int}[a)]	4885 / 0.0710	2721 / 0.0927	4959 / 0.0523	20700 / 0.0557
Goodness-of-fit on F^2 [b)]	1.104	1.062	1.065	1.036
Data / restrains / parameters	4885 / 1 / 382	2721 / 1 / 202	7324 / 2 / 483	2852 / 0 / 210
θ range for data collection (°)	2.816 to 72.187	4.796 to 74.413	2.495 to 74.925	3.877 to 74.495
Limiting indices	$-33 \le h \le 34$,	$-23 \le h \le 23$,	$-11 \le h \le 11$,	$-7 \le h \le 8$,
	$-23 \le k \le 18$,	$-5 \le k \le 5$,	$-13 \le k \le 14$,	$-22 \le k \le 22$,
	$-5 \le l \le 5$	$-19 \le l \le 19$	$-22 \le l \le 22$	$-27 \le l \le 28$
Final R indices [$I > 2\sigma(I)$][c)]	$R_1 = 0.0778$, $wR_2 = 0.2005$	$R_1 = 0.0651$, $wR_2 = 0.1681$	$R_1 = 0.0785$, $wR_2 = 0.2007$	$R_1 = 0.0356$, $wR_2 = 0.0909$
R indices (all data)[c)]	$R_1 = 0.0954$, $wR_2 = 0.2145$	$R_1 = 0.0696$, $wR_2 = 0.1756$	$R_1 = 0.1088$, $wR_2 = 0.2185$	$R_1 = 0.0436$, $wR_2 = 0.0971$
Largest diff. peak/hole (e·Å^{-3})	0.805/-0.294	0.648/-0.255	0.399/-0.373	0.297/-0.184
CCDC	2516005	2516006	2516007	2516008

[a)] $R_{int} = \sum | F_0^2 - F_0^2(\text{mean}) | / \sum F_0^2$, where F_0^2 (mean) is the average intensity of symmetry equivalent diffractions. [b)] $S = [\sum w (F_0^2 - F_c^2)^2] / (n-p)^{1/2}$, where n = number of reflections, p = number o parameters. [c)] $R = [\sum(\| F_0 | - | F_c |) / \sum | F_0 |)$; $R[\sum(w(F_0^2 - F_c^2))^2 / \sum(wF_0^4)]^{1/2}$.

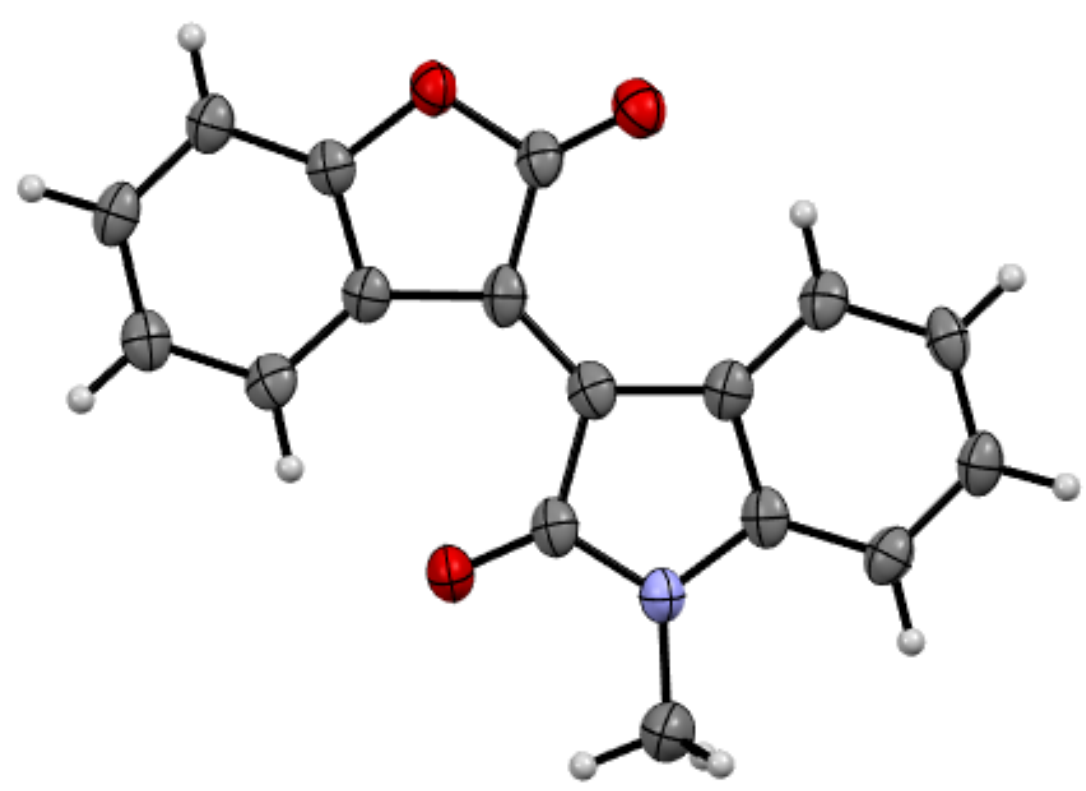

Figure S63. ORTEP (50 % probability ellipsoids) of the molecular structure of cross aldol product **11**.

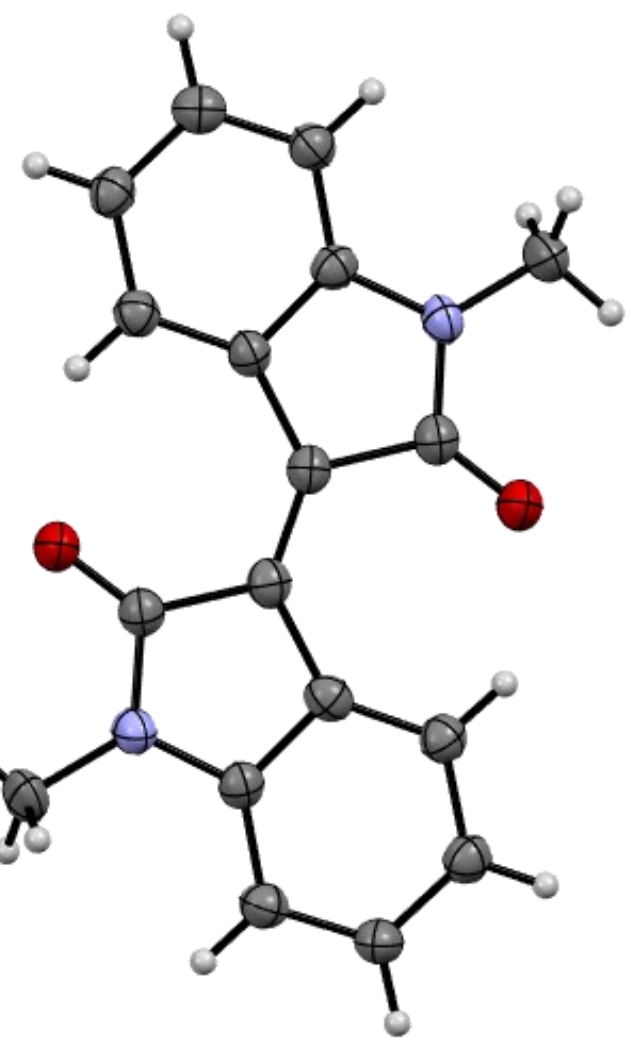

Figure S64. ORTEP (50 % probability ellipsoids) of the molecular structure of N,N'dimethyl isoindigo defect **15**.

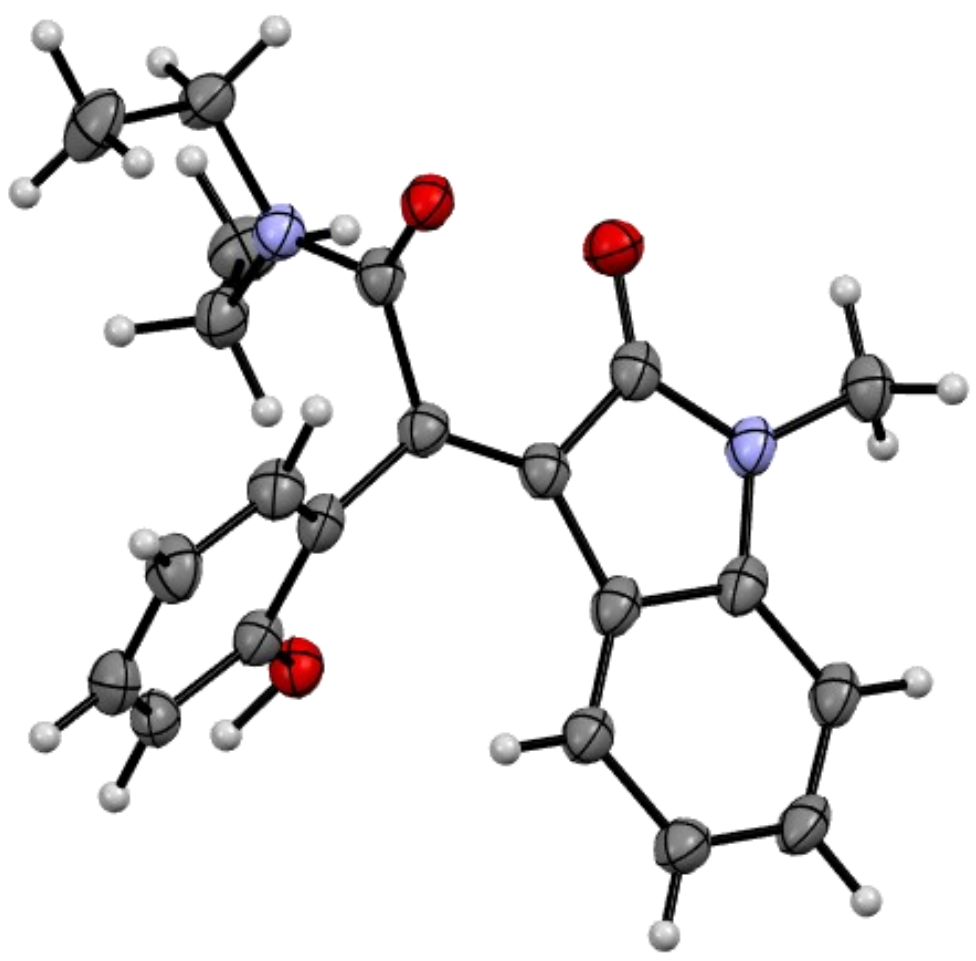

Figure S65. ORTEP (50 % probability ellipsoids) of the molecular structure of defect **16**.

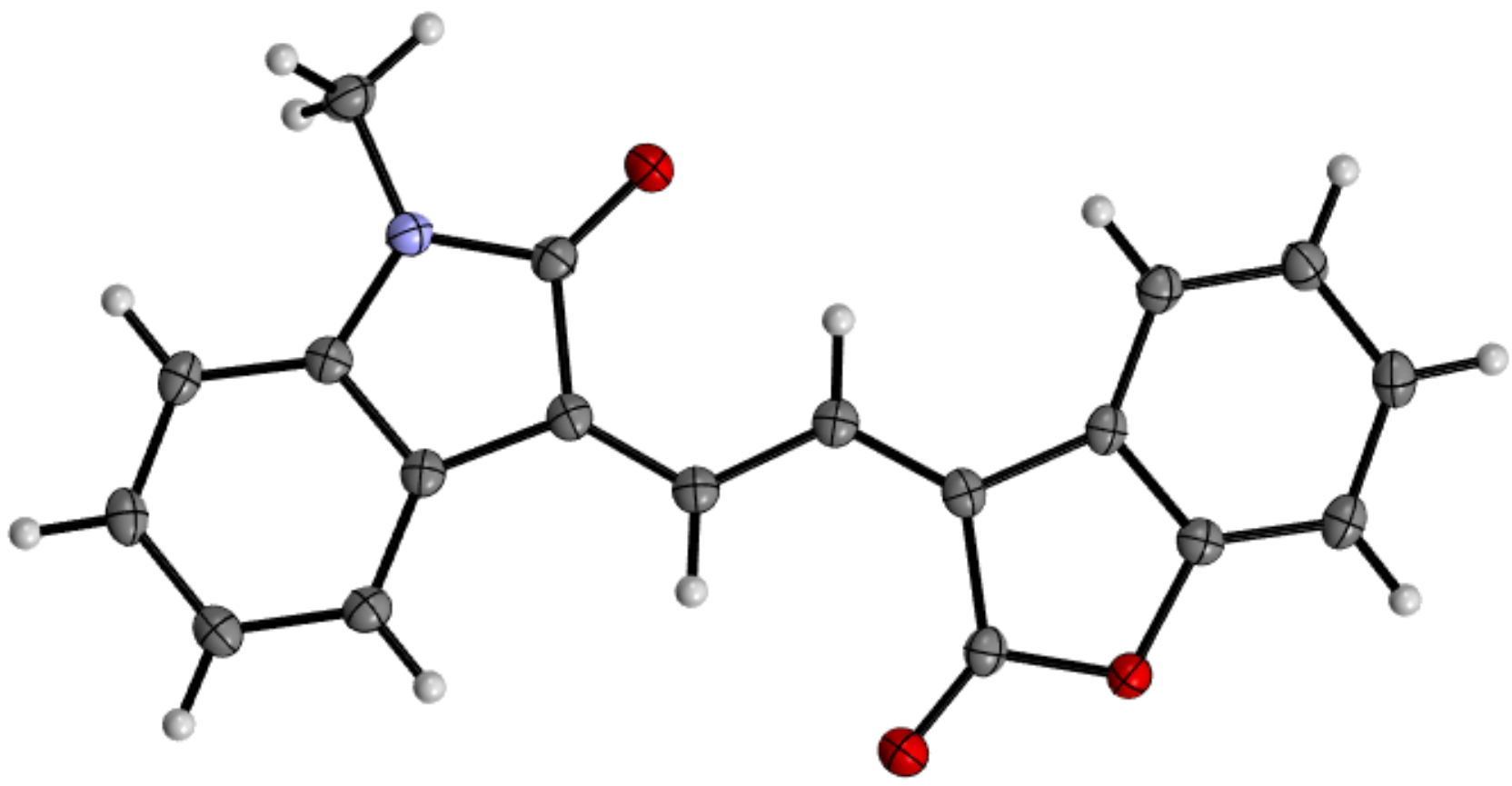

Figure S66. ORTEP (50 % probability ellipsoids) of the molecular structure of **17.**

7. References

(1) R. Hinojosa, D.; J. Pataki, N.; Rossi, P.; Erhardt, A.; Guchait, S.; Pallini, F.; McNeill, C.; Müller, C.; Caironi, M.; Sommer, M. Solubilizing Benzodifuranone-Based Conjugated Copolymers with Single-Oxygen-Containing Branched Side Chains. *ACS Appl. Polym. Mater.* **2024**, *6* (1), 457–465. https://doi.org/10.1021/acsapm.3c02137.

(2) Matsidik, R.; Komber, H.; Brinkmann, M.; Schellhammer, K. S.; Ortmann, F.; Sommer, M. Evolution of Length-Dependent Properties of Discrete n-Type Oligomers Prepared via Scalable Direct Arylation. *J. Am. Chem. Soc.* **2023**, *145* (15), 8430–8444. https://doi.org/10.1021/jacs.3c00058.

(3) Cardona, C. M.; Li, W.; Kaifer, A. E.; Stockdale, D.; Bazan, G. C. Electrochemical Considerations for Determining Absolute Frontier Orbital Energy Levels of Conjugated Polymers for Solar Cell Applications. *Advanced Materials* **2011**, *23* (20), 2367–2371. https://doi.org/10.1002/adma.201004554.

(4) Sheldrick, G. M. A Short History of *SHELX*. *Acta Crystallogr A Found Crystallogr* **2008**, *64* (1), 112–122. https://doi.org/10.1107/S0108767307043930.

(5) Sheldrick, G. M. Crystal Structure Refinement with SHELXL. *Acta Cryst C* **2015**, *71* (1), 3–8. https://doi.org/10.1107/S2053229614024218.

(6) Zhang, Z.; Ji, R.; Kroll, M.; Hofstetter, Y. J.; Jia, X.; Becker-Koch, D.; Paulus, F.; Löffler, M.; Nehm, F.; Leo, K.; Vaynzof, Y. Efficient Thermally Evaporated γ-CsPbI3 Perovskite Solar Cells. *Advanced Energy Materials* **2021**, *11* (29), 2100299. https://doi.org/10.1002/aenm.202100299.

(7) Ji, R.; Zhang, Z.; Hofstetter, Y. J.; Buschbeck, R.; Hänisch, C.; Paulus, F.; Vaynzof, Y. Perovskite Phase Heterojunction Solar Cells. *Nat Energy* **2022**, *7* (12), 1170–1179. https://doi.org/10.1038/s41560-022-01154-y.

(8) Becker-Koch, D.; Rivkin, B.; Paulus, F.; Xiang, H.; Dong, Y.; Chen, Z.; Bakulin, A. A.; Vaynzof, Y. Probing Charge Transfer States at Organic and Hybrid Internal Interfaces by Photothermal Deflection Spectroscopy. *J. Phys.: Condens. Matter* **2019**, *31* (12), 124001. https://doi.org/10.1088/1361-648X/aafa4e.

(9) Hanwell, M. D.; Curtis, D. E.; Lonie, D. C.; Vandermeersch, T.; Zurek, E.; Hutchison, G. R. Avogadro: An Advanced Semantic Chemical Editor, Visualization, and Analysis Platform. *J Cheminform* **2012**, *4* (1), 17. https://doi.org/10.1186/1758-2946-4-17.

(10) *LMAPper - The SPM and Mol Viewer Reviews - 2026*. https://sourceforge.net/projects/spm-and-mol-viewer/reviews/ (accessed 2026-04-08).

(11) Moro, S.; Siemons, N.; Drury, O.; Warr, D. A.; Moriarty, T. A.; Perdigão, L. M. A.; Pearce, D.; Moser, M.; Hallani, R. K.; Parker, J.; McCulloch, I.; Frost, J. M.; Nelson, J.; Costantini, G. The Effect of Glycol Side Chains on the Assembly and Microstructure of Conjugated Polymers. *ACS Nano* **2022**, *16* (12), 21303–21314. https://doi.org/10.1021/acsnano.2c09464.

(12) Calaminici, P.; Janetzko, F.; Köster, A. M.; Mejia-Olvera, R.; Zuniga-Gutierrez, B. Density Functional Theory Optimized Basis Sets for Gradient Corrected Functionals: 3d Transition Metal Systems. *The Journal of Chemical Physics* **2007**, *126* (4), 044108. https://doi.org/10.1063/1.2431643.

(13) Perdew, J. P.; Burke, K.; Ernzerhof, M. Generalized Gradient Approximation Made Simple. *Phys. Rev. Lett.* **1996**, *77* (18), 3865–3868. https://doi.org/10.1103/PhysRevLett.77.3865.

(14) Johnson, J. R.; Ketcham, R. Thiazolothiazoles. I. The Reaction of Aromatic Aldehydes with Dithioöxamide1. *J. Am. Chem. Soc.* **1960**, *82* (11), 2719–2724. https://doi.org/10.1021/ja01496a017.

(15) *Thiazolothiazoles. I. The Reaction of Aromatic Aldehydes with Dithioöxamide1 | Journal of the American Chemical Society*. https://pubs.acs.org/doi/10.1021/ja01496a017 (accessed 2025-07-07).
(16) Wang, C.-P.; Jiang, G.-F. An Efficient Method Based on Indoles for the Synthesis of Isatins by Taking Advantage of I2O5 as Oxidant. *Tetrahedron Letters* **2017**, *58* (18), 1747–1750. https://doi.org/10.1016/j.tetlet.2017.03.060.
